# New *N*-Substituted-1,2,4-triazole Derivatives of Pyrrolo[3,4-*d*]pyridazinone with Significant Anti-Inflammatory Activity—Design, Synthesis and Complementary In Vitro, Computational and Spectroscopic Studies

**DOI:** 10.3390/ijms222011235

**Published:** 2021-10-18

**Authors:** Łukasz Szczukowski, Edward Krzyżak, Benita Wiatrak, Paulina Jawień, Aleksandra Marciniak, Aleksandra Kotynia, Piotr Świątek

**Affiliations:** 1 Department of Medicinal Chemistry, Wroclaw Medical University, Borowska 211, 50-556 Wroclaw, Poland; 2 Department of Inorganic Chemistry, Wroclaw Medical University, Borowska 211a, 50-556 Wroclaw, Poland; edward.krzyzak@umed.wroc.pl (E.K.); aleksandra.marciniak@umed.wroc.pl (A.M.); aleksandra.kotynia@umed.wroc.pl (A.K.); 3 Department of Pharmacology, Wroclaw Medical University, Mikulicza-Radeckiego 2, 50-345 Wroclaw, Poland; benita.wiatrak@umed.wroc.pl (B.W.); paulina.jawien@umed.wroc.pl (P.J.)

**Keywords:** cyclooxygenase, 1,2,4-triazole, pyridazinone, SAR, molecular docking, anti-inflammatory activity, antioxidant activity, ADME

## Abstract

Regarding that the chronic use of commonly available non-steroidal and anti-inflammatory drugs (NSAIDs) is often restricted by their adverse effects, there is still a current need to search for and develop new, safe and effective anti-inflammatory agents. As a continuation of our previous work, we designed and synthesized a series of 18 novel *N*-substituted-1,2,4-triazole-based derivatives of pyrrolo[3,4-*d*]pyridazinone **4a-c-9a-c**. The target compounds were afforded via a convenient way of synthesis, with good yields. The executed cell viability assay revealed that molecules **4a-7a**, **9a**, **4b-7b**, **4c-7c** do not exert a cytotoxic effect and were qualified for further investigations. According to the performed in vitro test, compounds **4a-7a**, **9a**, **4b**, **7b**, **4c** show significant cyclooxygenase-2 (COX-2) inhibitory activity and a promising COX-2/COX-1 selectivity ratio. These findings are supported by a molecular docking study which demonstrates that new derivatives take position in the active site of COX-2 very similar to *Meloxicam*. Moreover, in the carried out in vitro evaluation within cells, the title molecules increase the viability of cells pre-incubated with the pro-inflammatory lipopolysaccharide and reduce the level of reactive oxygen and nitrogen species (RONS) in induced oxidative stress. The spectroscopic and molecular modeling study discloses that new compounds bind favorably to site II(m) of bovine serum albumin. Finally, we have also performed some in silico pharmacokinetic and drug-likeness predictions. Taking all of the results into consideration, the molecules belonging to series **a** (**4a-7a**, **9a**) show the most promising biological profile.

## 1. Introduction

The inflammatory response that leads to homeostasis restoration is provoked by various exogenous and endogenous harmful stimuli and inducers, such as injury, tissue malfunctioning or infection. Its course, purpose and aftermath depend on the trigger. Characteristic symptoms that occur in inflamed areas are edema, reddening, hypersensitivity and often pain, which plays an important warning and protective role and promotes the organism’s reflex and behavioral response to minimize the effects of tissue damage. The inflammation is a complicated process coordinated by a great variety of mediators whose expression and complex network of relationships are still not satisfactorily understood. Numerous proinflammatory mediators of different origins could be divided into the following groups: vasoactive amines, vasoactive peptides, fragments of complement components and lipid mediators, such as eicosanoids, cytokines, chemokines and proteolytic enzymes. A lot of those aforementioned agents, besides affecting the target cells and tissues, could also induce the production of other elements. The best possible understanding and explanation of the mechanisms responsible for inflammatory mediators’ expression, action and mutual dependence is essential in the effective management of different inflammatory diseases. Suitable pharmacological treatment and natural tissue healing processes cause acute pain and inflammation to disappear after some days. Nevertheless, the lack of or ineffective pharmacotherapy may trigger dangerous pathophysiological changes, which lead to the evolution of chronic inflammation and pain syndrome [1,2,3,4,5].

Most drugs commonly used in the treatment of pain and inflammatory disorders act as, mainly non-selective, inhibitors of both isoforms of cyclooxygenase (COX) and belong to a varied and spacious group of medicines named as non-steroidal and anti-inflammatory drugs (NSAIDs) [6,7,8,9,10]. The analgesic, antipyretic and anti-inflammatory effects associated with the administration of these medicaments are related to the reduction of COX-dependent prostaglandins (PGs) production, which belong, alongside with leukotrienes and lipoxins, to the above-mentioned group of eicosanoids [4,5,6,7,8,9,10]. Because PGs, except for the mediation of inflammation, play a crucial role in homeostasis maintenance and exert a protective effect, e.g., in the gastrointestinal and cardiovascular system, such decreased COX activity may also lead to dangerous side effects [6,7,11,12,13,14,15]. Usually, in patients receiving NSAIDs, adverse effects related to the gastroduodenal tract, such as heartburn, dyspepsia, stomach ache or even ulceration, may occur [11,12,13,14]. Initially, it was believed that a constitutive isoform named COX-1 is engaged in various physiological processes, while the development of pain and inflammation is under control of the mediators produced by the inducible form—COX-2 [6,7,8,9,10,13,15]. However, the introduction of selective COX-2 inhibitors—COXIBs, which were supposed to spare gastric mucosa, have quickly disproved this theory. Although any significant harmful impact of COXIBs on the gastrointestinal tract has not been noticed, the therapy with those drugs has been shown to carry a serious risk of hazardous cardiovascular incidents, which can even lead to patient death. Consequently, some COXIBs have been withdrawn from the market, and among them, the case of *Rofecoxib* became the most shameful [11,12,13,14,15,16,17].

Therefore, there is still a current need to search for and develop new, safe and efficient analgesic and anti-inflammatory compounds, since severe adverse effects often restrict the long-term usage of already known and available NSAIDs [11,12,13,14]. New drug candidates with potential application in the treatment of various inflammatory disorders can be received either through the structural alteration of already known NSAIDs, such as *Diclofenac* [18], *Naproxen* [19], *Celecoxib* [20], *Ibuprofen* [21], or by developing fully novel classes of cyclooxygenase inhibitors. When considering the design of new anti-inflammatory agents, one of the most popular and effective synthetic approaches in contemporary medicinal chemistry relies on replacing the free carboxylic group with different bioisosteric five-membered heterocyclic rings, such as 1,3,4-oxadiazole [18,19,20,21,22], 1,3-thiazole [23,24,25], pyrazole [26,27] or 1,2,4-triazole [28,29,30,31,32,33]. According to the leading investigations, such a strategy can be successfully applied to modify widespread used NSAIDs and other promising compounds not introduced in the market yet. As a result, potent cyclooxygenase inhibitors with an improved affinity towards COX-2 isoform and reduced gastrotoxicity can be received [18,19,22,23,30,31].

In our former studies, we have reported the synthesis and comprehensive biological evaluation of new 1,3,4-oxadiazole-based derivatives of pyrrolo [3,4-*d*]pyridazinone designed as a new class of COX inhibitor. The investigated compounds exerted promising in vitro cyclooxygenase inhibitory activity and acted as specific or selective COX-2 inhibitors. It is worth emphasizing that each examined molecule showed a superior COX-2/COX-1 selectivity ratio than *Meloxicam*, which was used as a reference drug. These findings were supported by the results of molecular docking studies, which revealed that tested 1,3,4-oxadiazole derivatives of pyrrolo[3,4-*d*]pyridazinone take place in the active site of cyclooxygenase very similar to that of *Meloxicam* [34,35]. Subsequently, the most potent molecules have been investigated in vivo. It has been demonstrated by the enzyme-linked immunosorbent assay (ELISA) tests that the measured concentrations of inflammatory mediators—prostaglandin E_2_ (PGE_2_) and myeloperoxidase (MPO) in mice serum—were decreased after the application of our derivatives. Moreover, the macro- and microscopic histopathological assessment of gastric mucosa proved that novel compounds caused negligible stomach lesions andno histopathological changes were observed. These results confirmed the safe gastric profile of investigated molecules [36].

Encouraged by those promising results, we have decided to modify the structure of the above-mentioned derivatives by replacing the 1,3,4-oxadiazole with 4-substituted-1,2,4-triazole pharmacophore to obtain even more effective compounds. The introduction of this five-membered ring was inspired by the leading research described in the most recent literature [28,29,30,31,32,33]. The 1,2,4-triazole is an important moiety present in numerous potent bioactive molecules with a wide range of therapeutic applications [28,29,30,31,32]. It also acts as a valuable building block used in the design and synthesis of promising analgesic and anti-inflammatory agents, especially those with a good affinity towards an inducible COX-2 isoform [28,30,31,32]. Needless to say, different five-membered heterocycles serve as the structural core of the previously mentioned selective COX-2 inhibitors—COXIBs [17,20,25]. Considering that the binding pocket of COX-2 isoenzyme is bigger than that of COX-1 [6,7,8,9,10], we expect that the presence of expanded 4-substituted-1,2,4-triazole residues will enhance the COX-2 selectivity of titled compounds.

In summing up, here we report herein the design, synthesis and complex in vitro and in silico investigations of a new series of 4-substituted-1,2,4-triazole-based derivatives of pyrrolo[3,4-*d*]pyridazinone. First of all, we have determined the cytotoxic effect of the new compounds. After excluding toxic derivatives from further studies, we have defined the potential affinity and binding mode of the novel structures to both isoforms of cyclooxygenase. The investigations were carried out using in vitro enzymatic assay and computational studies, as well. Afterwards, have we estimated the antioxidant activity of the title compounds. Finally, the interaction binding manner of novel 1,2,4-triazole-based derivatives of pyrrolo[3,4-*d*]pyridazinone with bovine serum albumin (BSA) has been established. All of the performed experiments focused on the most precise determination of the possible mechanism of action and pharmacokinetic properties of new molecules and their potential application in the treatment of inflammatory disorders.

## 2. Results

### 2.1. Chemistry

**Scheme 1:**
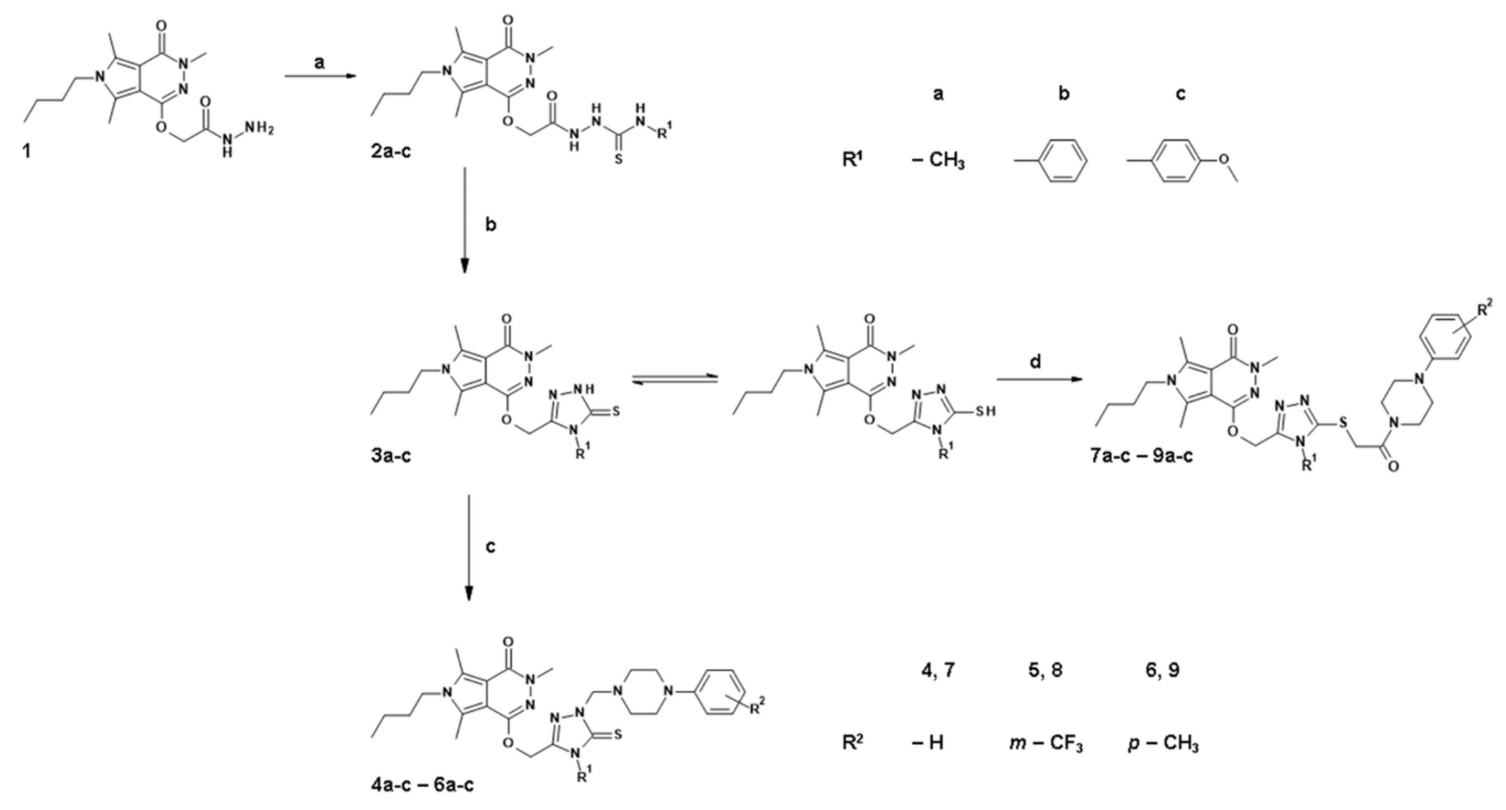
Synthesis of the intermediates **2a-c-3a-c** and the title compounds **4a-c-9a-c**. Reagents and reaction conditions: (a) N substituted isothiocyanate, ethanol, reflux, 0.5–2 h; (b) I. 5% NaOH (aq), reflux, 2 h; II. Cooling, crushed ice, stirring, acidification with 7.5% HCl; (c) 37% HCHO, 4 aryl piperazine derivative, methanol, RT 5 h; (d) C_2_H_5_ONa, 1 (2-chloro-1-oxoethyl)-4-aryl piperazine derivative, ethanol, reflux, 5 h.

The aim of the present study was the design and synthesize a new series of *N*-substituted-1,2,4-triazole derivatives of pyrrolo[3,4-*d*]pyridazinone. Scheme 1 presents all of the performed chemical modifications and structures of novel molecules (each new compounds’ structural formula is also presented in Table S1 in Supplementary Data). The thin layer chromatography (TLC) technique was used to monitor the progress of the reaction. The structure and purity of all of the new derivatives were established and confirmed based on the spectra analysis: ^1^H and ^13^C nuclear magnetic resonance (NMR), Fourier transform infrared (FT-IR), electrospray ionization mass spectrometry (ESI-MS). All of the analytical and spectroscopic properties of every newly obtained compound were in good agreement with their predicted structures. The Experimental Section and Supplementary Data provide detailed information concerning the analytical and spectroscopic data, reactants, solvents and reactions environment. The synthetic pathway and structure of the starting molecule 2-(6-butyl-3,5,7-trimethyl-4-oxo-pyrrolo[3,4-*d*]pyridazin-1-yl)oxyacetohydrazide 1 has already been published in our previous paper [34].

The first step of the current synthesis relied on forming adequate *N-*substituted-(aminothioxomethyl)hydrazides **2a-c**. The aforementioned hydrazide **1** was refluxed in ethanol in the presence of the appropriate *N*-substituted isothiocyanate for 30 to 120 min. The mixture was then cooled down, and the formed precipitate was filtered off, washed thoroughly with ethanol and purified by crystallization from this solvent. Compounds **2a-c** were obtained with a good to excellent yield (up to 94%). Their formation was confirmed by spectral analysis. When considering the ^1^H NMR spectra of molecules **2a-c**, a characteristic peak of protons linked to *N*1, *N*2 and *N*3 nitrogens of thiosemicarbazide moiety were recorded as three singlets in the range of δ 7.89–10.18 ppm. The methylene group connecting the pyrrolo[3,4-*d*]pyridazinone core and hydrazine residue appears as two proton singlets within δ 4.70-4.76 ppm in the ^1^H NMR and as a signal about δ 63.74-63.84 ppm in the ^13^C NMR spectra, respectively. Moreover, in the ^13^C NMR spectra of compounds **2a-c**, a signal near δ 182 ppm is assigned to the carbon atom forming the C=S bond. Finally, in both the ^1^H and ^13^C NMR spectra of structures **2a-c**, new peaks characteristic for the methyl group in series **a** and phenyl or 4-methoxyphenyl substituent in series **b** and **c** can be distinguished.

Subsequently, the synthesis of key 1,2,4-triazole derivatives of pyrrolo[3,4-*d*]pyridazinone **3a-c** was carried out. First, compounds **2a-c** underwent alkaline cyclization by refluxing in a 5% aqueous sodium hydroxide solution for about 2 h. Then, the reaction mixture was poured onto crushed ice and acidified with 7.5% hydrochloric acid solution, affording the corresponding *N*-substituted-1,2,4-triazoles **3a-c**. Finally, the formed white or yellowish precipitate of compounds **3a-c** was filtered off, washed with cold water and recrystallized from a proper solvent. In reference to the NMR spectra of structures **3a-c**, the change in the chemical shift of the signal of methylene linker can be easily observed. The signal of the protons of this group is shown at δ 5.12–5.30 ppm in the ^1^H NMR, while the signal of a carbon atom is recorded about δ 57.85–58.09 ppm in the ^13^C NMR spectra, accordingly. Moreover, the presence of the distinctive peak near δ 168.04–169.12 ppm in the ^13^C NMR spectra, which is identified with the triazole carbon forming C=S bond, alongside with the signal characteristic for a *N*H proton observed in the range of δ 13.82–14.04-ppm in the ^1^H NMR spectra may suggest that derivatives **3a-c** occur in the thione form.

The final stage of the planned synthesis relied on the formation of titled *N*-substituted-1,2,4-triazole derivatives of pyrrolo[3,4-*d*]pyridazinone **4a-c-9a-c**. As it has already been mentioned, the concept of their structure was inspired by the leading literature data and our own previous investigations. As it was depicted on Scheme 1, the final molecules **4a-c-9a-c** could be divided into two series.

Compounds **4a-c-6a-c** are the new Mannich base-type derivatives of pyrrolo[3,4-*d*]pyridazinone based 1,2,4-triazoles. These molecules were obtained via an effective and convenient one-step reaction, carried out at room temperature. The corresponding derivative **3a-c** was stirred with the appropriate 4-aryl piperazine derivative and formaldehyde in methanol for several hours and left overnight. The distinctive two-proton singlet in the ^1^H NMR spectrum observed about δ 5.15–5.29 ppm. The signal at around δ 69.39–69.66 ppm in the ^13^C NMR spectrum clearly indicates the creation of the methylene linker, characteristic for Mannich bases. What is obvious, in both the ^1^H and ^13^C NMR spectra of compounds **4a-c-6a-c**, are the signals assigned to aryl piperazine pharmacophore have been recorded.

On the other hand, the structure of the final compounds **7a-c-9a-c** was inspired by the pharmacophore theory featured by Dogruer [37]. Therefore, in the case of these derivatives, the 4-aryl piperazine moiety is connected with a five-membered 1,2,4-triazole ring via a flexible 2-oxoethylene linker. First of all, suitable 2-chloro-1-oxoethylaryl piperazine derivatives were afforded according to the synthetic protocols which have already been reported [38]. Due to the occurrence of possible tautomerism in the mentioned five-membered ring, the alkylation of 1,2,4-triazole analogue of pyrrolo[3,4-*d*]pyridazinone **3a-c** with 2-chloro-1-oxoethylaryl piperazine derivative may result in the formation of a mixture of *N*- and *S*-isoforms. Based on our former study, we have engaged the same synthetic conditions [35]. Therefore, the title compounds **7a-c-9a-c** were obtained by refluxing the 4-substituted-1,2,4-triazole derivatives of pyrrolo[3,4-*d*]pyridazinone **3a-c** with the appropriate 2-chloro-1-oxoethylaryl piperazine for several hours in ethanol in the presence of sodium ethoxide (Scheme 1). The crude products were filtered off, washed thoroughly with ethanol and purified by crystallization from this solvent. The lack of characteristic peak in the ^13^C NMR spectra observed about δ 169.72–170.78 ppm, which was assigned to a carbon atom, forming a C=S bond (Experimental Section, Supplementary Data), strongly suggests that the final compounds **7a-c-9a-c** were formed via *S*-alkylation of 1,2,4-triazole derivatives of pyrrolo[3,4-*d*]pyridazinone **3a-b** (Scheme 1). This claim is supported by the presence of a distinctive signal shown near δ 152.25–152.62 ppm in the ^13^C NMR spectra of **7a-c-9a-c**, which can be identified with carbon atom C3 in 1,2,4-triazole ring binding sulphur atom via a single bond (Ar-C-*S*-CH_2_). Furthermore, a peak recorded about δ 165.49–165.72 ppm is typical for carbon atoms in carbonyl moiety (C=O). On the other hand, the signal which occurs near δ 41.98–42.23 ppm is assigned to carbon atom C1 (-CH_2_-) of the 2-oxoethylene linker. Moreover, the two-proton singlet in the range of δ 4.37–4.42 ppm in the ^1^H NMR spectra of **7a-c-9a-c** is assigned with the protons of carbon atom C1 in the mentioned linker. More detailed information is provided in the Experimental Section and Supplementary Data.

### 2.2. Evaluation of Viability

To estimate the effect of the new compounds on normal cells, a 3-(4, 5-Dimethylthiazol-2-yl)-2, 5-diphenyltetrazolium bromide (MTT) test was performed according to ISO 10993 part 5 Appendix C. The percentage of survival and the assessment of changes in the morphology of normal human dermal fibroblasts (NHDF) after contact with the tested compounds are presented in Table 1. The derivatives with 4-phenylpiperazine moiety, **4a-c** and **7a-c** and compounds **5a**, **6a**, **9a**, did not reduce the viability of NHDF cells below 70% in the tested concentration ranges. In the case of these molecules, no significant differences in cell survival were observed at a concentration of 100 µM. However, NHDF cell survival after 24-hour incubation was less than 50% for compounds **8a**, **8b**, **8c**, **9b** and **9c**, and the concentration at which 50% cell survival was observed has been calculated for these molecules. Therefore, these derivatives were excluded from further experiments. Furthermore, due to the low level of cytotoxicity (less than 30% of dead cells compared to the control—culture without test compounds in a complete medium only), the theoretical IC_50_ values for compounds **5b**, **6b**, **5c** and **6c** were calculated. In all of the cases, it was noted that an increase in cell viability was observed at the reduced concentration (10 and 50 µM).

On the microscopic image, few granules were observed in the NHDF cells in the tested concentration range for the compounds **4a-7a**, **9a**, **4b**, **7b**, **4c** and **7c**. Cell shrinkage and separation from the surface of the culture wells were not observed. These changes were classified according to the criterion of grade 1—low toxicity. Similar changes in the cell morphology were observed in the systems containing 100 µM for **5b**, **6b** and **5c**. In this case, a slightly greater number of endoplasmic granules were observed without any effect on the cell contraction. In this case, low toxicity was considered as well.

With reference to derivative **6c** at a concentration of 100 µM, the cells in the range of 10–15% contracted and detached from the medium. The appearance of fine granules inside the cytoplasmic cells was observed. However, at the reduced concentration of the tested compounds from individual endoplasmic pellets, a culture density comparable to that of the control culture was observed. Cell lysis was not observed. Regardless of the tested derivatives, there was no significant change in the NHDF cells compared to the control.

### 2.3. Cyclooxygenase (COX-1, COX-2) Inhibition Studies

#### 2.3.1. In Vitro COX Inhibition Assay

Inhibition of COX-1 and COX-2 activity of the tested compounds was assessed after 2 min incubation at 100 µM concentration using the Cayman’s COX Colorimetric Inhibitor Screening Assay Kit (Cat # 701050). The IC_50_ values and the COX-2/COX-1 selectivity ratios were calculated for each investigated and reference compound (*Meloxicam*, *Celecoxib* and *Diclofenac*). The results are shown in Table 2. The derivatives **4a-7a**, **9a**, **4b**, **7b** and **4c** revealed COX-2 inhibitory activity comparable to *Meloxicam*. Four of the studied molecules: **5a**, **7a**, **4b** and **7b**, also inhibited the constitutive isoform COX-1, but this activity was significantly lower. Compounds **5b**, **6b**, **5c-7c** did not exert any cyclooxygenase inhibition profile in the performed in vitro investigations. The analysis of these data allows us to conclude that the derivatives of series **a**, with methyl substituent in the 1,2,4-triazole ring, demonstrate possibly the best COX inhibitory activity. Better affinity towards COX-2 can be explained by the bigger binding pocket of that isoenzyme. Large and expanded molecules, like those from series **b** and **c**, with aryl substituent in the 1,2,4-triazole moiety, revealed poor or no inhibitory activity towards COX.

#### 2.3.2. Molecular Docking Study

The molecular docking analysis was carried out to explore the binding interactions of the tested compounds inside the active site of cyclooxygenase. The binding free energy (ΔG°) for the interaction with COX-1 for all of the studied compounds showed positive value. The energies obtained for the interactions with COX-2 are presented in Table 3. The ΔG° is negative for all of the compounds from series **a**, and for **4b**, **7b** and **4c**. The lowest value was found for compounds number **4** in each series (**4a-c**). The energies ΔE_2_, ΔE_3_ indicate that the main interactions are van der Waals and hydrogen bonding. The size of the COX-2 pocket is bigger than COX-1, which allows for the selective binding of larger molecules. However, the analyzed compounds are relatively large, especially the compounds from the **b** and **c** series, with phenyl and 4-methoxyphenyl rings. It can make some difficulties in the efficient docking to even COX-2.

**Figure 1:**
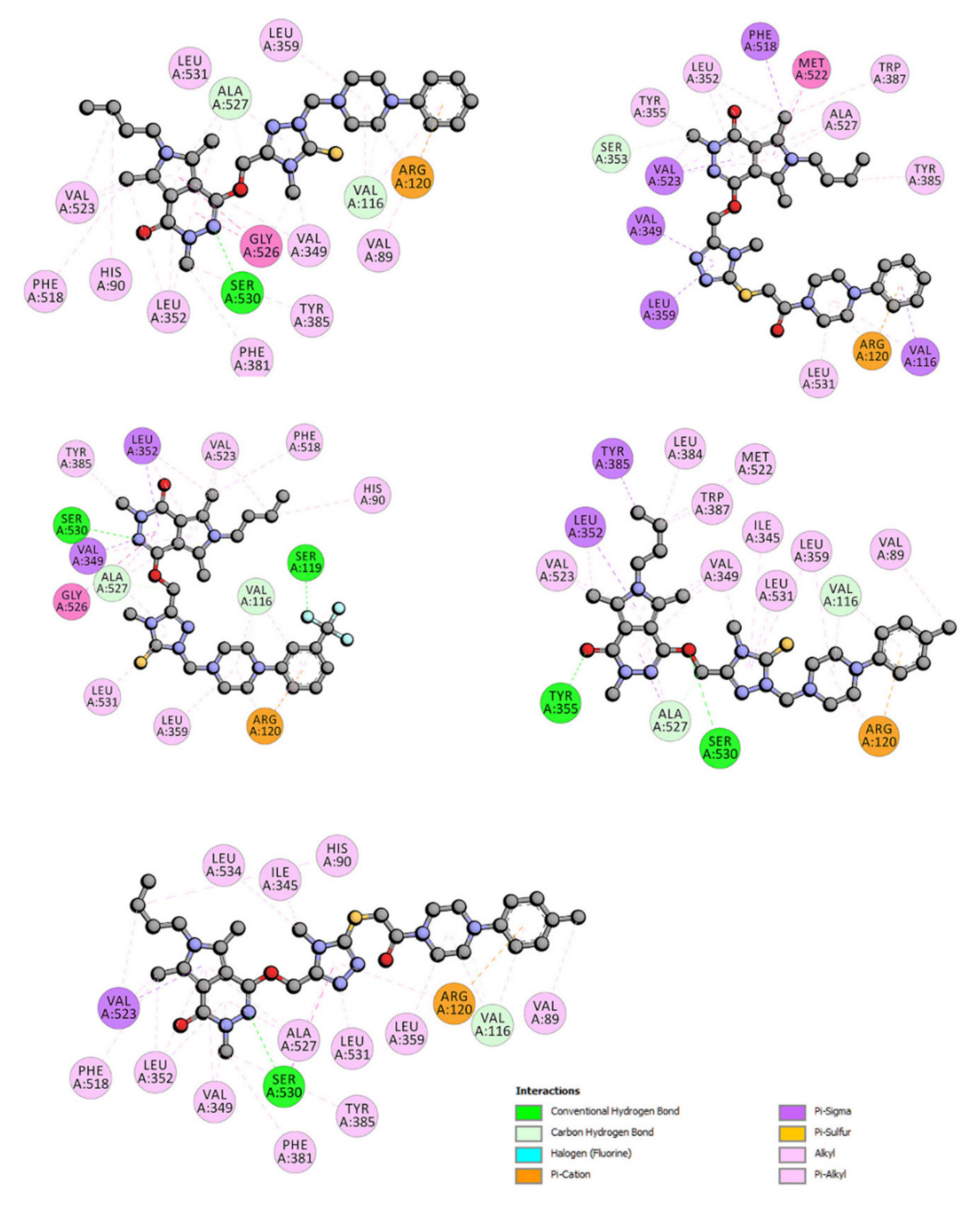
Two-dimensional interaction plot of **4a-7a**, **9a** with COX-2.

**Figure 2:**
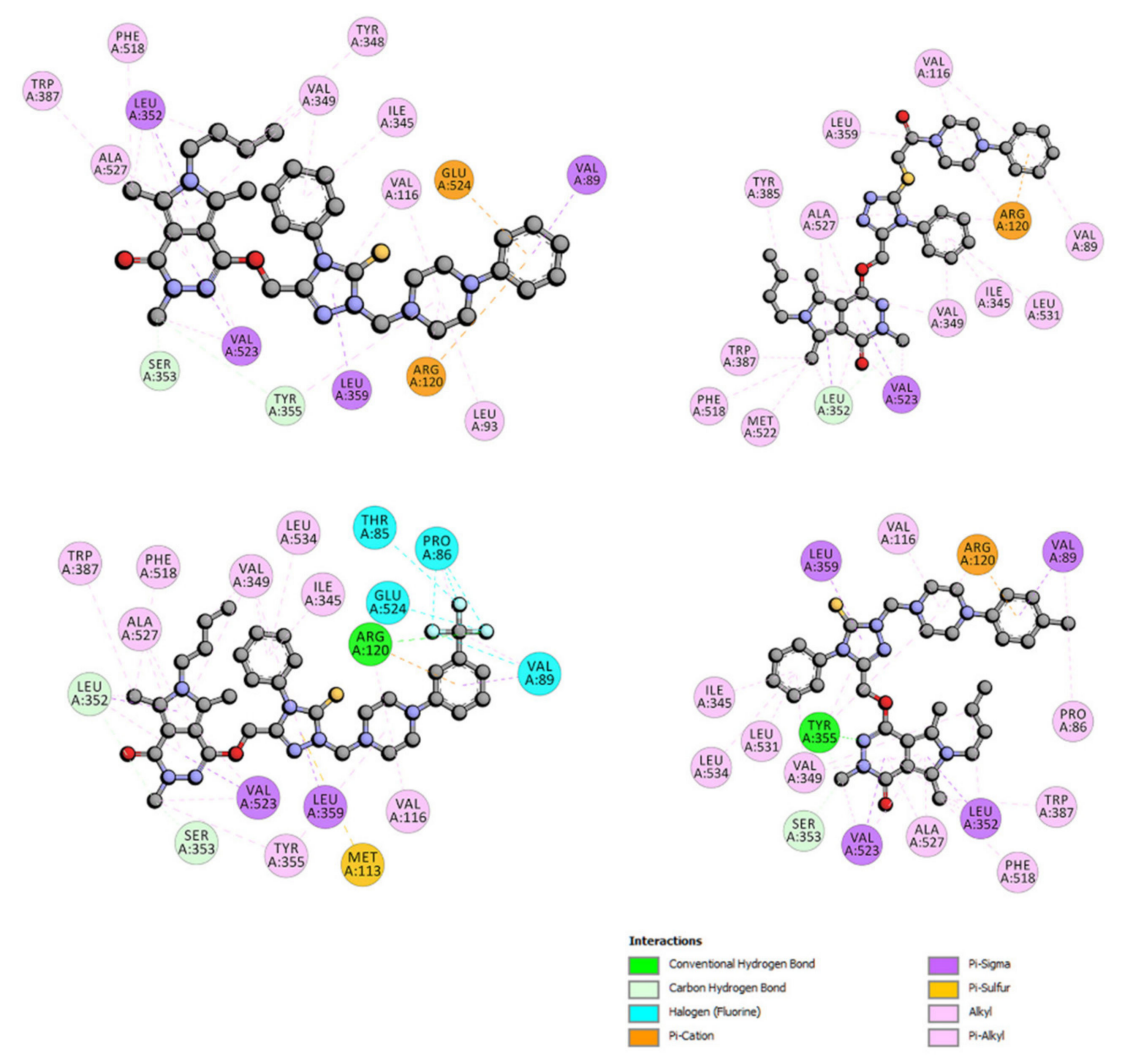
Two-dimensional interaction plot of **4b-7b** with COX-2.

**Figure 3:**
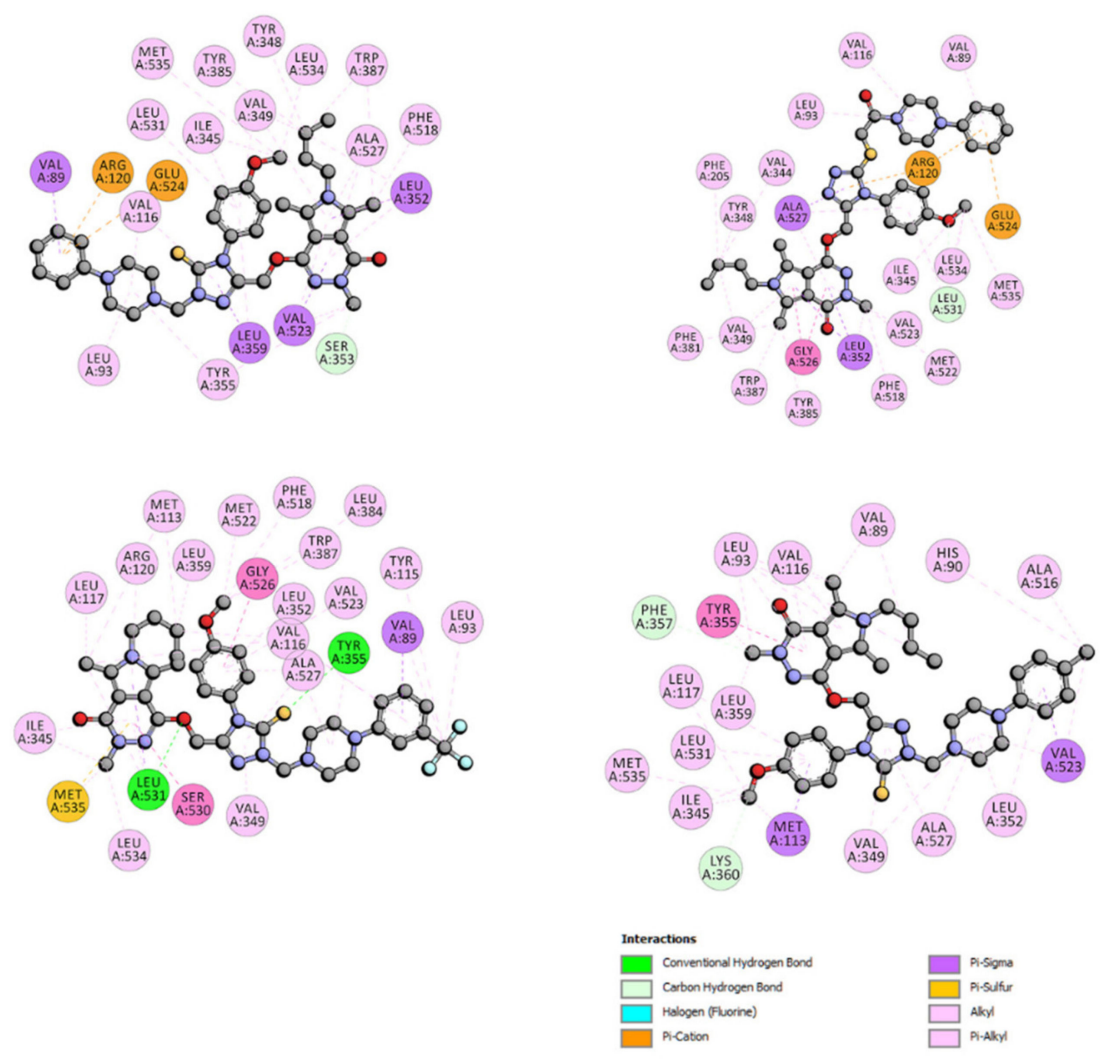
Two-dimensional interaction plot of **4c-7c** with COX-2.

**Figure 4:**
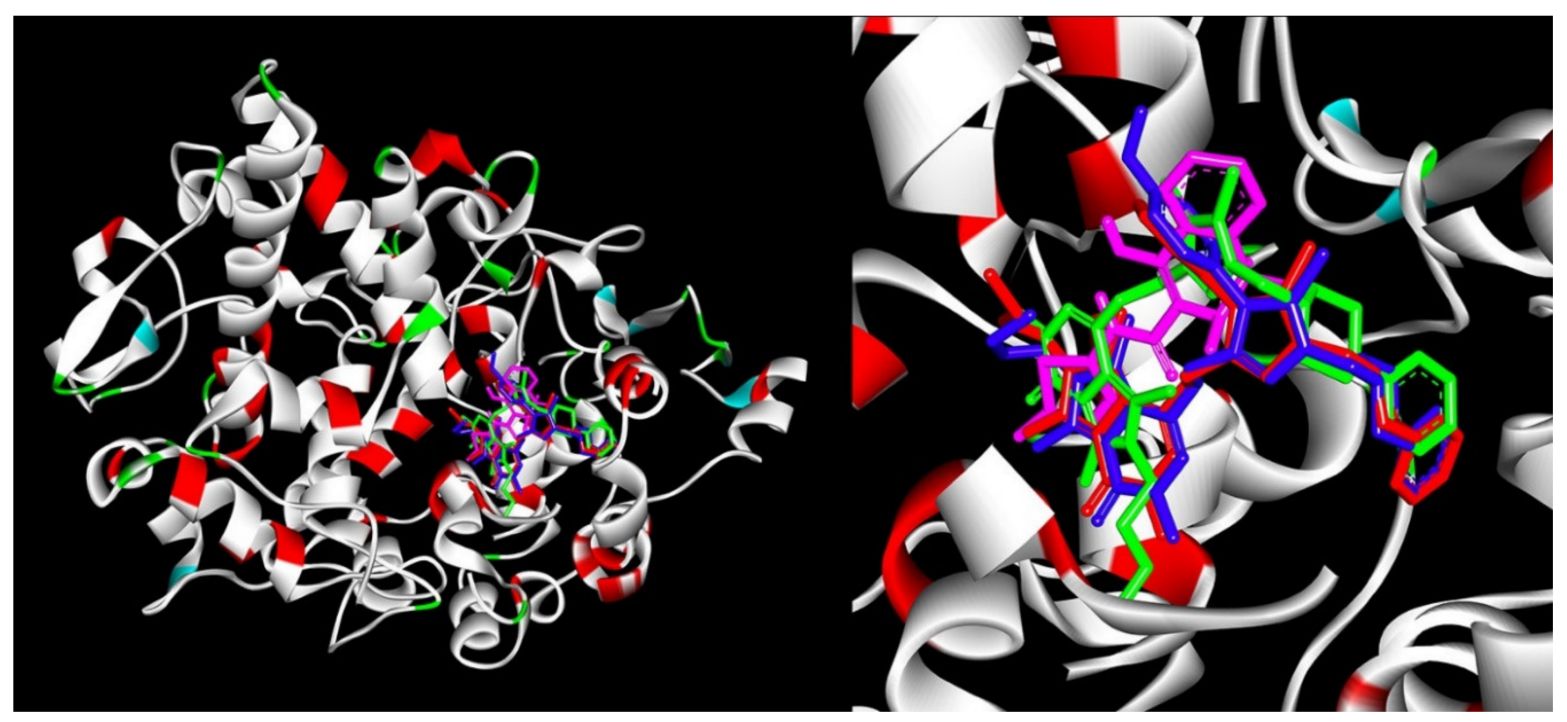
Docking poses of **4a** (green), **4b** (red), **4c** (blue) and *Meloxicam* (pink) inside COX-2.

Inside the COX-2 active site, some hydrogen bonds interactions were found, especially for the compounds belonging to series **a**: SER530, TYR355, ARG120, SER120. Moreover, various kinds of π interactions are observed. The details are presented in Figure 1, Figure 2 and Figure 3. In addition, the position of the studied compounds was found very similar to *Meloxicam* (Figure 4).

### 2.4. Anti-Inflammatory and Antioxidant Activity within Cells

The titled *N*-substituted-1,2,4-triazole derivatives of pyrrolo[3,4-*d*]pyridazinone were further evaluated for their anti-inflammatory potential in NHDF cells pre-incubated with the proinflammatory agent lipopolysaccharide (LPS) at a concentration of 50 µg/mL.

**Figure 5:**
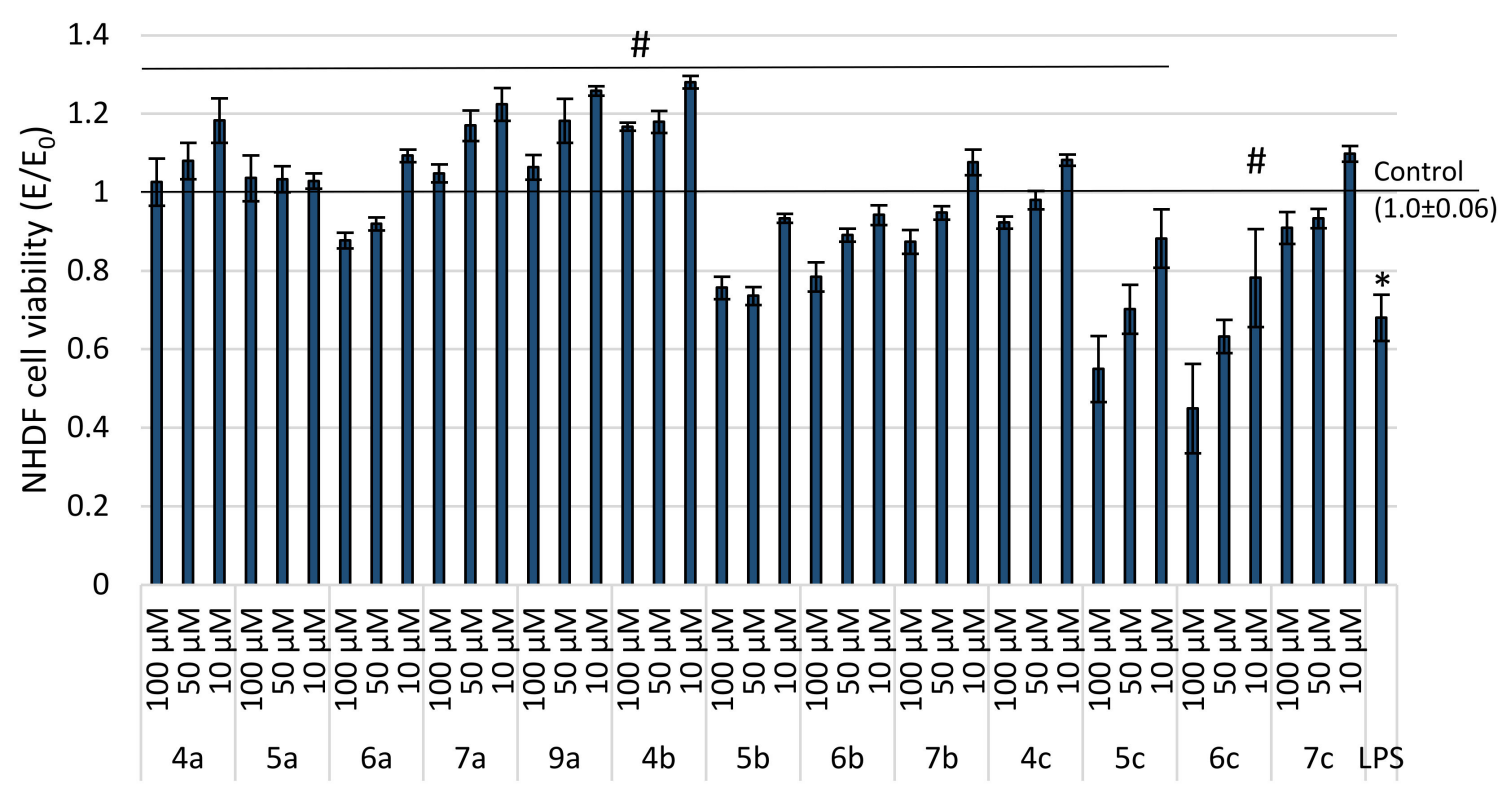
Impact of investigated compounds on NHDF cells after incubation with 50 µg/mL LPS measured through MTT assay; # *p* < 0.05—significant difference compared to the control with 50 μg/mL LPS and without compounds; * *p* < 0.05—significant difference compared to a negative control without 50 μg/mL LPS and compounds.

The NHDF cell line was firstly treated with LPS for 24 h to induce inflammation. Then, the culture was washed and incubated with the test compounds for 24 h at a concentration range of 10–100 µM. All of the tested molecules increased the cell viability compared to the positive control (except **5c** and **6c** in the concentration range of 50–100 µM). This fact can suggest that the investigated derivatives could probably exert a good ability to reduce cell inflammation (Figure 5). For the remaining tested compounds, the increase in the activity of mitochondria was statistically significantly higher than in the positive control. At the same time, compounds **6a** and **9a** showed a greater increase in mitochondrial activity than the negative control in the entire range of the tested concentrations.

**Figure 6:**
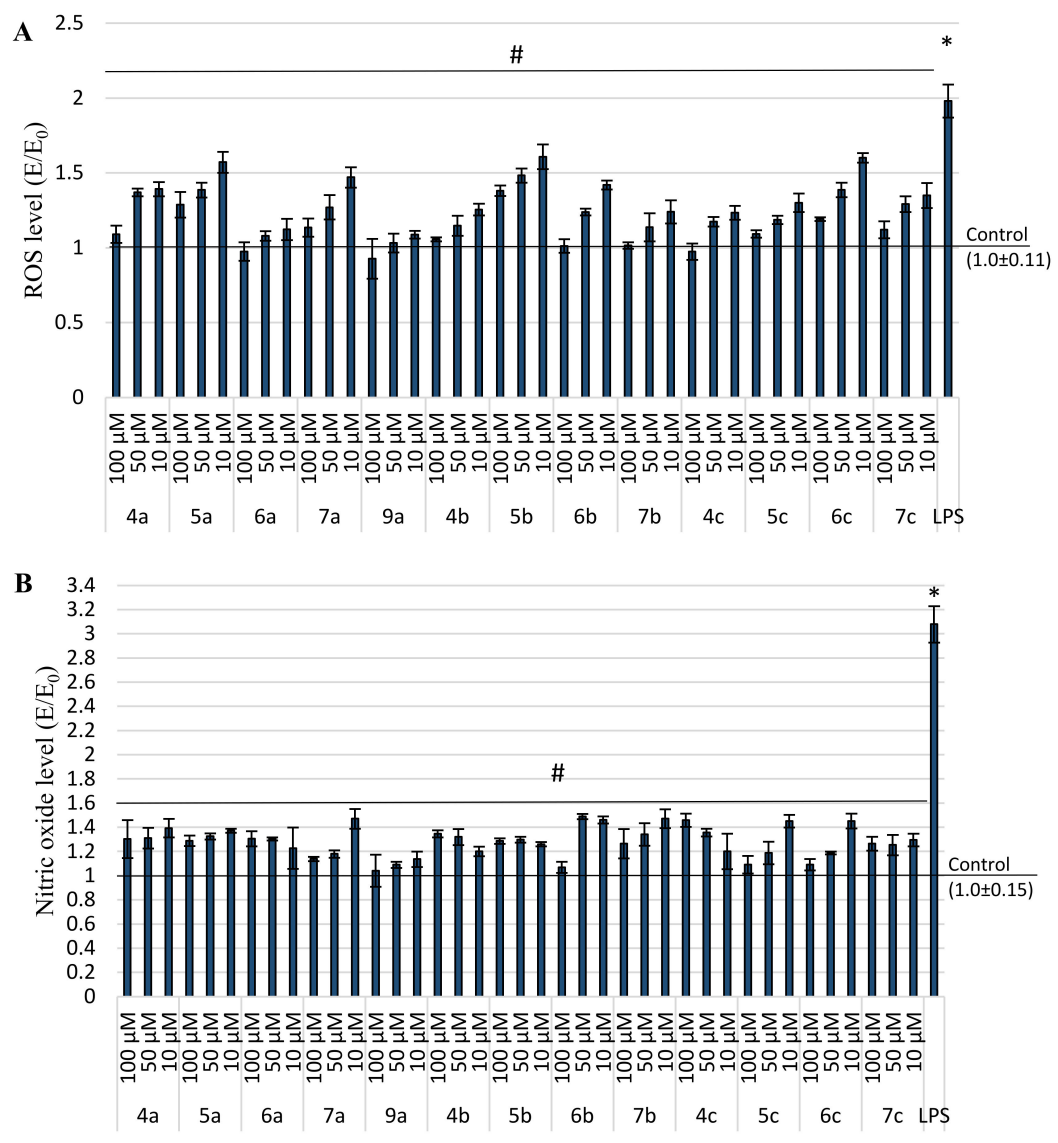
Impact of investigated compounds on NHDF cells after incubation with 50 µg/mL LPS; (**A**) DCF-DA assay and (**B**) Griess assay; # *p* < 0.05—significant difference compared to the control with 50 μg/mL LPS and without compounds; * *p* < 0.05—significant difference compared to a negative control without 50 μg/mL LPS and compounds.

Needless to say, such factors like hypoxia or inflammation can increase the intracellular level of reactive oxygen and nitrogen species (RONS). As a consequence, oxidative and/or nitrosative stress can develop in inflamed tissue [39]. According to many studies, the aforementioned processes often co-exist and potentiate one another [39,40]. Being aware that there is an increased level of free oxygen radicals and nitric oxide (NO) in inflammation, we evaluated whether the tested compounds exhibit antiradical activity in the dichlorofluorescin diacetate (DCF-DA) and Griess assays and assessed the levels of reactive oxygen species (ROS) and NO, respectively (Figure 6A,B).

The dependence on the concentration of the new derivatives of pyrrolo[3,4-*d*]pyridazinone and their antioxidant activity has been demonstrated in the executed experiments (Figure 6). The higher the concentration of the tested compound, the stronger the scavenging of oxygen free radicals has been noticed. All of the tested compounds statistically significantly decreased the level of ROS as compared to the positive control. Moreover, the derivatives **6a** and **9a** scavenged oxygen free radicals to the level of the negative control in the whole range of the tested concentrations. At the same time, all of the examined molecules showed a statistically significant reduction in the NO level, as compared to the positive control. Compound **9a** was the only one that lowered NO levels to negative control levels over the whole range of the investigated concentrations.

### 2.5. Structure-Activity Relationship Study

**Figure 7:**
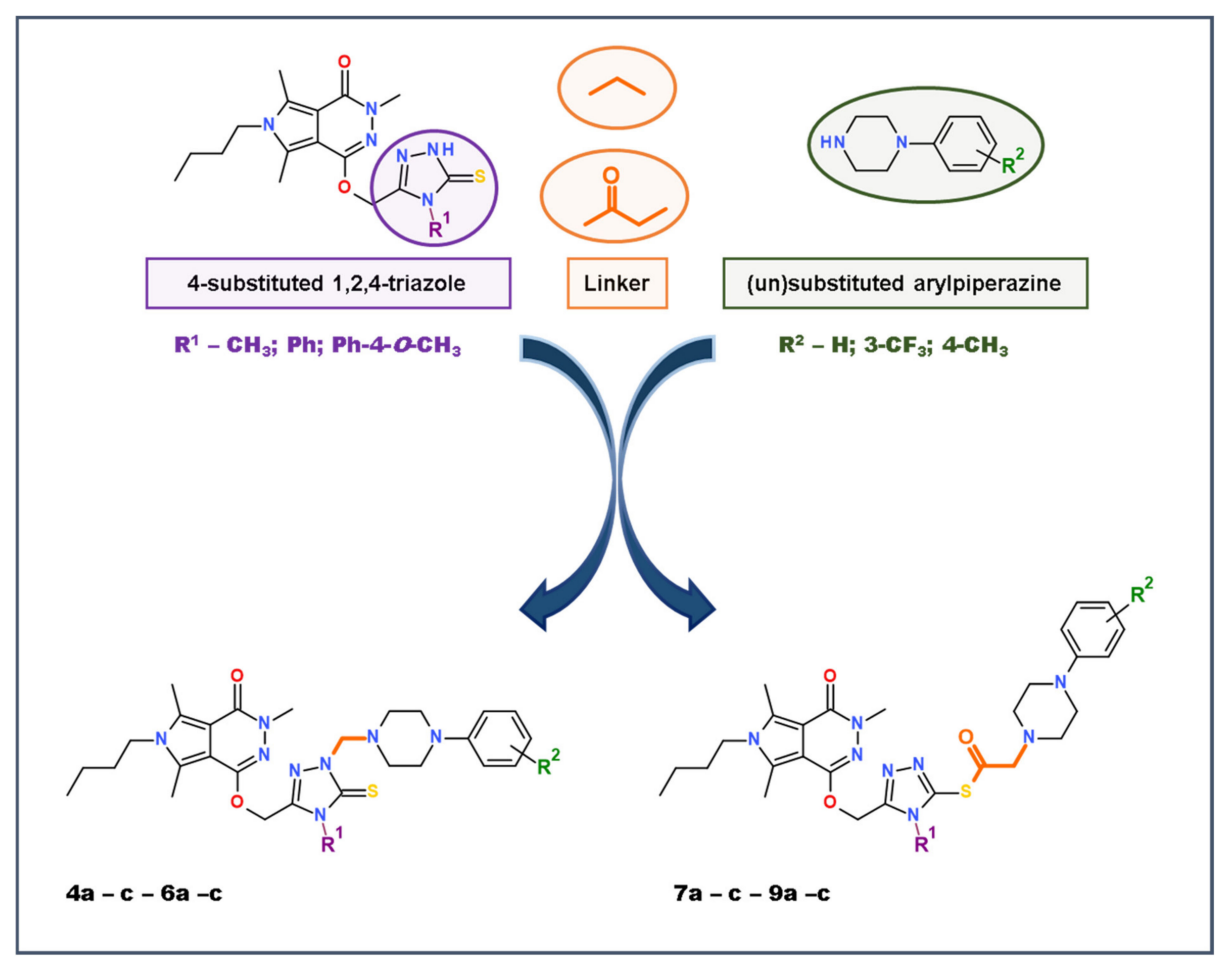
The concept of design and synthesis of target *N*-substituted-1,2,4-triazole-based derivatives of pyrrolo[3,4-*d*]pyridazinone.

When considering the composition of 18 title compounds reported in this paper, we can point out three characteristic and variable structural elements which could have the impact on the toxicity and the biological activity of the investigated derivatives (Figure 7). The first one is the substituent in position 4 in the 1,2,4-triazole ring. We have introduced there residues of a different size and character. Based on this structural element, we have divided title compounds into three main series: (**a**) with methyl group, (**b**) with phenyl ring and (**c**) with 4-methoxyphenyl moiety. The second one is the aryl piperazine pharmacophore—being more precise, the substituent or its lack in its phenyl ring. In this case, we can also demonstrate three groups of compounds. The first one is those with unsubstituted phenylpiperazine moiety (**4a-c**, **7a-c**). In the second class (**5a-c**, **8a-c**) we can distinguish the derivatives with the trifluoromethyl group in position 3. On the other hand, compounds **6a-c**, **9a-c** possess a substituent completely different in the size and electronic character—a methyl residue in position 4 of phenyl ring. Finally, when taking into consideration the way in which the aryl piperazine pharmacophore is linked with 1,2,4-triazole moiety, we can divide the target compounds into two groups. Mannich base-type derivatives are the first one (**4a-c-6a-c**), while the second one consists of structures with flexible oxoethylene linker (**7a-c-9a-c**) received via *S*-alkylation of 1,2,4-triazole.

When analyzing the results of the cell viability evaluation, we can see that there is a significant relationship between the structure and cytotoxicity of the titled compounds. First of all, in every series, **a**, **b** and **c** molecules with unsubstituted phenylpiperazine, that is **4** and **7**, appeared to be non-toxic. On the contrary, all of the derivatives with both the trifluoromethyl group and oxoethylene linker (**8a-c**) caused cell lysis. Such an effect was also observed in the case of compounds possessing 4-methylphenylpiperazine pharmacophore connected via the oxoethylene group with 1,2,4-triazole, which are **9b** and **9c**. Continuing our considerations, it is worth noticing that the data shown in the Table 1 clearly indicate that out of six compounds from series **a** (methyl residue in 1,2,4-triazole), as many as five derivatives (**4a-7a**, **9a**) did not reveal a cytotoxic effect at all. Therefore, the presence of a small substituent in that place appear to be the most favorable. Moreover, Mannich base derivatives (**4a-c-6a-c**) were found to be less toxic than compounds with oxoethylene linker (**7a-c-9a-c**).

We can assume that the introduction of a large substituent into position 4 of 1,2,4-triazole ring, with the simultaneous presence of substituted aryl piperazine pharmacophore, leads to an increase of cytotoxicity, which may be caused by large molecular weight. In this case, the nature and size of the residue in the triazole ring seems to be substantial. The compounds with a small methyl group in the position 4 of 1,2,4-triazole ring and/or unsubstituted phenylpiperazine pharmacophore did not affect the cell viability at all (except **8a**). Summing up, the cytotoxic effect grew up alongside with increased molecular weight and the size of molecule. Derivatives **8a-c**, **9b-c** were excluded from further investigations.

Considering COX inhibition studies, derivatives **5b**, **6b**, **5c-7c**, did not show any activity in in vitro assay and their binding free energy (ΔG°) for interaction with both of the isoenzymes presented positive values. Thus, a large substituent in 1,2,4-triazole ring led to a decrease in the biological activity. From series **b** and **c** only **4b**, **7b** and **4c** revealed potential COX inhibition. Compounds **4a-7a**, **9a** demonstrated good cyclooxygenase inhibitory activity, especially towards COX-2 isoform (Table 2). It is worth to mention, that **5a**, **7a**, **4b** and **7b** inhibited also COX-1 isoform in vitro, but it was not confirmed by the results of the molecular docking studies. Moreover, the IC_50_ values of active compounds determined for both COX-1 and COX-2 were very similar to that defined for *Meloxicam*. Taking into consideration the results obtained in in vitro COX inhibition assay and in the molecular docking studies, the most promising appeared to be derivatives from series **a**.

Analyzing the result of performed in vitro anti-inflammatory and antioxidant activity evaluation within cells, we did not notice such meaningful differences between the investigated compounds. All of the derivatives exerted a good ability to reduce induced inflammation, except **5c** and **6c**. Once more, structures which belong to series **a** turned out to be the most potent.

Taking all of the above into account, we can suppose that the nature of aryl piperazine pharmacophore and the linker had the main impact on the compounds’ toxicity. On the other hand, the substituent in the position 4 of 1,2,4-triazole had the influence on both the cytotoxicity and the COX inhibitory activity.

**Figure 8:**
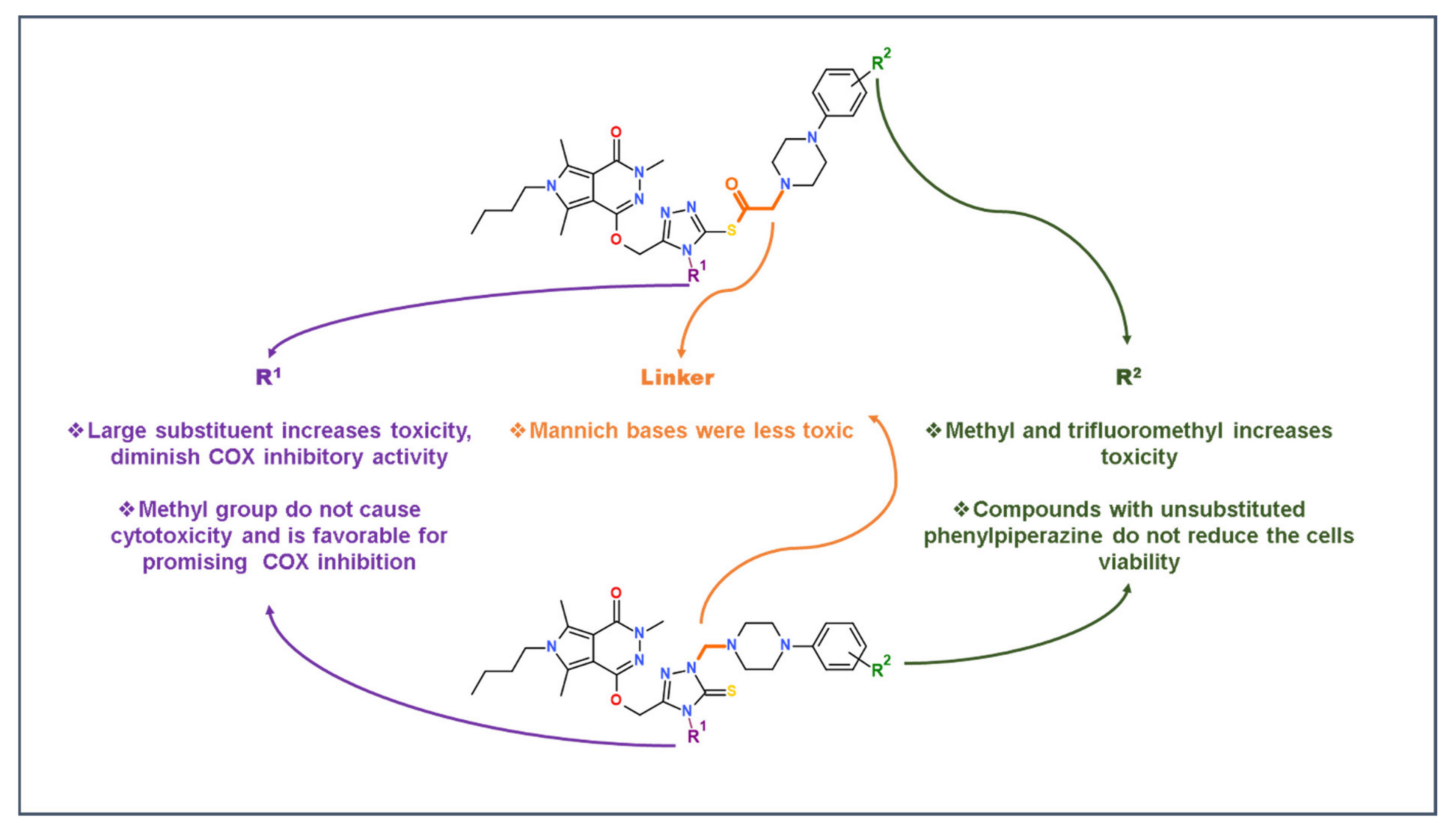
The structure-activity relationships in the group of investigated molecules.

Our consideration concerning the structure-activity relationships in the group of the investigated target molecules are summarized in Figure 8.

### 2.6. Bovine Serum Albumin (BSA) Ligand-Binding Assay

The interaction of new compounds with blood proteins affects their pharmacokinetics in vivo. Therefore, we have performed experiments which were aimed at the estimation of the binding mode of the target compounds with the bovine serum albumin (BSA). Its structure is very similar to human serum albumin (HSA), and therefore can be used instead of human protein, especially since the costs of the application of bovine protein are significantly lower [41,42]. The mature BSA protein consists of 583 amino acids and is formed by 3 homologous domains, I, II and III, which in turn are composed of 2 sub-domains: A and B [42]. Aromatic and heterocyclic ligands can bind to hydrophobic cavities in the subdomains IIA and IIIA [41]. The molecular interaction between new ligands and bovine serum albumin can be monitored by optical techniques such as circular dichroism (CD), FT-IR, ultraviolet–visible (UV-vis) or fluorescence spectroscopy [43].

#### 2.6.1. Fluorescence Quenching of BSA, Binding Constants, Thermodynamic Studies

**Figure 9:**
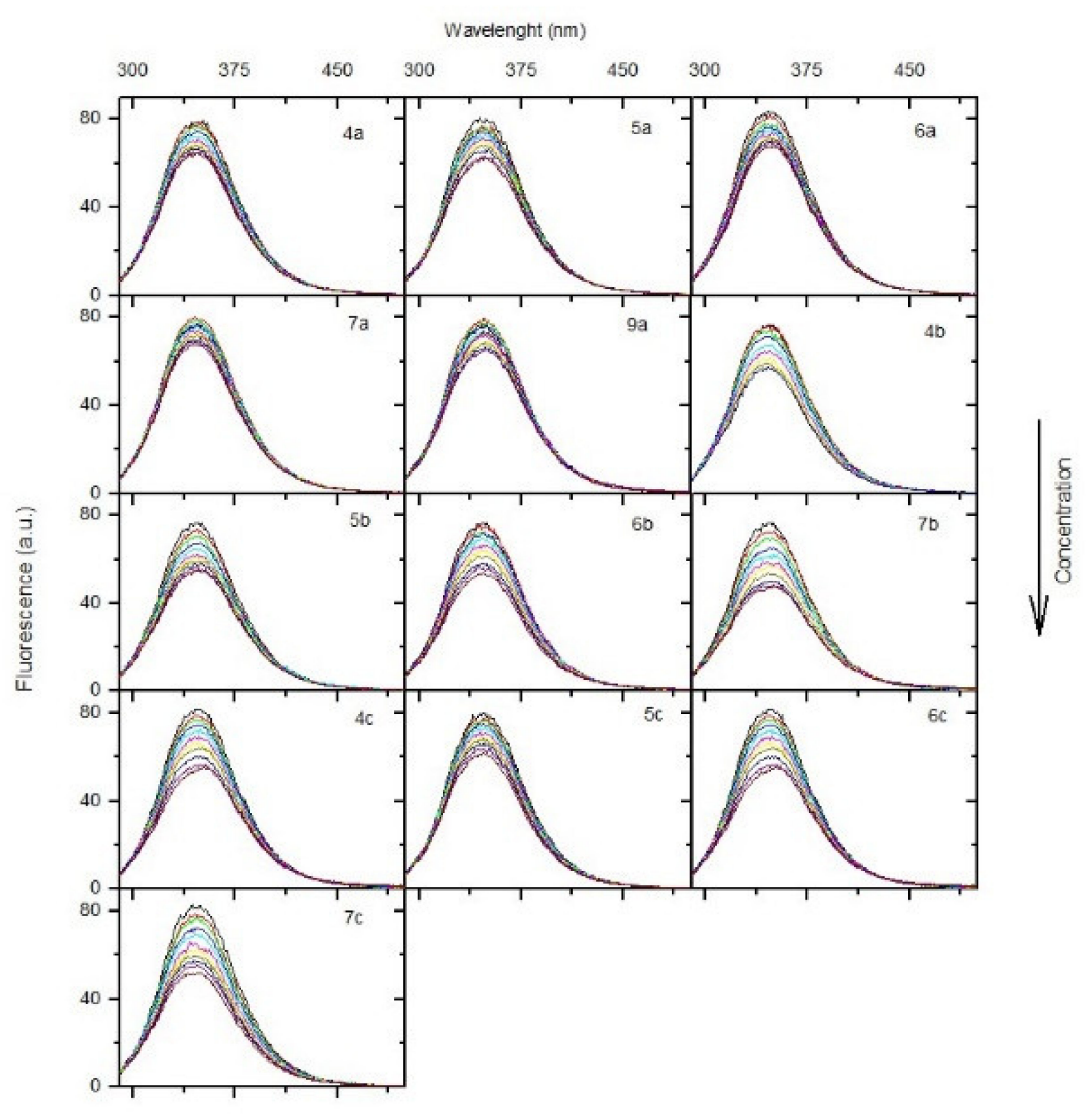
Fluorescence spectra of BSA solution in the presence of studied compounds (T-297 K, λex = 280 nm). The concentration of derivatives from series a, b and c increased gradually as follows: 0, 0.2, 0.4, 0.6, 0.8, 1.0, 1.2, 1.4, 1.6, 1.8, 2.0 µM.

In this study, we checked the binding properties of the analyzed compounds to bovine serum albumin (BSA). Due to this, the fluorescence spectra were recorded in the range of 300–500 nm upon excitation at 280 nm, where both Trp and Tyr residues are excited, and concentration range 0.0–2.0 µM (Figure 9). After the addition of each portion of the analyzed compound, the fluorescence intensity of BSA decreased. It suggests that all compounds could interact with BSA. The analyzed compounds can interact with BSA in two ways: forming a complex (what means static quenching) or due to collisions between molecules (dynamic quenching). The analysis by the Stern–Volmer equation, in dependence on temperature, can explain which way of interaction is observed in the case of our compounds [44].

The Stern–Volmer Equation (1) [45] at three different temperatures 297, 303, 308 K, was used for all of the fluorescence data after correction due to the infer filter effect (2):

(1) $$F_{corr}=F_{obs}10^{\frac{\left(A_{ex}+A_{em}\right)}{2}}$$

where, F_corr_ and F_obs_ are the corrected and observed fluorescence intensities, respectively. A_ex_ and A_em_ are the absorbance values at excitation and emission wavelengths, respectively.

(2) $$\frac{F_{O}}{F}=1+k_{q}\tau \left[Q\right]=1+K_{SV}$$

where F_0_ and F are the steady-state fluorescence intensities at the maximum wavelength in the absence and presence of quencher, respectively, k_q_ the quenching rate constant of the biomolecule, τ_0_ the average lifetime of the biomolecule, [Q] is the quencher concentration and K_sv_ is the Stern–Volmer constant.

The Stern–Volmer (K_SV_) constant was determined by linear fitting. The calculated results are collected in Table 4. When the temperature increases, the K_SV_ values also decrease. Furthermore, the quenching rate constant (k_q_) values are much greater than the value of the maximum scatter collision quenching constant equal to 2 × 10^10^ dm^3^·mol^−1^·s^−1^ [46]. Thus, all of the above indicate that the static quenching mechanism is more probable than dynamic and suggests forming the ground–state complex.

A double logarithm regression curve (3) was used to calculate the binding constants and the number of binding sites:

(3) $$log\frac{F_{0}-F}{F}=logK_{b}+nlog\left[Q\right]$$

where F_0_ and F are the steady-state fluorescence intensities at the maximum wavelength in the absence and presence of quencher, respectively, [Q] is the quencher concentration.

**Figure 10:**
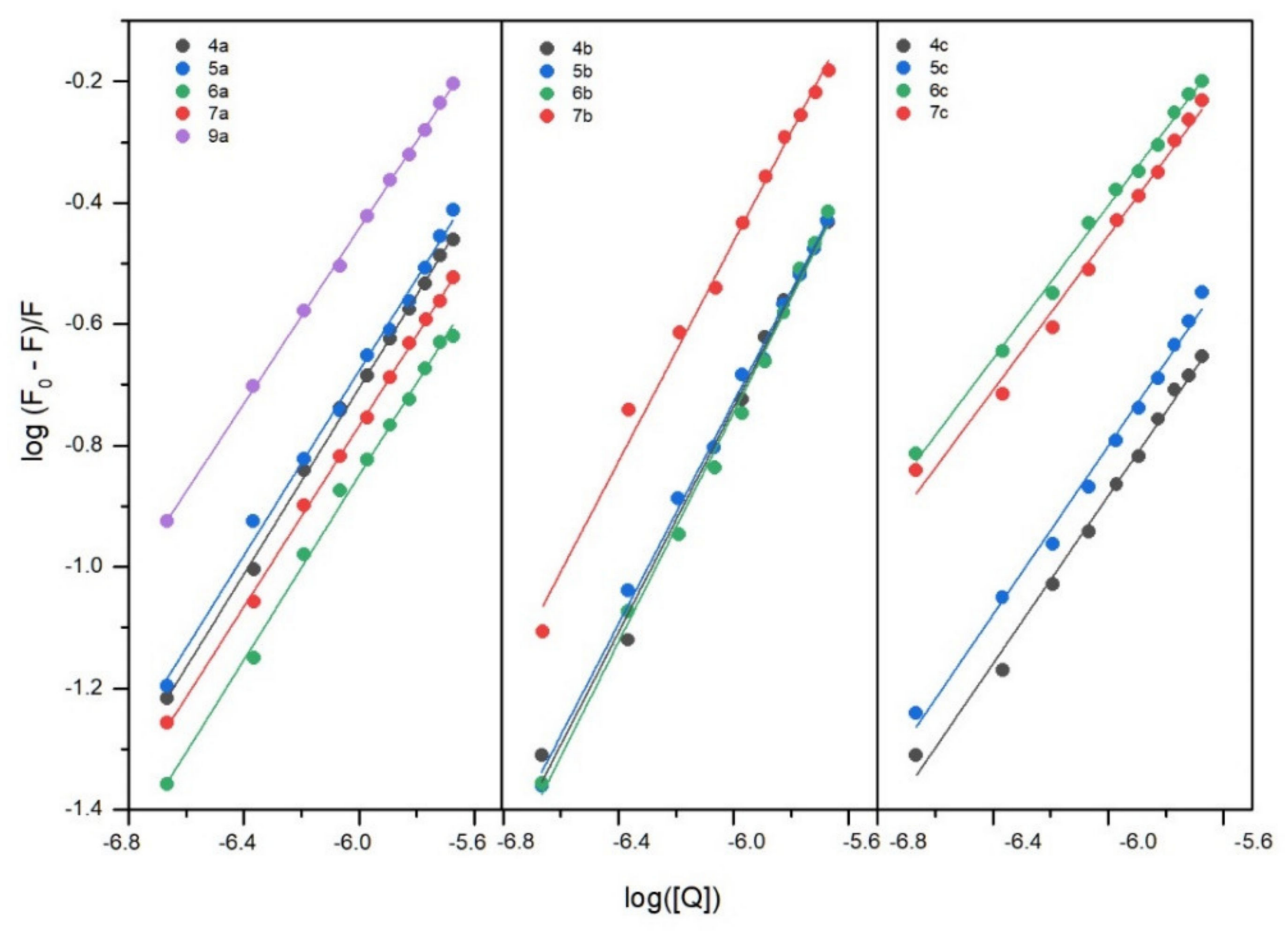
Fluorescence spectra of BSA solution in presence of studied compounds (T-297 K, λex = 280 nm). The concentration of a, b and c was as follows: 0, 0.2, 0.4, 0.6, 0.8, 1.0, 1.2, 1.4, 1.6, 1.8, 2.0 µM.

Figure 10 shows a good linear fit for all of the studied compounds. The results listed in Table 4 made visible that the binding constants indicate values of about 10^5^ dm^3^·mol^−1^ at 297 K and decrease in higher temperatures. Similar values were obtained for many biologically active compounds [44,47,48,49,50,51]. Thus, the number of binding sites is close to 1 for all of the studied compounds, which means a one-to-one interaction.

Small molecules can interact with proteins in various ways, such as a hydrogen bond, van der Waals force, electrostatic and hydrophobic interactions, etc. [52]. The values of the thermodynamic parameters, enthalpy change (ΔH°), the entropic change (ΔS°) and free energy change (ΔG°), indicate the manners of these interactions.

The thermodynamic parameters were calculated from Equations (4) and (5):

(4) $$logK_{b}=-\frac{\Delta H^{{}^{\circ}}}{RT}+\frac{\Delta S^{{}^{\circ}}}{R}$$

(5) $$\Delta G^{{}^{\circ}}=\Delta H^{{}^{\circ}}-T\Delta S^{{}^{\circ}}=-RTlnK_{b}$$

where K_b_ is the binding constant, R is the universal gas constant.

The results are listed in Table 4. The negative ΔG° values indicate that the interaction between BSA and the analyzed compounds was spontaneous. Simultaneously, the ΔH° and ΔS° also had negative values. Due to this, it can be concluded that van der Waals forces and/or hydrogen bonds were the main interaction types in the binding process.

#### 2.6.2. Circular Dichroism Spectra

Circular dichroism spectroscopy is a good method to determine the changes in the secondary structure in the conformation of proteins and to check if the analyzed compounds can interact with protein molecules [53]. In this study, we investigated the changes in the structure of BSA when all of the analyzed compounds were absent or present in solutions. Two negative bands characteristic for BSA, at near 208 nm and 222 nm, were observed in all of the CD spectra (Table S6 in Supplementary Data), which is typical for the α-helical structure of the protein. Any changes in this region mean conformational changes in protein molecules [54]. On the CD spectra shown in Table S6, a reduction of ellipticity values at 208 nm and 222 nm after adding every portion of the analyzed compounds can be seen. The loss in the α-helix(%) was observed. The content of the α-helix was calculated using Equations (6) and (7):

(6) $$\alpha -helix\left(\%\right)=\frac{-MRE_{208}-4000}{33000-4000}100\%$$

where MRE_208_ is the MRE value observed at 208 nm, 4000 and 33,000 is the MRE value of the β-form and random coil conformation cross at 208 nm value of pure α-helix at 208 nm, respectively.

(7) $$MRE=\frac{ObservedCD\left[mdeg\right]}{10Cnl}$$

where C is the molar concentration of BSA, n is the number of amino acid residues, which is 583 for BSA, l is the path length in cm [55].

**Figure 11:**
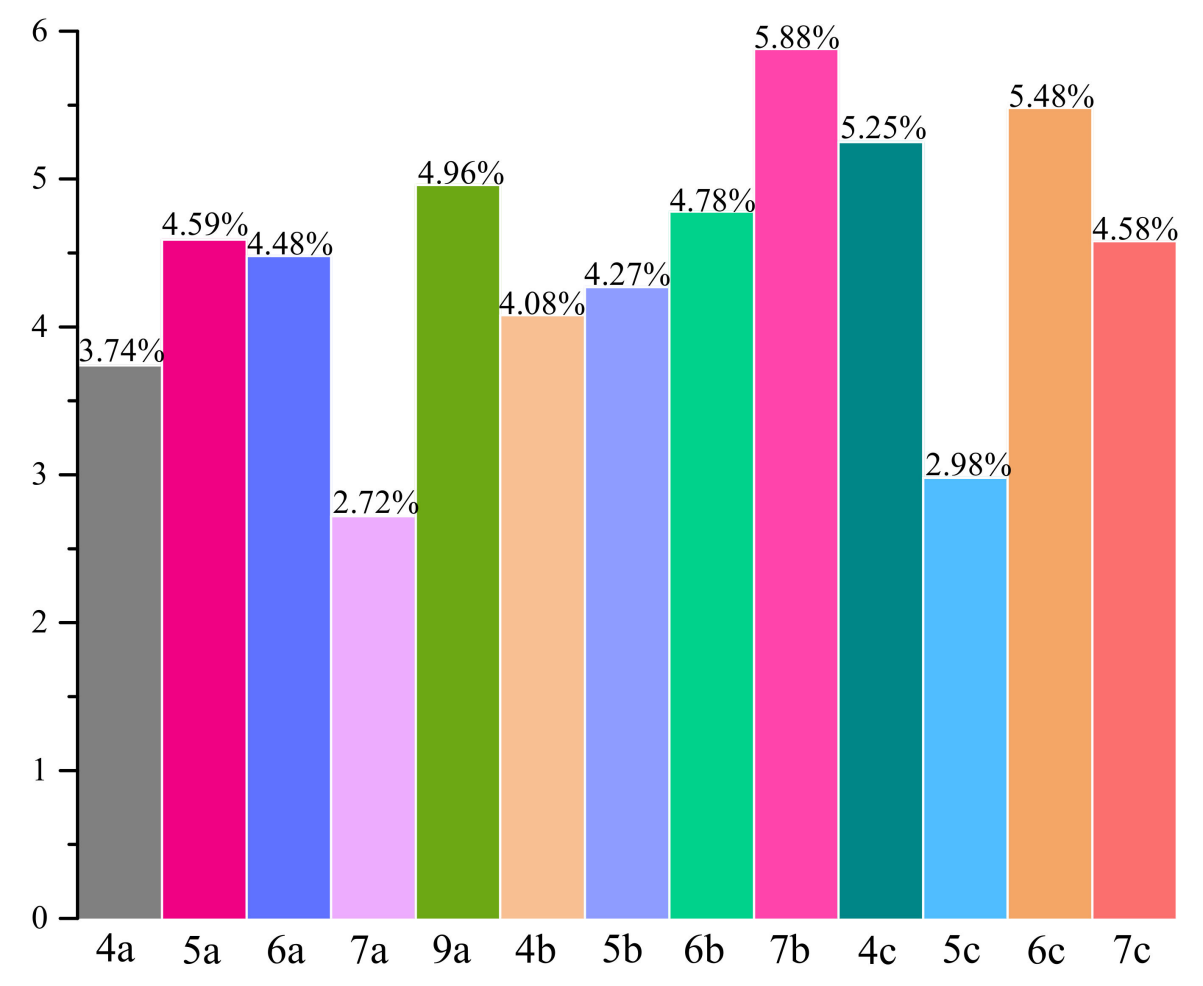
The comparison of changes in the α-helix(%) of BSA after adding 10 portions of each analyzed compound.

The reduction in the α-helical contents of BSA is observed in the presence of all of the analyzed compounds. The changes after adding every portion of analyzed ligands are presented in Table 5. The observed changes ranged from 2.72% for **7a** to 5.88% for **7b** (Figure 11). Thus, CD studies showed that all of the analyzed compounds could interact with BSA, which agrees with the fluorescence spectroscopy.

#### 2.6.3. Fourier Transform Infrared Spectroscopic Measurements

The interaction of BSA with a series of investigated compounds was evaluated by analyzing changes in the protein secondary structure. The backbone conformation of BSA is related to the shape and intensity variation of the Amide I bond (C=O—stretching) and Amide II bond (C–N—stretching coupled with N–H bending) detected in the range 1700–1600 cm^−1^ [56]. Shi et al. mention that the Amide I bond is more sensitive than the Amide II bond for changes in the secondary structure [42]. The major signals found for free BSA: 1658 cm^−1^ and 1550 cm^−1^ are related to the α-helix structure and contribution of 53.8% (Table 6). It corresponds well with the CD measurements. The second large share has the β-sheet structure, which accounts for 29.2% and manifests as 1638 cm^−1^, 1630 cm^−1^ and 1618 cm^−1^ peaks (Table 6, Table S7a). For all of the measurement BSA-compound complexes, it was observed that there was a tendency to decrease the intensity of the 1650 cm^−1^ and 1545 cm^−1^ signals with an increase of the compound concentration (Table S8 in Supplementary Data). This clearly proves that all of the presented compounds interact with BSA, and binding to a protein is demonstrated by changing its secondary structure. A reduction in the α-helix structures in BSA was observed for all of the studied compounds after binding them to the protein. These results are in good agreement with fluorescence and CD spectroscopy. The majority of the changes were exhibited in It is with good correspondence the **5b, 5c, 6b, 6c** and **7c** compounds. Moreover, the certain losing β-sheet structure occurred, especially for the **5c, 6c** and **7c**. These findings correspond to a rise in the percentage of β-turn and β-antiparallel in the secondary structure of BSA. Moreover, the higher contribution of disordered random coil stood out in the samples containing **5c, 6c** and **7c**.

#### 2.6.4. Site Markers Studies and Molecular Docking

In the BSA molecule, there are two binding sites situated in subdomains IIA and IIIA [57]. To confirm the binding sites where the analyzed compounds can be bound, *Phenylbutazone* (PHB) and *Ibuprofen* (IBP) were used as site probes [58]. Equation (3) was used to analyze the results here. The obtained results are collected in Table 7. The values of K_b_ of all of the tested compounds with BSA in the presence of IBP and PHB decline compared to compounds without BSA (Table 7). However, in the case of the values with PHB, the differences are smaller than in the case of IBP. Therefore, the results suggest that all of the studied compounds mainly bind to subdomain IIIA of BSA.

**Figure 12:**
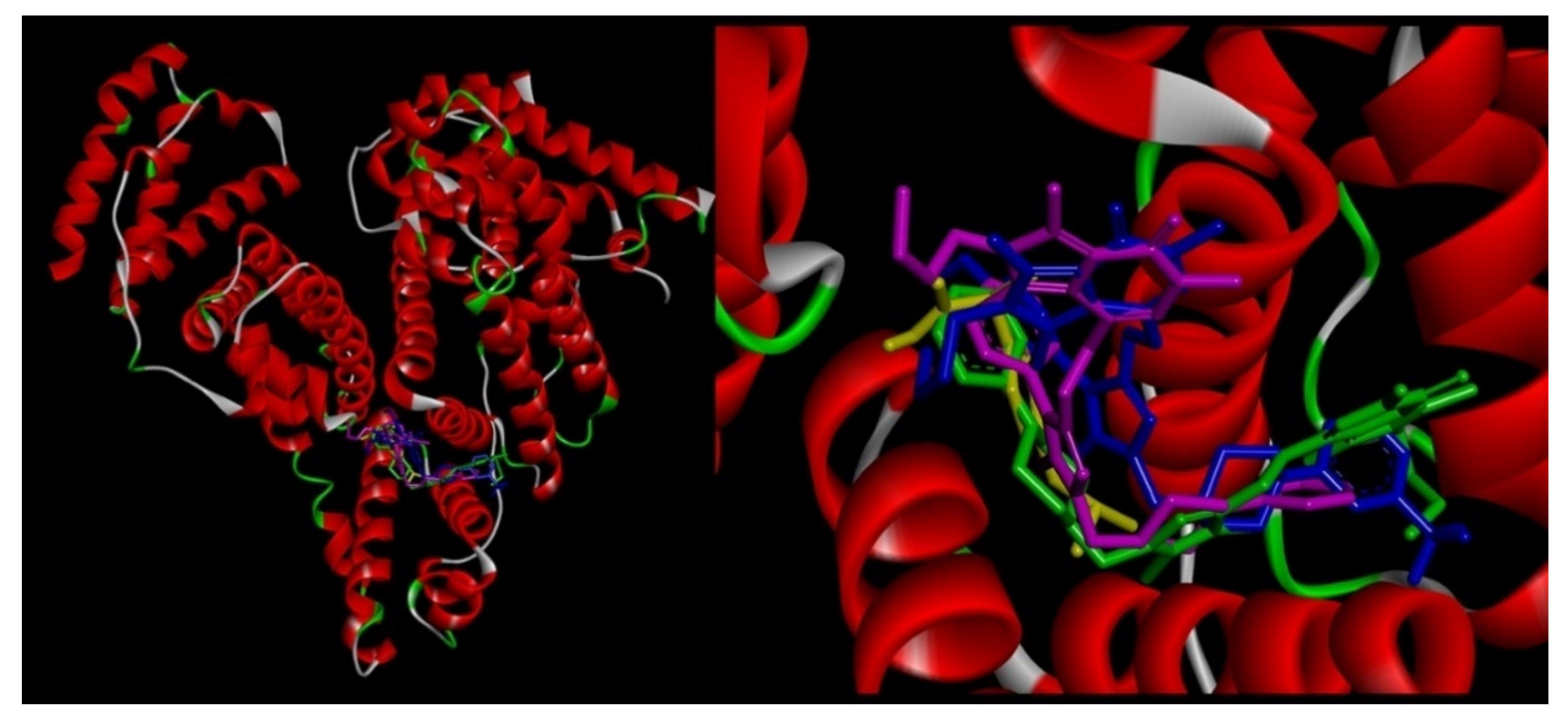
Docking poses of **4a** (pink), **4b** (green), **5c** (blue) and *Ibuprofen* (yellow) inside BSA site II (m).

**Figure 13:**
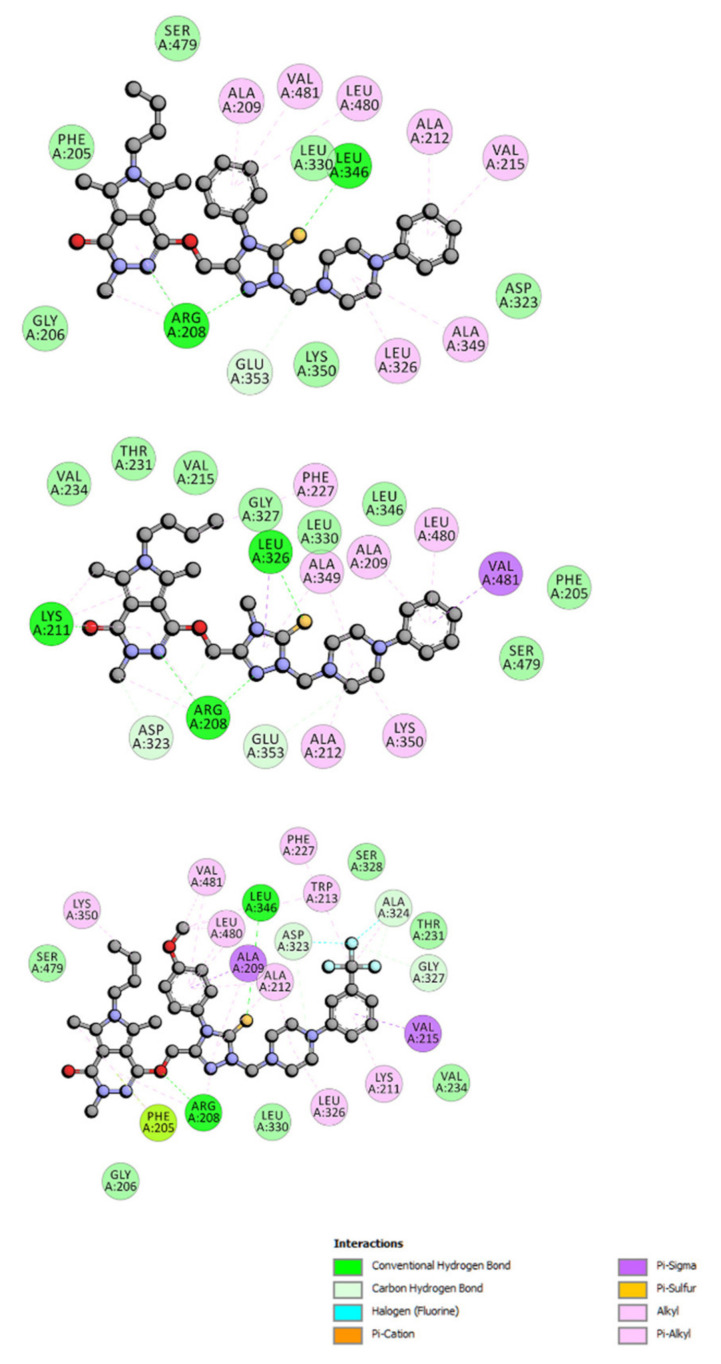
Two-dimensional interaction plot of **4b**, **4a**, and **5c** with BSA in site 2 (m).

The binding interactions between the tested compounds and BSA were studied by the molecular docking method. The simulated results were listed in Table 8. The results revealed that the binding free energy for all of the compounds within the hydrophobic cavity in site II (m) of BSA was more negative than that within the hydrophobic cavity in site I and site II(l). This indicates that site II (m) is favorable. The value of electrostatic energy (ΔE_3_) is much less than ΔE_2_. It can indicate that the main interactions are van der Waals and hydrogen bonding interactions. The position of the compounds with the lowest binding free energy from all of the studied series in site II (m) compared to *Ibuprofen* is shown in Figure 12. The compounds are surrounded by various kinds of residues (Figure 13). Hydrogen bonds with Arg208, Leu326, Leu346 are formed. The π-sigma and other hydrophobic interactions are observed.

### 2.7. In Silico Pharmacokinetic and Druglikeness Prediction

The derivatives **4a-7a**, **9a**, **4b-7b**, **4c-7c** were predicted for their possible pharmacokinetic (absorption distribution metabolism excretion; ADME) and drug-likeness properties using the SWISSADME server (http://www.swissadme.ch/index.php, accessed on 9 September 2021). The simulated results concerning the physicochemical features in the context of the Lipinski’s rule of five (Ro5) [59] are presented in the Table 9. The compounds **4a-6a**, **4b** and **6b** meet the conditions of the Ro5, and therefore are supposed to show good oral bioavailability and membrane permeability. These findings are supported by the results collected in Table 10. Most of the investigated molecules are predicted to be highly absorbed through the gastrointestinal (GI) tract. On the other hand, none of them are expected to cross blood–brain barrier (BBB). The water solubility was found to be rather poor (Table 10).

According to the results presented in Table 11, the derivatives **4a-6a**, **4b**, **6b** not only do not violate the Lipinski’s rule of five, but also fulfill the descriptors of Veber’s rule [59,60]. This factors, alongside with the promising bioavailability score and calculated values of topological polar surface area (TPSA) (<140 Å^2^) [61], can suggest that our compounds could be easily transported through biological membranes.

## 3. Materials and Methods

### 3.1. Chemistry

#### 3.1.1. Instrumentation and Chemicals

All of the chemicals, solvents and reagents used during chemical synthesis, purification and other experiments were delivered by commercially available suppliers (Alchem, Wrocław, Poland; Chemat, Gdańsk, Poland; Archem, Łany, Poland) and were used without further purification. Dry solvents were received according to the standard procedures. The reaction progress was monitored using the thin-layer chromatography (TLC) technique on silica-gel-60-F254-coated TLC plates, which were observed in UV light at 254 or 366 nm. The melting points of all of the new compounds were determined on the Electrothermal Mel-Temp 1101D apparatus (Cole-Parmer, Vernon Hills, IL, USA) using the open capillary method and were uncorrected. The ^1^H NMR (300 MHz) and ^13^C NMR (75 MHz) spectra were recorder on the Bruker 300 MHz NMR spectrometer (Bruker Analytische Messtechnik GmbH, Rheinstetten, Germany). The samples were dissolved in CDCL_3_ or DMSO-*d_6_*, and tetramethylsilane (TMS) was used as an internal reference. Chemical shifts (δ) were reported in ppm. The infrared (IR) spectra were determined on the Nicolet iS50 FT-IR Spectrometer (Thermo Fisher Scientific, Waltham, MA, USA). The samples were applied as solids, and the frequencies were reported in cm^−1^. Mass spectra (MS) were recorded using the Bruker Daltonics Compact ESI-Mass Spectrometer (Bruker Daltonik, GmbH, Bremen, Germany), operating in the positive ion mode. The analyzed compounds were dissolved in a methanol–chloroform mixture. All of the new reported derivatives were determined to have purities of >95% by the above-mentioned methods, unless stated otherwise.

#### 3.1.2. Chemical Synthesis

The synthesis protocols and experimental data for compound **1** and all of the intermediates have already been reported [34].

##### General Procedure for Preparation of *N*-Substituted(aminothioxomethyl)hydrazide Derivatives of Pyrrolo[3,4-*d*]pyridazinone (**2a-c**)

The 2-(6-butyl-3,5,7-trimethyl-4-oxo-pyrrolo[3,4-*d*]pyridazin-1-yl)oxyacetohydrazide **1** (0.001 mol) was suspended in anhydrous ethanol (25 mL), and the mixture was heated under reflux until the hydrazide dissolved completely. Then, the appropriate *N*-substituted isothiocyanate (0.0011 mol) was added, and the reflux was continued for a further 0.5–2 h till the product precipitated. Finally, the mixture was cooled down, and the solid was filtered off, thoroughly washed with ethanol and purified by crystallization from this solvent.

**2a:**1-[[2-(6-butyl-3,5,7-trimethyl-4-oxo-pyrrolo[3,4-*d*]pyridazin-1-yl)oxyacetyl]amino]-3-methyl-thiourea

Yield: 87.56%; m.p.: 207–209 °C;

FT-IR (selected lines, γ_max_, cm^−1^): 3263 (N-H), 2960, 2937, 2875 (C-H aliph.), 1698 (C=O), 1372 (C=S), 1231 (C-O), ^1^H NMR (300 MHz, DMSO-*d_6_*) δ: 0.88–0.93 (m, 3H, -CH_2_-CH_2_ CH_2_-CH_3_), 1.27–1.35 (m, 2H, -CH_2_-CH_2_ CH_2_-CH_3_), 1.57 (m, 2H, -CH_2_-CH_2_ CH_2_-CH_3_), 2.51 (s, 3H, 7-CH_3_), 2.58 (s, 3H, 5-CH_3_), 2.85 (s, 3H, *N*-CH_3_), 3.38 (s, 3H, 3-CH_3_), 3.94–3.99 (m, 2H, -CH_2_-CH_2_ CH_2_-CH_3_), 4.70 (s, 2H, *O*-CH_2_-); 7.88 (s, 1H, *N*H), 9.31 (s, 1H, *N*H), 9.96 (s, 1H, *N*H); ^13^C NMR (75 MHz, DMSO-*d_6_*) δ: 10.65, 11.33, 13.99, 19.91, 31.27, 32.06, 37.04, 63.74, 108.00, 111.23, 123.58, 129.07, 148.57, 158.46, 167.62, 182.59; MS (ESI-MS) (*m*/*z*): calcd. for C_17_H_26_N_6_O_3_S [L+H]^+^: 395.1860; found: 395.1839.

**2b:**1-[[2-(6-butyl-3,5,7-trimethyl-4-oxo-pyrrolo[3,4-*d*]pyridazin-1-yl)oxyacetyl]amino]-3-phenyl-thiourea

Yield: 89.88%; m.p.: 214–216 °C;

FT-IR (selected lines, γ_max_, cm^−1^): 3323, 3237 (N-H), 3049 (C-H arom.), 2961, 2938, 2876 (C-H aliph.), 1689 (C=O), 1360 (C=S); ^1^H NMR (300 MHz, DMSO-*d_6_*) δ: 0.88–0.93 (m, 3H, -CH_2_-CH_2_ CH_2_-CH_3_), 1.28–1.35 (m, 2H, -CH_2_-CH_2_ CH_2_-CH_3_), 1.58 (m, 2H, -CH_2_-CH_2_ CH_2_-CH_3_), 2.53 (s, 3H, 7-CH_3_), 2.58 (s, 3H, 5-CH_3_), 3.36 (s, 3H, 3-CH_3_), 3.94–3.99 (m, 2H, -CH_2_-CH_2_ CH_2_-CH_3_), 4.77 (s, 2H, *O*-CH_2_-); 7.16–7.18 (m, 1H, Ar-H), 7.30–7.42 (m, 4H, Ar-H), 9.56 (s, 1H, *N*H), 9.71 (s, 1H, *N*H), 10.18 (s, 1H, *N*H); ^13^C NMR (75 MHz, DMSO-*d_6_*) δ: 10.65, 11.36, 13.99, 19.91, 32.06, 37.03, 63.84, 108.01, 111.25, 123.57, 125.64, 128.56, 129.09, 139.50, 148.57, 158.46; MS (ESI-MS) (*m*/*z*): calcd. for C_22_H_28_N_6_O_3_S [L+H]^+^: 457.2016; found: 457.1985.

**2c:**1-[[2-(6-butyl-3,5,7-trimethyl-4-oxo-pyrrolo[3,4-*d*]pyridazin-1-yl)oxyacetyl]amino]-3-[(4-methoxy)phenyl]-thiourea

Yield: 94.12%; m.p.: 217–219 °C;

FT-IR (selected lines, γ_max_, cm^−1^): 3321, 3219 (N-H), 3051 (C-H arom.), 2957, 2872, 2836 (C-H aliph.), 1678 (C=O), 1348 (C=S); ^1^H NMR (300 MHz, DMSO-*d_6_*) δ: 0.88–0.93 (m, 3H, -CH_2_-CH_2_ CH_2_-CH_3_), 1.28–1.35 (m, 2H, -CH_2_-CH_2_ CH_2_-CH_3_), 1.58 (m, 2H, -CH_2_-CH_2_ CH_2_-CH_3_), 2.53 (s, 3H, 7-CH_3_), 2.58 (s, 3H, 5-CH_3_), 3.36 (s, 3H, 3-CH_3_), 3.73 (s, 3H, *O*-CH_3_), 3.94–3.97 (m, 2H, -CH_2_-CH_2_ CH_2_-CH_3_), 4.76 (s, 2H, *O*-CH_2_-); 6.87–6.90 (m, 2H, Ar-H), 7.22–7.25 (m, 2H, Ar-H), 9.44 (s, 1H, *N*H), 9.61 (s, 1H, *N*H), 10.14 (s, 1H, *N*H); ^13^C NMR (75 MHz, DMSO-*d_6_*) δ: 10.65, 11.35, 13.99, 19.91, 32.06, 37.03, 55.67, 63.84, 108.02, 111.25, 113.78 123.57, 127.77, 129.08, 132.31, 148.59, 157.32, 158.46, 167.69; MS (ESI-MS) (*m*/*z*): calcd. for C_23_H_30_N_6_O_4_S [L+H]^+^: 487.2122; found: 487.2087.

##### General Procedure for Preparation of 4-Substituted-2*H*-1,2,4-triazole Derivatives of Pyrrolo[3,4-*d*]pyridazinone (**3a-c**)

The appropriate *N*-substituted-(aminothioxomethyl)hydrazide derivative of pyrrolo[3,4-*d*]pyridazinone (**2a-c**) (0.0001 mol) was dissolved in a 5% aqueous solution of sodium hydroxide (25 mL), and the mixture was stirred and refluxed for about 2–3 h. Afterward, it was poured onto crushed ice and carefully acidified to pH 2–3 with 7.5% hydrochloric acid (aq), resulting in the formation of a white or yellowish solid of adequate 4-substituted-1,2,4-triazole derivative (**3a-c**). Finally, the afforded precipitate was filtered off, washed with ice-cold water and recrystallized from ethanol.

**3a:**6-butyl-3,5,7-trimethyl-1-[(4-methyl-3-thioxo-2*H*-1,2,4-triazol-5-yl)methoxy]pyrrolo[3,4-*d*]pyridazin-4-one

Yield: 83.12%; m.p.: 117–120 °C;

FT-IR (selected lines, γ_max_, cm^−1^): 3425 (N-H), 3120, 3050 (C-H arom.), 2935, 2871(C-H aliph.), 1544 (C=N), 1340 (C=S); ^1^H NMR (300 MHz, DMSO-*d_6_*) δ: 0.86–0.91 (m, 3H, -CH_2_-CH_2_ CH_2_-CH_3_), 1.26–1.33 (m, 2H, -CH_2_-CH_2_ CH_2_-CH_3_), 1.56 (m, 2H, -CH_2_-CH_2_ CH_2_-CH_3_), 2.42 (s, 3H, 7-CH_3_), 2.57 (s, 3H, 5-CH_3_), 3.40 (s, 3H, 3-CH_3_), 3.50 (s, 3H, triazole-*N*-CH_3_), 3.92–3.97 (m, 2H, -CH_2_-CH_2_ CH_2_-CH_3_), 5.30 (s, 2H, *O*-CH_2_-), 13.82 (s, 1H, *N*H); ^13^C NMR (75 MHz, DMSO-*d_6_*) δ: 10.64, 11.18, 13.97, 19.89, 30.65, 32.01, 37.03, 43.83, 58.09, 107.62, 111.18, 123.42, 129.38, 148.07, 148.89, 158.40, 168.05; HR-MS (ESI-MS) (*m*/*z*): calcd. for C_17_H_24_N_6_O_2_S [L+H]^+^: 377.1754; found: 377.1736.

**3b:**6-butyl-3,5,7-trimethyl-1-[(4-phenyl-3-thioxo-2*H*-1,2,4-triazol-5-yl)methoxy]pyrrolo[3,4-*d*]pyridazin-4-one

Yield: 81.69%; m.p.: 123–125 °C;

FT-IR (selected lines, γ_max_, cm^−1^): 3414 (N-H), 3037 (C-H arom.), 2957, 2928, 2871 (C-H aliph.), 1543 (C=N); ^1^H NMR (300 MHz, DMSO-*d_6_*) δ: 0.86–0.91 (m, 3H, -CH_2_-CH_2_ CH_2_-CH_3_), 1.24–1.31 (m, 2H, -CH_2_-CH_2_ CH_2_-CH_3_), 1.53 (m, 2H, -CH_2_-CH_2_ CH_2_-CH_3_), 2.25 (s, 3H, 7-CH_3_), 2.53 (s, 3H, 5-CH_3_), 3.32 (s, 3H, 3-CH_3_), 3.88–3.93 (m, 2H, -CH_2_-CH_2_ CH_2_-CH_3_), 5.12 (s, 2H, *O*-CH_2_-); 7.45 (m, 5H, Ar-H), 14.04 (s, 1H, *N*H); ^13^C NMR (75 MHz, DMSO-*d_6_*) δ:10.59, 11.20, 13.97, 19.86, 31.97, 36.95, 43.76, 57.85, 107.38, 111.05, 123.31, 128.28, 129.19, 129.69, 129.91, 133.86, 147.76, 148.60, 158.28, 168.90; MS (ESI-MS) (*m*/*z*): calcd. for C_22_H_26_N_6_O_2_S [L+H]^+^: 439.1911; found: 439.1874.

**3c:**6-butyl-3,5,7-trimethyl-1-[[4-(4-methoxy)phenyl-3-thioxo-2*H*-1,2,4-triazol-5-yl]methoxy]pyrrolo[3,4-*d*]pyridazin-4-one

Yield: 84.49%; m.p.: 129–131 °C;

FT-IR (selected lines, γ_max_, cm^−1^): 3414 (N-H), 3041 (C-H arom.), 2956, 2931, 2872 (C-H aliph.), 1543 (C=N);^1^H NMR (300 MHz, DMSO-*d_6_*) δ: 0.87–0.92 (m, 3H, -CH_2_-CH_2_ CH_2_-CH_3_), 1.31 (m, 2H, -CH_2_-CH_2_ CH_2_-CH_3_), 1.54 (m, 2H, -CH_2_-CH_2_ CH_2_-CH_3_), 2.29 (s, 3H, 7-CH_3_), 2.54 (s, 3H, 5-CH_3_), 3.31 (s, 3H, 3-CH_3_), 3.74 (s, 3H, Ar-*O*-CH_3_) 3.92 (m, 2H, -CH_2_-CH_2_ CH_2_-CH_3_), 5.12 (s, 2H, *O*-CH_2_-); 6.96–6.99 (m, 2H, Ar-H), 7.31–7.34 (m, 2H, Ar-H), 13.99 (s, 1H, *N*H); ^13^C NMR (75 MHz, DMSO-*d_6_*) δ:10.59, 11.20, 13.98, 19.88, 31.99, 36.95, 43.78, 55.84, 57.85, 107.46, 111.06, 114.79, 115.18, 123.34, 126.32, 129.19, 129.54, 129.97, 147.83, 148.88, 158.29, 160.13, 169.12; MS (ESI-MS) (*m*/*z*): calcd. for C_13_H_28_N_6_O_3_S [L+H]^+^: 469.2016; found: 469.1976.

##### General Procedure for Preparation of Mannich Base-Type Derivatives of Pyrrolo[3,4-*d*]pyridazinone (**4a-c-6a-c**)

Aqueous formaldehyde of 37% (0.01 mol, ~1mL) was added to the solution of adequate 4-substituted-1,2,4-triazole derivative of pyrrolo[3,4-*d*]pyridazinone (**3a**, **3b** or **3c**) (0.001 mol) in methanol (30 mL). The mixture was stirred at room temperature (RT) for 30 min. Subsequently, a corresponding aryl piperazine derivative (0.0015 mol) was added, and the stirring was continued for a further several hours at RT. The mixture was left overnight. The formed precipitate was filtered off, thoroughly washed with cold methanol and purified by crystallization from methanol.

**4a:**6-butyl-3,5,7-trimethyl-1-[[4-methyl-2-[(4-phenylpiperazin-1-yl)methyl]-3-thioxo-2*H*-1,2,4-triazol-5-yl]methoxy]pyrrolo[3,4-*d*]pyridazin-4-one

Yield: 72.35%; m.p.: 186–188 °C;

FT-IR (selected lines, γ_max_, cm^−1^): 3019 (C-H arom.), 2962, 2937, 2874 (C-H aliph.), 1549 (C=N) 1272 (C=S); ^1^H NMR (300 MHz, CDCl_3_) δ: 0.94–0.99 (m, 3H, -CH_2_-CH_2_ CH_2_-CH_3_), 1.34–1.41 (m, 2H, -CH_2_-CH_2_ CH_2_-CH_3_), 1.60–1.66 (m, 2H, -CH_2_-CH_2_ CH_2_-CH_3_), 2.43 (s, 3H, 7-CH_3_), 2.68 (s, 3H, 5-CH_3_), 2.98–2.99 (m, 4H, CH_2_—piperazine), 3.18–3.19 (m, 4H, CH_2_—piperazine), 3.57 (s, 3H, 3-CH_3_), 3.67 (s, 3H, triazole-*N*-CH_3_), 3.94–3.97 (m, 2H, -CH_2_-CH_2_ CH_2_-CH_3_), 5.21 (s, 2H, *N*-CH_2_-*N*) 5.31 (s, 2H, *O*-CH_2_-); 6.82–6.91 (m, 3H, Ar-H), 7.22–7.27 (m, 2H, Ar-H); ^13^C NMR (75 MHz, CDCl_3_) δ: 10.65, 11.30, 13.69, 20.04, 31.69, 32.32, 37.05, 43.99, 49.29, 50.42, 57.54, 69.49, 108.05, 111.84, 116.32, 119.89, 122.24, 129.10, 129.43, 146.76, 147.95, 151.28, 159.17, 169.74; HRMS (ESI-MS) (*m*/*z*): calcd. for C_28_H_38_N_8_O_2_S [L+H]^+^: 551.2911; found: 551.2894.

**5a:**6-butyl-3,5,7-trimethyl-1-[[4-methyl-2-[[4-[3-(trifluoromethyl)phenyl]piperazin-1-yl]methyl]-3-thioxo-2*H*-1,2,4-triazol-5-yl]methoxy]pyrrolo[3,4-*d*]pyridazin-4-one

Yield: 64.84%; m.p.: 198–200 °C;

FT-IR (selected lines, γ_max_, cm^−1^): 2963, 2935, 2876, 3843 (C-H aliph.), 1650 (C=N), 1242 (C=S); ^1^H NMR (300 MHz, CDCl_3_) δ: 0.94–0.98 (m, 3H, -CH_2_-CH_2_ CH_2_-CH_3_), 1.34–1.41 (m, 2H, -CH_2_-CH_2_ CH_2_-CH_3_), 1.60–1.63 (m, 2H, -CH_2_-CH_2_ CH_2_-CH_3_), 2.42 (s, 3H, 7-CH_3_), 2.68 (s, 3H, 5-CH_3_), 2.96–2.99 (m, 4H, CH_2_—piperazine), 3.22–3.25 (m, 4H, CH_2_—piperazine), 3.57 (s, 3H, 3-CH_3_), 3.67 (s, 3H, triazole-*N*-CH_3_), 3.87–3.92 (m, 2H, -CH_2_-CH_2_ CH_2_-CH_3_), 5.21 (s, 2H, *N*-CH_2_-*N*) 5.31 (s, 2H, *O*-CH_2_-); 7.01–7.07 (m, 3H, Ar-H), 7.30–7.35 (m, 1H, Ar-H); ^13^C NMR (75 MHz, CDCl_3_) δ: 10.65, 11.27, 13.67, 20.04, 31.71, 32.32, 37.04, 43.99, 48.77, 50.23, 57.51, 69.39, 108.05, 111.83, 112.39, 115.99, 118.93, 122.21, 129.45, 129.56, 131.22, 131.64, 146.84, 147.91, 151.32, 159.16, 169.78; MS (ESI-MS) (*m*/*z*): calcd. for C_29_H_37_F_3_N_8_O_2_S [L+H]^+^: 619.2785; found: 619.2748.

**6a:**6-butyl-3,5,7-trimethyl-1-[[4-methyl-2-[[4-[(4-methyl)phenyl]piperazin-1-yl]methyl]-3-thioxo-2*H*-1,2,4-triazol-5-yl]methoxy]pyrrolo[3,4-*d*]pyridazin-4-one

Yield: 68.98%; m.p.: 181–183 °C;

FT-IR (selected lines, γ_max_, cm^−1^): 2962, 2933, 2878, 2855, 2826 (C-H aliph.), 1633 (C=N), 1248 (C=S); ^1^H NMR (300 MHz, CDCl_3_) δ: 0.94–0.98 (m, 3H, -CH_2_-CH_2_ CH_2_-CH_3_), 1.34–1.42 (m, 2H, -CH_2_-CH_2_ CH_2_-CH_3_), 1.63 (m, 2H, -CH_2_-CH_2_ CH_2_-CH_3_), 2.26 (s, 3H, Ar-CH_3_), 2.43 (s, 3H, 7-CH_3_), 2.67 (s, 3H, 5-CH_3_), 2.99 (m, 4H, CH_2_—piperazine), 3.15 (m, 4H, CH_2_—piperazine), 3.57 (s, 3H, 3-CH_3_), 3.67 (s, 3H, triazole-*N*-CH_3_), 3.87–3.92 (m, 2H, -CH_2_-CH_2_ CH_2_-CH_3_), 5.21 (s, 2H, *N*-CH_2_-*N*) 5.31 (s, 2H, *O*-CH_2_-); 6.85 (m, 2H, Ar-H), 7.05–7.08 (m, 2H, Ar-H); ^13^C NMR (75 MHz, CDCl_3_) δ: 10.66, 11.33, 13.70, 20.05, 20.44 31.70, 32.33, 37.06, 43.99, 50.34, 57.54, 69.43, 108.04, 111.82, 112.26, 116.81, 122.26, 129.41, 129.67, 146.77, 147.95, 159.18, 169.72; MS (ESI-MS) (*m*/*z*): calcd. for C_29_H_40_N_8_O_2_S [L+H]^+^: 565.3068; found: 565.3019.

**4b:**6-butyl-3,5,7-trimethyl-1-[[4-phenyl-2-[(4-phenylpiperazin-1-yl)methyl]-3-thioxo-2*H*-1,2,4-triazol-5-yl]methoxy]pyrrolo[3,4-*d*]pyridazin-4-one

Yield: 69.02%; m.p.: 129–131 °C;

FT-IR (selected lines, γ_max_, cm^−1^): 2932, 2828 (C-H aliph.), 1543 (C=N), 1269 (C=S); ^1^H NMR (300 MHz, CDCl_3_) δ: 0.94–0.99 (m, 3H, -CH_2_-CH_2_ CH_2_-CH_3_), 1.36–1.38 (m, 2H, -CH_2_-CH_2_ CH_2_-CH_3_), 1.61 (m, 2H, -CH_2_-CH_2_ CH_2_-CH_3_), 2.34 (s, 3H, 7-CH_3_), 2.65 (s, 3H, 5-CH_3_), 3.06 (m, 4H, CH_2_—piperazine), 3.22 (m, 4H, CH_2_—piperazine), 3.46 (s, 3H, 3-CH_3_), 3.84–3.89 (m, 2H, -CH_2_-CH_2_ CH_2_-CH_3_), 5.16 (s, 2H, *N*-CH_2_-*N*) 5.30 (s, 2H, *O*-CH_2_-); 6.87–6.94 (m, 3H, Ar-H), 7.24–7.29 (m, 2H, Ar-H) 7.39 (m, 2H, Ar-H); 7.45–7.46 (m, 3H, Ar-H); ^13^C NMR (75 MHz, CDCl_3_) δ: 10.62, 11.23, 13.71, 20.03, 32.31, 36.97, 43.92, 49.36, 50.52, 57.09, 69.64, 108.05, 111.74, 116.39, 119.98, 122.18, 127.67 129.13, 129.63, 130.03, 133.80 146.79, 147.81, 151.31, 159.11, 170.52; MS (ESI-MS) (*m*/*z*): calcd. for C_33_H_40_N_8_O_2_S [L+H]^+^: 613.3068; found: 613.2995.

**5b:**6-butyl-3,5,7-trimethyl-1-[[4-phenyl-2-[[4-[3-(trifluoromethyl)phenyl]piperazin-1-yl]methyl]-3-thioxo-2*H*-1,2,4-triazol-5-yl]methoxy]pyrrolo[3,4-*d*]pyridazin-4-one

Yield: 65.29%; m.p.: 118–120 °C;

FT-IR (selected lines, γ_max_, cm^−1^): 2935, 2872, 2850 (C-H aliph.), 1268 (C=S); ^1^H NMR (300 MHz, CDCl_3_) δ: 0.94–0.99 (m, 3H, -CH_2_-CH_2_ CH_2_-CH_3_), 1.33–1.40 (m, 2H, -CH_2_-CH_2_ CH_2_-CH_3_), 1.59–1.61 (m, 2H, -CH_2_-CH_2_ CH_2_-CH_3_), 2.33 (s, 3H, 7-CH_3_), 2.65 (s, 3H, 5-CH_3_), 3.05–3.07 (m, 4H, CH_2_—piperazine), 3.24–3.26 (m, 4H, CH_2_—piperazine), 3.46 (s, 3H, 3-CH_3_), 3.84–3.89 (m, 2H, -CH_2_-CH_2_ CH_2_-CH_3_), 5.16 (s, 2H, *N*-CH_2_-*N*) 5.30 (s, 2H, *O*-CH_2_-); 7.04–7.09 (m, 3H, Ar-H), 7.32–7.39 (m, 3H, Ar-H); 7.46–7.48 (m, 3H, Ar-H); ^13^C NMR (75 MHz, CDCl_3_) δ: 10.62, 11.20, 13.69, 20.02, 32.31, 36.96, 43.92, 48.84, 50.34, 57.08, 69.54, 108.01, 111.72, 112.44, 116.01, 119.00, 122.15, 126.08, 127.65, 129.15, 129.60, 129.66, 130.07, 131.23, 131.65, 133.75, 146.87, 147.78, 151,37, 159.09, 170.52; MS (ESI-MS) (*m*/*z*): calcd. for C_34_H_39_F_3_N_8_O_2_S [L+H]^+^: 681.2942; found: 681.2864.

**6b:**6-butyl-3,5,7-trimethyl-1-[[4-phenyl-2-[[4-[(4-methyl)phenyl]piperazin-1-yl]methyl]-3-thioxo-2*H*-1,2,4-triazol-5-yl]methoxy]pyrrolo[3,4-*d*]pyridazin-4-one

Yield: 61.74%; m.p.: 108–111 °C;

FT-IR (selected lines, γ_max_, cm^−1^): 2959, 2935, 2860, 2832 (C-H aliph.), 1618 (C=N), 1234 (C=S); ^1^H NMR (300 MHz, CDCl_3_) δ: 0.94–0.99 (m, 3H, -CH_2_-CH_2_ CH_2_-CH_3_), 1.36–1.38 (m, 2H, -CH_2_-CH_2_ CH_2_-CH_3_), 1.61 (m, 2H, -CH_2_-CH_2_ CH_2_-CH_3_), 2.27 (s, 3H, Ar-CH_3_), 2.34 (s, 3H, 7-CH_3_), 2.66 (s, 3H, 5-CH_3_), 3.07 (m, 4H, CH_2_—piperazine), 3.18 (m, 4H, CH_2_—piperazine), 3.46 (s, 3H, 3-CH_3_), 3.84–3.90 (m, 2H, -CH_2_-CH_2_ CH_2_-CH_3_), 5.16 (s, 2H, *N*-CH_2_-*N*) 5.30 (s, 2H, *O*-CH_2_-); 6.86 (m, 2H, Ar-H), 7.06–7.09 (m, 2H, Ar-H); 7.39 (m, 2H, Ar-H), 7.45–7.46 (m, 3H, Ar-H); ^13^C NMR (75 MHz, CDCl_3_) δ: 10.63, 11.26, 13.71, 20.04, 32.32, 36.97, 43.92, 50.47, 57.09, 69.60, 108.02, 111.74, 116.84, 122.20, 127.68 129.11, 129.64, 129.69, 130.02, 133.80, 146.80, 147.81, 159.11, 170.52; MS (ESI-MS) (*m*/*z*): calcd. for C_34_H_42_N_8_O_2_S [L+H]^+^: 627.3224; found: 627.3176.

**4c:**6-butyl-1-[[4-(4-methoxyphenyl)-2-[(4-phenylpiperazin-1-yl)methyl]-3-thioxo-2*H*-1,2,4-triazol-5-yl]methoxy]-3,5,7-trimethyl-pyrrolo[3,4-*d*]pyridazin-4-one

Yield: 71.07%; m.p.: 169–170 °C;

FT-IR (selected lines, γ_max_, cm^−1^): 2961, 2933, 2860, 2838 (C-H aliph.), 1651 (C=N); ^1^H NMR (300 MHz, CDCl_3_) δ: 0.94–0.99 (m, 3H, -CH_2_-CH_2_ CH_2_-CH_3_), 1.34–1.41 (m, 2H, -CH_2_-CH_2_ CH_2_-CH_3_), 1.62 (m, 2H, -CH_2_-CH_2_ CH_2_-CH_3_), 2.36 (s, 3H, 7-CH_3_), 2.66 (s, 3H, 5-CH_3_), 3.05–3.07 (m, 4H, CH_2_—piperazine), 3.20–3.22 (m, 4H, CH_2_—piperazine), 3.47 (s, 3H, 3-CH_3_), 3.81 (s, 3H, *O*-CH_3_) 3.85–3.90 (m, 2H, -CH_2_-CH_2_ CH_2_-CH_3_), 5.15 (s, 2H, *N*-CH_2_-*N*) 5.29 (s, 2H, *O*-CH_2_-); 6.84–6.95 (m, 5H, Ar-H), 7.24–7.28 (m, 4H, Ar-H); ^13^C NMR (75 MHz, CDCl_3_) δ: 10.62, 11.26, 13.72, 20.05, 32.33, 36.97, 43.94, 49.36, 50.52, 55.51, 57.11, 69.66, 108.08, 111.74, 114.83, 116.39, 119.97, 122.19, 126.21 129.13, 147.11, 147.88, 151.31, 159.11, 160.48, 170.75; HRMS (ESI-MS) (*m*/*z*): calcd. for C_34_H_42_N_8_O_3_S [L+H]^+^: 643.3173; found: 643.3194.

**5c:**6-butyl-1-[[4-(4-methoxyphenyl)-2-[[4-[3-(trifluoromethyl)phenyl]piperazin-1-yl]methyl]-3-thioxo-2*H*-1,2,4-triazol-5-yl]methoxy]-3,5,7-trimethyl-pyrrolo[3,4-*d*]pyridazin-4-one

Yield: 59.73%; m.p.: 163–165 °C;

FT-IR (selected lines, γ_max_, cm^−1^): 2964, 2935, 2870, 2840 (C-H aliph.), 1650 (C=N), 1248 (C=S); ^1^H NMR (300 MHz, CDCl_3_) δ: 0.94–0.99 (m, 3H, -CH_2_-CH_2_ CH_2_-CH_3_), 1.34–1.41 (m, 2H, -CH_2_-CH_2_ CH_2_-CH_3_), 1.62 (m, 2H, -CH_2_-CH_2_ CH_2_-CH_3_), 2.36 (s, 3H, 7-CH_3_), 2.66 (s, 3H, 5-CH_3_), 3.04 (m, 4H, CH_2_—piperazine), 3.24–3.26 (m, 4H, CH_2_—piperazine), 3.47 (s, 3H, 3-CH_3_), 3.81 (s, 3H, *O*-CH_3_), 3.85–3.90 (m, 2H, -CH_2_-CH_2_ CH_2_-CH_3_), 5.15 (s, 2H, *N*-CH_2_-*N*) 5.29 (s, 2H, *O*-CH_2_-); 6.92–6.95 (m, 2H, Ar-H), 7.04–7.09 (m, 3H, Ar-H), 7.29–7.37 (m, 3H, Ar-H); ^13^C NMR (75 MHz, CDCl_3_) δ: 10.61, 11.20, 13.68, 20.03, 32.32, 36.95, 43.93, 48.83, 50.34, 55.51, 57.10, 69.57, 108.09, 111.75, 112.42, 114.84, 116.04, 118.98, 122.15, 126.18, 128.83, 129.14, 129.59, 131.24, 147.18, 147.85, 151.37, 159.09, 160.51, 170.78; HRMS (ESI-MS) (*m*/*z*): calcd. for C_35_H_41_F_3_N_8_O_3_S [L+H]^+^: 711.3047; found: 711.3031.

**6c:**6-butyl-1-[[4-(4-methoxyphenyl)-2-[[4-[(4-methyl)phenyl]piperazin-1-yl]methyl]-3-thioxo-2*H*-1,2,4-triazol-5-yl]methoxy]-3,5,7-trimethyl-pyrrolo[3,4-*d*]pyridazin-4-one

Yield: 74.21%; m.p.: 151–153 °C;

FT-IR (selected lines, γ_max_, cm^−1^): 2958, 2934, 2860, 2837 (C-H aliph.), 1651 (C=N), 1247 (C=S); ^1^H NMR (300 MHz, CDCl_3_) δ: 0.94–0.99 (m, 3H, -CH_2_-CH_2_ CH_2_-CH_3_), 1.36–1.41 (m, 2H, -CH_2_-CH_2_ CH_2_-CH_3_), 1.62 (m, 2H, -CH_2_-CH_2_ CH_2_-CH_3_), 2.27 (s, 3H, Ar-CH_3_), 2.37 (s, 3H, 7-CH_3_), 2.66 (s, 3H, 5-CH_3_), 3.07 (m, 4H, CH_2_—piperazine), 3.17 (m, 4H, CH_2_—piperazine), 3.47 (s, 3H, 3-CH_3_), 3.81 (s, 3H, *O*-CH_3_) 3.85–3.90 (m, 2H, -CH_2_-CH_2_ CH_2_-CH_3_), 5.15 (s, 2H, *N*-CH_2_-*N*) 5.29 (s, 2H, *O*-CH_2_-); 6.85 (m, 2H, Ar-H), 6.92–6.95 (m, 2H, Ar-H), 7.07–7.09 (m, 2H, Ar-H), 7.25–7.29 (m, 2H, Ar-H); ^13^C NMR (75 MHz, CDCl_3_) δ: 10.63, 11.28, 13.72, 20.05, 20.46, 32.33, 36.97, 43.94, 50.43, 55.51, 57.11, 69.61, 108.08, 111.74, 114.82, 116.86, 117.24, 122.21, 126.22, 128.85, 129.10, 129.71, 147.12, 147.88, 159.11, 160.48, 170.75; HRMS (ESI-MS) (*m*/*z*): calcd. for C_35_H_44_N_8_O_3_S [L+H]^+^: 657.3330; found: 657.3316.

##### General Procedure for Preparation of S-Substituted Derivatives of Pyrrolo[3,4-*d*]pyridazinone (**7a-c-9a-c**)

The appropriate 4-substituted-1,2,4-triazole derivative of pyrrolo[3,4-*d*]pyridazinone (**3a**, **3b** or **3c**) (0.001 mol) was suspended in 30 mL of anhydrous ethanol in a round bottom flask. Next, the 1 mL of 1M sodium ethoxide (0.001 mol) and the appropriate 2-chloro-1-oxoethyl aryl piperazine derivative was added, and the mixture was refluxed for 4–6 h. TLC monitored the reaction progress. After the completion of synthesis, the mixture was cooled, and the precipitate was formed. Finally, the solid was filtered off, washed thoroughly with ethanol and, afterward, purified by crystallization from this solvent.

**7a:**6-butyl-3,5,7-trimethyl-1-[[4-methyl-3-[2-oxo-2-(4-phenylpiperazin-1-yl)ethyl]sulfanyl-2*H*-1,2,4-triazol-5-yl]methoxy]pyrrolo[3,4-*d*]pyridazin-4-one

Yield: 62.52%; m.p.: 183–184 °C;

FT-IR (selected lines, γ_max_, cm^−1^): 3054 (C-H arom.), 2961, 2922, 2873 (C-H aliph.), 1639 (C=O), 1268 (C=S); ^1^H NMR (300 MHz, CDCl_3_) δ: 0.93–0.98 (m, 3H, -CH_2_-CH_2_ CH_2_-CH_3_), 1.33–1.41 (m, 2H, -CH_2_-CH_2_ CH_2_-CH_3_), 1.59–1.62 (m, 2H, -CH_2_-CH_2_ CH_2_-CH_3_), 2.41 (s, 3H, 7-CH_3_), 2.68 (s, 3H, 5-CH_3_), 3.17–3.19 (m, 2H, CH_2_—piperazine), 3.23 (m, 2H, CH_2_—piperazine), 3.57 (s, 3H, 3-CH_3_), 3.66 (s, 3H, triazole-*N*-CH_3_), 3.78 (m, 4H, CH_2_—piperazine), 3.86–3.91 (m, 2H, -CH_2_-CH_2_ CH_2_-CH_3_), 4.38 (s, 2H, *S*-CH_2_) 5.44 (s, 2H, *O*-CH_2_-); 6.92–6.94 (m, 3H, Ar-H), 7.28–7.31 (m, 2H, Ar-H); ^13^C NMR (75 MHz, CDCl_3_) δ: 10.64, 11.21, 13.68, 20.04, 30.54, 32.32, 36.57, 37.02, 42.21, 43.93, 46.11, 49.33, 49.74, 57.45, 108.27, 111.86, 116.80, 120.80, 122.29, 129.19, 129.29, 148.33, 151.80, 152.30, 159,19, 165.55; HRMS (ESI-MS) (*m*/*z*): calcd. for C_29_H_38_N_8_O_3_S [L+H]^+^: 579.2860; found: 579.2843.

**8a:**6-butyl-3,5,7-trimethyl-1-[[4-methyl-3-[2-oxo-2-[4-[3-(triflouromethyl)phenyl]piperazin-1-yl]ethyl]sulfanyl-2*H*-1,2,4-triazol-5-yl]methoxy]pyrrolo[3,4-*d*]pyridazin-4-one

Yield: 56.41%; m.p.: 201–202 °C;

FT-IR (selected lines, γ_max_, cm^−1^): 2964, 2934, 2919 (C-H aliph.) 1226 (C=S); ^1^H NMR (300 MHz, CDCl_3_) δ: 0.93–0.98 (m, 3H, -CH_2_-CH_2_ CH_2_-CH_3_), 1.33–1.41 (m, 2H, -CH_2_-CH_2_ CH_2_-CH_3_), 1.59–1.62 (m, 2H, -CH_2_-CH_2_ CH_2_-CH_3_), 2.42 (s, 3H, 7-CH_3_), 2.68 (s, 3H, 5-CH_3_), 3.23 (m, 2H, CH_2_—piperazine), 3.31 (m, 2H, CH_2_—piperazine), 3.57 (s, 3H, 3-CH_3_), 3.68 (s, 3H, triazole-*N*-CH_3_), 3.80–3.84 (m, 4H, CH_2_—piperazine), 3.86–3.91 (m, 2H, -CH_2_-CH_2_ CH_2_-CH_3_), 4.41 (s, 2H, *S*-CH_2_) 5.44 (s, 2H, *O*-CH_2_-); 7.08–7.16 (m, 3H, Ar-H), 7.35–7.41 (m, 1H, Ar-H); ^13^C NMR (75 MHz, CDCl_3_) δ: 10.64, 11.22, 13.68, 20.04, 30.68, 32.32, 36.56, 37.02, 42.01, 43.94, 45.90, 48.85, 49.21, 57.33, 108.21, 111.83, 112.97, 117.10, 119.54, 122.29, 129.25, 129.79, 131.43, 131.85, 148.26, 150.69, 151.83, 152.31, 159,17, 165.51; MS (ESI-MS) (*m*/*z*): calcd. for C_30_H_37_F_3_N_8_O_3_S [L+H]^+^: 647.2734; found: 647.2680.

**9a:**6-butyl-3,5,7-trimethyl-1-[[4-methyl-3-[2-oxo-2-[4-[4-(methyl)phenyl]piperazin-1-yl]ethyl]sulfanyl-2*H*-1,2,4-triazol-5-yl]methoxy]pyrrolo[3,4-*d*]pyridazin-4-one

Yield: 59.49%; m.p.: 190–191 °C;

FT-IR (selected lines, γ_max_, cm^−1^): 2960, 2919, 2851 (C-H aliph.), 1643 (C=O) 1227 (C=S); ^1^H NMR (300 MHz, CDCl_3_) δ: 0.93–0.98 (m, 3H, -CH_2_-CH_2_ CH_2_-CH_3_), 1.34–1.41 (m, 2H, -CH_2_-CH_2_ CH_2_-CH_3_), 1.62 (m, 2H, -CH_2_-CH_2_ CH_2_-CH_3_), 2.27 (s, 3H, Ar-CH_3_), 2.41 (s, 3H, 7-CH_3_), 2.68 (s, 3H, 5-CH_3_), 3.11 (m, 4H, CH_2_—piperazine), 3.57 (s, 3H, 3-CH_3_), 3.66 (s, 3H, triazole-*N*-CH_3_), 3.78 (m, 4H, CH_2_—piperazine), 3.86–3.91 (m, 2H, -CH_2_-CH_2_ CH_2_-CH_3_), 4.38 (s, 2H, *S*-CH_2_) 5.44 (s, 2H, *O*-CH_2_-); 6.86 (m, 2H, Ar-H), 7.08–7.11 (m, 2H, Ar-H); ^13^C NMR (75 MHz, CDCl_3_) δ: 10.64, 11.22, 13.68, 20.04, 20.45 30.54, 32.32, 36.67, 37.03, 42.26, 43.93, 46.16, 49.88, 50.27, 57.46, 108.28, 111.87, 117.14, 122.29, 129.19, 129.81, 148.33, 151.84, 152.29, 159,19, 165.50; MS (ESI-MS) (*m*/*z*): calcd. for C_30_H_40_N_8_O_3_S [L+H]^+^: 593.3017; found: 593.3092.

**7b:**6-butyl-3,5,7-trimethyl-1-[[4-phenyl-3-[2-oxo-2-(4-phenylpiperazin-1-yl)ethyl]sulfanyl-2*H*-1,2,4-triazol-5-yl]methoxy]pyrrolo[3,4-*d*]pyridazin-4-one

Yield: 53.83%; m.p.: 126–128 °C;

FT-IR (selected lines, γ_max_, cm^−1^): 3053 (C-H arom.), 2929, 2871 (C-H aliph.), 1636 (C=O); ^1^H NMR (300 MHz, CDCl_3_) δ: 0.94–0.99 (m, 3H, -CH_2_-CH_2_ CH_2_-CH_3_), 1.33–1.41 (m, 2H, -CH_2_-CH_2_ CH_2_-CH_3_), 1.59–1.61 (m, 2H, -CH_2_-CH_2_ CH_2_-CH_3_), 2.33 (s, 3H, 7-CH_3_), 2.66 (s, 3H, 5-CH_3_), 3.17 (m, 2H, CH_2_—piperazine), 3.23 (m, 2H, CH_2_—piperazine), 3.46 (s, 3H, 3-CH_3_), 3.78 (m, 4H, CH_2_—piperazine), 3.84–3.89 (m, 2H, -CH_2_-CH_2_ CH_2_-CH_3_), 4.42 (s, 2H, *S*-CH_2_) 5.31 (s, 2H, *O*-CH_2_-); 6.89–6.94 (m, 3H, Ar-H), 7.29–7.34 (m, 4H, Ar-H), 7.44–7.46 (m, 3H, Ar-H); ^13^C NMR (75 MHz, CDCl_3_) δ: 10.61, 11.19, 13.70, 20.04, 32.32, 36.32, 42.18, 43.87, 46.13, 49.26, 49.72, 56.94, 108.24, 111.78, 116.76, 120.72, 122.21, 126.65, 129.27, 129.91, 130.21, 132.60, 148.15, 150.76, 152.28, 159,13, 165.54; MS (ESI-MS) (*m*/*z*): calcd. for C_34_H_40_N_8_O_3_S [L+H]^+^: 641.3017; found: 641.2935.

**8b:**6-butyl-3,5,7-trimethyl-1-[[4-phenyl-3-[2-oxo-2-[4-[3-(triflouromethyl)phenyl]piperazin-1-yl]ethyl]sulfanyl-2*H*-1,2,4-triazol-5-yl]methoxy]pyrrolo[3,4-*d*]pyridazin-4-one

Yield: 60.84%; m.p.: 141–143 °C;

FT-IR (selected lines, γ_max_, cm^−1^): 3053 (C-H arom.), 2959, 2930, 2872 (C-H aliph.), 1635 (C=O); ^1^H NMR (300 MHz, CDCl_3_) δ: 0.94–0.99 (m, 3H, -CH_2_-CH_2_ CH_2_-CH_3_), 1.33–1.40 (m, 2H, -CH_2_-CH_2_ CH_2_-CH_3_), 1.59–1.61 (m, 2H, -CH_2_-CH_2_ CH_2_-CH_3_), 2.33 (s, 3H, 7-CH_3_), 2.65 (s, 3H, 5-CH_3_), 3.22 (m, 2H, CH_2_—piperazine), 3.30 (m, 2H, CH_2_—piperazine), 3.45 (s, 3H, 3-CH_3_), 3.81–3.84 (m, 4H, CH_2_—piperazine), 3.86–3.89 (m, 2H, -CH_2_-CH_2_ CH_2_-CH_3_), 4.39 (s, 2H, *S*-CH_2_) 5.31 (s, 2H, *O*-CH_2_-); 7.06–7.15 (m, 3H, Ar-H), 7.31–7.35 (m, 2H, Ar-H), 7.37–7.40 (m, 1H, Ar-H), 7.44–7.46 (m, 3H, Ar-H); ^13^C NMR (75 MHz, CDCl_3_) δ: 10.61, 11.18, 13.69, 20.03, 32.31, 36.02, 36.94, 41.98, 43.87, 45.92, 48.76, 49.17, 56.92, 108.21, 108.23, 111.78, 112.93, 116.94, 119.44, 122.20, 125.95, 126.64, 128.92, 129.76, 129.92, 130.25, 131.43, 131.86, 132.57, 148.13. 150.85, 152.34, 152.34, 159.13, 165.65; MS (ESI-MS) (*m*/*z*): calcd. for C_35_H_39_F_3_N_8_O_3_S [L+H]^+^: 708.2891; found: 708.2827.

**9b:**6-butyl-3,5,7-trimethyl-1-[[4-phenyl-3-[2-oxo-2-[4-[4-(methyl)phenyl]piperazin-1-yl]ethyl]sulfanyl-2*H*-1,2,4-triazol-5-yl]methoxy]pyrrolo[3,4-*d*]pyridazin-4-one

Yield: 55.89%; m.p.: 129–131 °C;

FT-IR (selected lines, γ_max_, cm^−1^): 2958, 2925, 2870 (C-H aliph.), 1639 (C=O); ^1^H NMR (300 MHz, CDCl_3_) δ: 0.94–0.99 (m, 3H, -CH_2_-CH_2_ CH_2_-CH_3_), 1.35–1.38 (m, 2H, -CH_2_-CH_2_ CH_2_-CH_3_), 1.61 (m, 2H, -CH_2_-CH_2_ CH_2_-CH_3_), 2.28 (s, 3H, Ar-CH_3_), 2.33 (s, 3H, 7-CH_3_), 2.65 (s, 3H, 5-CH_3_), 3.11–3.16 (m, 4H, CH_2_—piperazine), 3.45 (s, 3H, 3-CH_3_), 3.76 (m, 4H, CH_2_—piperazine), 3.84–3.89 (m, 2H, -CH_2_-CH_2_ CH_2_-CH_3_), 4.41 (s, 2H, *S*-CH_2_) 5.31 (s, 2H, *O*-CH_2_-); 6.83–6.86 (m, 2H, Ar-H), 7.08–7.11 (m, 2H, Ar-H), 7.33–7.34 (m, 2H, Ar-H), 7.43–7.46 (m, 3H, Ar-H); ^13^C NMR (75 MHz, CDCl_3_) δ:10.60, 10.98, 11.19, 13.69, 20.02, 20.43, 32.31, 36.39, 36.94, 42.23, 43.87, 46.17, 49.81, 50.28, 56.94, 108.25, 111.78, 117.111, 122.21, 126.76, 127.16, 128.89, 129.78, 129.90, 130.19, 130.36, 132.61, 133.46, 148.16, 148.65, 152.25, 159.13, 165.49; MS (ESI-MS) (*m*/*z*): calcd. for C_35_H_42_N_8_O_3_S [L+Na]^+^: 677.2993; found: 677.2945.

**7c:**6-butyl-3,5,7-trimethyl-1-[[4-(4-methoxy)phenyl-3-[2-oxo-2-(4-phenylpiperazin-1-yl)ethyl]sulfanyl-2*H*-1,2,4-triazol-5-yl]methoxy]pyrrolo[3,4-*d*]pyridazin-4-one

Yield: 59.71%; m.p.: 150–152 °C;

FT-IR (selected lines, γ_max_, cm^−1^): 3060, (C-H arom.), 2957, 2929, 2871 (C-H aliph.), 1640 (C=O), 1271 (C=S); ^1^H NMR (300 MHz, CDCl_3_) δ: 0.94–0.99 (m, 3H, -CH_2_-CH_2_ CH_2_-CH_3_), 1.36–1.39 (m, 2H, -CH_2_-CH_2_ CH_2_-CH_3_), 1.61 (m, 2H, -CH_2_-CH_2_ CH_2_-CH_3_), 2.36 (s, 3H, 7-CH_3_), 2.66 (s, 3H, 5-CH_3_), 3.16 (m, 2H, CH_2_—piperazine), 3.23 (m, 2H, CH_2_—piperazine), 3.47 (s, 3H, 3-CH_3_), 3.78 (m, 4H, CH_2_—piperazine), 3.81 (s, 3H, *O*-CH_3_), 3.84–3.89 (m, 2H, -CH_2_-CH_2_ CH_2_-CH_3_), 4.40 (s, 2H, *S*-CH_2_) 5.28 (s, 2H, *O*-CH_2_-); 6.89–6.94 (m, 5H, Ar-H), 7.21–7.31 (m, 4H, Ar-H); ^13^C NMR (75 MHz, CDCl_3_) δ: 10.62, 11.23, 13.71, 20.05, 32.32, 36.22, 36.95, 42.16, 43.89, 46.12, 49.25, 49.72, 55.59, 56.94, 108.29, 111.78, 114.99, 116.75, 120.71, 122.23, 124.91, 127.97, 128.87, 129.28, 148.24, 150.76, 152.55, 153.09, 159,13, 160.71, 165.58; HRMS (ESI-MS) (*m*/*z*): calcd. for C_35_H_42_N_8_O_4_S [L+Na]^+^: 693.2942; found: 693.2956.

**8c:**6-butyl-3,5,7-trimethyl-1-[[4-(4-methoxy)phenyl-3-[2-oxo-2-[4-[3-(triflouromethyl)phenyl]piperazin-1-yl]ethyl]sulfanyl-2*H*-1,2,4-triazol-5-yl]methoxy]pyrrolo[3,4-*d*]pyridazin-4-one

Yield: 69.10%; m.p.: 114–117 °C;

FT-IR (selected lines, γ_max_, cm^−1^): 2959, 2929, 2871 (C-H aliph.), 1644 (C=O); ^1^H NMR (300 MHz, CDCl_3_) δ: 0.94–0.99 (m, 3H, -CH_2_-CH_2_ CH_2_-CH_3_), 1.33–1.41 (m, 2H, -CH_2_-CH_2_ CH_2_-CH_3_), 1.62 (m, 2H, -CH_2_-CH_2_ CH_2_-CH_3_), 2.36 (s, 3H, 7-CH_3_), 2.66 (s, 3H, 5-CH_3_), 3.22 (m, 2H, CH_2_—piperazine), 3.29 (m, 2H, CH_2_—piperazine), 3.46 (s, 3H, 3-CH_3_), 3.81 (s, 3H, *O*-CH_3_), 3.84 (m, 4H, CH_2_—piperazine), 3.86–3.89 (m, 2H, -CH_2_-CH_2_ CH_2_-CH_3_), 4.37 (s, 2H, *S*-CH_2_) 5.28 (s, 2H, *O*-CH_2_-); 7.89–6.92 (m, 2H, Ar-H), 7.05–7.14 (m, 3H, Ar-H), 7.21–7.24 (m, 2H, Ar-H), 7.35–7.40 (m, 1H, Ar-H); ^13^C NMR (75 MHz, CDCl_3_) δ: 10.59, 11.21, 13.69, 20.03, 32.32, 35.86, 36.94, 41.98, 43.88, 45.92, 48.72, 49.14, 55.57 56.92, 108.30 111.79, 112.88, 114.99, 116.87, 119.39, 122.21, 124.91, 127.97, 128.90, 129.75, 131.42, 131.84, 148.22, 150.89, 152.62, 152.91, 159.12, 160.74, 165.72; HRMS (ESI-MS) (*m*/*z*): calcd. for C_36_H_41_F_3_N_8_O_4_S [L+Na]^+^: 761.2816; found: 761.2812.

**9c:**6-butyl-3,5,7-trimethyl-1-[[4-(4-methoxy)phenyl-3-[2-oxo-2-[4-[4-(methyl)phenyl]piperazin-1-yl]ethyl]sulfanyl-2*H*-1,2,4-triazol-5-yl]methoxy]pyrrolo[3,4-*d*]pyridazin-4-one

Yield: 67.44%; m.p.: 119–121 °C;

FT-IR (selected lines, γ_max_, cm^−1^): 3052, 3002 (C-H arom.), 2957, 2922, 2871 (C-H aliph.), 1643 (C=O); ^1^H NMR (300 MHz, CDCl_3_) δ: 0.94–0.99 (m, 3H, -CH_2_-CH_2_ CH_2_-CH_3_), 1.34–1.41 (m, 2H, -CH_2_-CH_2_ CH_2_-CH_3_), 1.59–1.61 (m, 2H, -CH_2_-CH_2_ CH_2_-CH_3_), 2.28 (s, 3H, Ar-CH_3_), 2.36 (s, 3H, 7-CH_3_), 2.66 (s, 3H, 5-CH_3_), 3.10 (m, 2H, CH_2_—piperazine), 3.16 (m, 2H, CH_2_—piperazine), 3.46 (s, 3H, 3-CH_3_), 3.78 (m, 4H, CH_2_—piperazine), 3.81 (s, 3H, *O*-CH_3_), 3.84–3.89 (m, 2H, -CH_2_-CH_2_ CH_2_-CH_3_), 4.40 (s, 2H, *S*-CH_2_) 5.28 (s, 2H, *O*-CH_2_-); 6.83–6.2 (m, 4H, Ar-H), 7.08–7.11 (m, 2H, Ar-H), 7.21–7.24 (m, 2H, Ar-H); ^13^C NMR (75 MHz, CDCl_3_) δ:10.61, 11.22, 13.69, 20.04, 20.44, 32.32, 36.28, 36.95, 42.22, 43.88, 46.18, 49.81, 50.29, 55.58, 56.94, 108.30, 111.79, 114.99, 117.10, 122.22, 124.94, 127.97, 128.87, 129.79, 130.35, 148.24, 148.66, 152.54, 153.11, 159.13, 160.72, 165.54; HRMS (ESI-MS) (*m*/*z*): calcd. for C_36_H_44_N_8_O_4_S [L+H]^+^: 685.3279; found: 685.3246.

### 3.2. Biological Evaluation

#### 3.2.1. Cell Line and Conditions

Normal human dermal fibroblasts (NHDF) were purchased from Lonza and used in bioassays between 7–12 passages. The cells were incubated in 5% CO_2_, 95% humidity at 37 °C with morphology and confluence assessments twice weekly using EVOS FL microscopy. The cells were passaged with TrypLE solution when the cell confluence was greater than 70%. The cells were transferred to a tube and centrifuged at 1000× *g* for 5 min. Then, the supernatant was removed, and the cells were resuspended in a fresh medium, and the number of cells was counted using the Burcher chamber. Finally, the cells were plated on the assay plates or reduced by about half and placed back in the culture flasks. The NHDF cells were grown in Dulbecco’s modified Eagle’s medium (DMEM) without phenol red supplemented with 10% fetal bovine serum (FBS), 2 mM ultra-glutamine and 2 µg/mL gentamicin and streptomycin. The medium was stored at 4–8 °C for one month or until used.

#### 3.2.2. Tested Compounds

The tested compounds were dissolved in dimethyl sulfoxide (DMSO) to form 10 mM stock solutions which were stored at −20 °C. These compounds were thawed immediately before the preparation of the bioassay concentrations. The concentration range of 10–100 µM was used, so the DMSO concentration did not exceed 1% at the higher concentration tested. The bioassay concentrations were prepared in DMEM without phenol red, which was supplemented as a medium for traditional cultures, but with a reduced amount of FBS to 5%.

#### 3.2.3. Cyclooxygenase Inhibition Assay

The cyclooxygenase (COX) inhibition was evaluated using a ready-to-used test from the Cayman company. In this study, the only 100 µM concentrations that were tested, each in triplicate, for the obtained results were calculated IC_50_—concentration, which inhibited activity of COX-1 or COX-2 about 50% compared to 100% activity of these enzymes. In these studies, *Meloxicam, Diclofenac* and *Celecoxib* were used as reference compounds.

#### 3.2.4. MTT Assay

The assessment of cell viability after 24-hour incubation with the test compounds was performed according to ISO 10993 Part 5 Appendix C. The cells were seeded at 10,000 cells per well and left in a CO_2_ incubator overnight to allow the cells to adhere. Non-adherent cells were removed with the medium, and freshly prepared concentrations of test compounds were added for 24 h. During the last hour of 24-hour incubation, the cells were assessed microscopically according to the ISO 10,993 scale for cytotoxicity. The medium with the compounds was replaced with a 1 mg/mL MTT in phosphate buffered saline (PBS) for 2 h at 37 °C. The solution was then gently removed and the purple crystals dissolved in isopropanol, and the absorbance was measured at 570 nm with a VirusScan microplate reader.

#### 3.2.5. Anti-Inflammatory and Antioxidant Activity

To evaluate the anti-inflammatory and antioxidant activity of the tested compounds, the MTT, DCF-DA and Griess assays were performed. In the first assay, the NHDF cells were seeded at a density of 10,000 cells per well, and in the other assay, 40,000 cells per well. After the cells adhered overnight, the supernatant with the non-adherent cells was replaced with 50 µg/mL lipopolysaccharide (LPS) for 24 h. Next, the cells were washed, and freshly prepared concentrations of the test compounds were added for 24 h. Then, the culture plate was washed for MTT assay, and the procedure described in Section 3.2.4. was used. To evaluate the free radicals scavenging of tested the compounds, the 50 µL supernatant was transferred to a new plate. The rest of the supernatant was removed and 25 µM of DCF-DA solution in MEM without phenol red was added for 1 h at 37 °C to measure the reactive oxygen species (ROS) and into collected supernatant 50 µM mixture reagent A and reagent B in a volume ratio 1:1 for 20 min at RT in the dark to measure the nitric oxide (NO). The ROS was measured at 498 nm excitation and 535 nm emission, and NO at 548 nm using a VirusScan microplate reader.

### 3.3. Molecular Docking

The structure optimization was performed using the DFT/B3LYP method combined with the 6–311+G (d,p) basis set. From the Protein Data Bank (http://www.rcsb.org, accessed on 1 May 2021), the following crystal structure was selected for the docking studies: 4O1Z, 4M11, 3V03. The ligand and receptor files were prepared using AutoDock 4.2.6 software and AutoDock Tools 1.5.6. All of the ligands and water molecules were removed, and then polar hydrogen atoms and Kollman charges were added to the protein structure. To prepare the ligand molecules, the partial charges were calculated, non-polar hydrogens were merged, and rotatable bonds were assigned. The interactions with COX-1, COX-2 and BSA were performed using AutoDock Script downloaded from The Scripps Research Institute (TSRI). The centers of the grid boxes for COX-1 and COX-2 were set according to the *Meloxicam* binding site in the crystal structure 4O1Z, 4M11. The centers of the grid boxes for BSA were set according to the binding site I phenylbutazone (PDB ID: 2BXC) and site II *Ibuprofen* (PDB ID: 2BXG) on HSA [58]. The Lamarckian genetic algorithm was selected for the conformational search. The running times of the genetic algorithm and the evaluation times were set to 100 and 2.5million, respectively. After the molecular docking, the ligand–receptor complexes were further analyzed using Discovery Studio software (http://accelrys.com/, accessed on 1 May 2021).

### 3.4. Spectroscopic Studies

#### 3.4.1. Fluorescence

The spectroscopic fluorescence studies were performed using a Cary Eclipse 500 spectrophotometer (Agilent, Santa Clara, CA, USA). A concentration of BSA was 1.0 × 10^−6^ mol·dm^−3^. A solution of BSA was titrated by successive additions of 1.0 × 10^−3^ mol·dm^−3^ solution of the studied compounds to give a final concentration of 0.2 × 10^−6^–2.0 × 10^−6^ mol·dm^−3^. Experiments were carried out at three temperatures: 297, 303, and 308 K in pH = 7.4. The quenching spectra were recorded at excitation and an emission wavelength of 280 nm and 300–500 nm. The molar ratio compound/BSA was 0.1–2.0 with 0.2 steps. Binding site identification studies were indicated in the presence of the two site markers, *Phenylbutazone* (PHB) and *Ibuprofen* (IBP), as sites I and II markers, respectively. Concentrations of BSA and site markers were set at 1.0 × 10^−6^ and 3.0 × 10^−6^ mol·dm^−3^, respectively.

#### 3.4.2. Circular Dichroism

Circular dichroism (CD) spectra were measured on the Jasco J-1500 magnetic circular dichroism spectrometer. All of the measurements for the BSA solutions in the absence and presence of the analyzed compounds were made at room temperature under simulated physiological conditions in pH 7.4, in phosphate buffer as a solvent. The CD spectra were collected in the range of 205–250 nm at a scan rate speed of 50 nm min^−1^, with a response time of 1 s, 10 mm path length, and were baseline corrected. The concentrations of BSA and the analyzed compounds were 1 × 10^−6^ mol·dm^−3^ and 1 × 10^−3^ mol·dm^−3^, respectively. BSA performed experiments on each analyzed compound in molar ratios: 1:0, 1:0.5, 1:1, 1:5 and 1:10.

#### 3.4.3. FT-IR Measurement

Infrared spectra were recorded on the Nicolet iS50 FT-IR (Thermo Fisher Scientific, Waltham, MA, USA) equipped with a deuterated triglycine sulphate (DTGS) detector and KBr beam splitter. The spectra were obtained at room temperature using the attenuated total reflectance (ATR) method. The spectral data were recorded within 4000 to 600 cm^−1^ with resolution 4 cm^−1^ and 100 scans were averaged for each spectrum.

Bovine serum albumin (Sigma Aldrich) was dissolved in an aqueous solution containing phosphate buffer (pH = 7.5) (Sigma Aldrich) to obtain 0.02 mol·dm^−1^ concentration. The concentration of the studied compounds was 0.01 mol·dm^−1^ andsolutions were prepared in methanol (Chempur). The 200 μL solution of BSA was mixed with the appropriate amount of the compounds solution to achieve 0.25, 0.50, 0.75 and 1.0 molar ratio and 10 μL of mixture was dropped on the crystal to register a spectrum.

The analysis of the secondary structure was proceeded by Omnic 9.3.30 (Thermo Fisher Scientific Inc.) software. The analysis of the FT-IR spectra was evaluated by the Byler and Susi procedure [56]. After normalization of each spectrum, the fragment with the peak 1650 cm^−1^ was extracted, and a second derivate was made. The major peaks are characteristic for the α-helix (1660–1650 cm^−1^), β-sheet (1640–1610 cm^−1^), β-turn (1691–1680 cm^−1^), β-antiparallel (1660–1650 cm^−1^) and random coil (1650–1640 cm^−1^) [56,62,63,64]. The self-deconvolution and curve-fitting by Gaussian function allowed for us to determine the intensity and total area under peaks. The calculation of the percentage of area peaks corresponds with the contribution of the type of structure.

### 3.5. Statistical Analysis

The biological results are shown again as the mean ± SD of the IC_50_ in the cyclooxygenase inhibition assay and the E/E_0_ ratio in the remaining assays. E is the mean result for the compounds tested, and E_0_ is the mean result for the controls. For the viability assay, E_0_ cells were incubated only with a medium without the test compounds. In assessing the anti-inflammatory activity of the tested compounds, cells treated with only 50 µg/mL LPS were controls. The statistical analyses were performed using the Statistica program. One-way ANOVA and Tukey post-hoc analysis were calculated. The *p*-value was set at 0.05.

## 4. Conclusions

The present paper describes the design, synthesis and complex biological, computational and also spectroscopic studies of novel, three series of *N*-substituted-1,2,4-triazole-based derivatives of pyrrolo[3,4-*d*]pyridazinone. The structures of the title compounds were inspired by the results of our previous investigations and most recent literature reports. Their formation relied on the molecular hybridization of the biheterocyclic scaffold of pyrrolo[3,4-*d*]pyridazinone, *N*-substituted-1,2,4-triazole moiety and aryl piperazine pharmacophore (Scheme 1, Figure 7). Our goal was to receive potent anti-inflammatory agents with possibly the best affinity towards an inducible COX-2 isoform. The evaluation of the viability revealed that five compounds **8a-c**, **9b**, **9c** cause cell lysis and were excluded from further examination. The results of in vitro COX inhibition assay indicate that molecules **4a-7a**, **9a**, **4b**, **7b** and **4c** have good, comparable to *Meloxicam*, inhibitory activity towards COX-2 isoenzyme and are characterized by a promising COX-2/COX-1 selectivity ratio. Moreover, derivatives **4a**, **6a**, **9a** and **4c** acted as selective COX-2 inhibitors. These findings were supported by the results of the molecular docking studies, according to which, new molecules take position in the active site of COX-2 very similar to *Meloxicam* and did not show an affinity to COX-1 isoform. What is more, the potential good anti-inflammatory and antioxidant activity of the examined derivatives was confirmed in the performed in vitro evaluation within cells. As it has already been stated, all molecules bind and interact with serum albumin, which is the most abundant protein in blood. Some of them, especially those belonging to series **a**, exert promising and beneficial properties in the executed in silico ADME prediction.

Taking the above information into account, we can summarize that title *N*-substituted-1,2,4-triazole-based derivatives of pyrrolo[3,4-*d*]pyridazinone can serve as promising and valuable structures in the development of novel anti-inflammatory agents. Undoubtedly, further, extended investigations, especially in vivo experiments, concerning these compounds are necessary. Moreover, based on the described biological, computational, structure–activity relationship (SAR) and ADME study, we are going to perform rational structural modifications of the reported derivatives in order to receive a new series of potent and effective molecules.

## Figures and Tables

**Table 1:** The cell viability [IC_50_ (SEM) n = 3] and the evaluation of the morphology of cells treated with the tested compounds.

	IC_50_ [µM]	Cell Morphology in Culture
**4a**	Non-toxic	normal morphology for fibroblasts—elongated cells, single granular cells in 1 of 10 assessed fields of view
**5a**	Non-toxic	normal morphology for fibroblasts—elongated cells, single granular cells in 1 of 10 assessed fields of view
**6a**	Non-toxic	normal morphology for fibroblasts—elongated cells, single granular cells in 1 of 10 assessed fields of view
**7a**	Non-toxic	normal morphology for fibroblasts—elongated cells, single granular cells in 1 of 10 assessed fields of view
**8a**	35.60 (4.46)	many granules, cells shrunken, cell lysis was observed
**9a**	Non-toxic	normal morphology for fibroblasts—elongated cells, single granular cells in 1 of 10 assessed fields of view
**4b**	Non-toxic	normal morphology for fibroblasts—elongated cells, single granular cells in 1 of 10 assessed fields of view
**5b**	200.00 (7.05)	granularities were observed in 3–5 fields of view from 10 analyzed fields, cells with an elongated shape characteristic of fibroblasts
**6b**	156.25 (6.70)	granularities were observed in 3–5 fields of view from 10 analyzed fields, cells with an elongated shape characteristic of fibroblasts
**7b**	Non-toxic	normal morphology for fibroblasts—elongated cells, single granular cells in 1 of 10 assessed fields of view
**8b**	22.72 (2.25)	many granules, cells shrunken, cell lysis was observed
**9b**	30.80 (3.04)	many granules, cells shrunken, cell lysis was observed
**4c**	Non-toxic	normal morphology for fibroblasts—elongated cells, single granular cells in 1 of 10 assessed fields of view
**5c**	166.67 (3.35)	granularities were observed in 3–5 fields of view from 10 analyzed fields, cells with an elongated shape characteristic of fibroblasts
**6c**	103.89 (2.75)	granularities were observed in 3–5 fields of view from 10 analyzed fields, cells with an elongated shape characteristic of fibroblasts
**7c**	Non-toxic	normal morphology for fibroblasts—elongated cells, single granular cells in 1 of 10 assessed fields of view
**8c**	18.97 (1.84)	many granules, cells shrunken, cell lysis was observed
**9c**	28.56 (4.59)	many granules, cells shrunken, cell lysis was observed

Note: Cytotoxic compounds which were excluded from further investigations are marked in red.

**Table 2:** The IC_50_ [µM] values determined for COX-1 and COX-2.

Compound	IC_50_ [µM] (SD)		COX-2/COX-1 Selectivity Ratio
	COX-1	COX-2	
**4a**	NA	45.24 (0.018)	-
**5a**	95.75 (0.1)	48.24 (0.04)	0.50
**6a**	NA	43.85 (0.035)	-
**7a**	70.96 (0.2)	48.48 (0.037)	0.68
**9a**	NA	42.64 (0.015)	-
**4b**	79.47 (0.06)	47.83 (0.039)	0.60
**5b**	NA	NA	-
**6b**	NA	NA	-
**7b**	86.30 (0.005)	49.79 (0.001)	0.58
**4c**	NA	48.50 (0.027)	-
**5c**	NA	NA	-
**6c**	NA	NA	-
**7c**	NA	NA	-
*Meloxicam*	83.7 (0.03)	59.2 (0.06)	0.71
*Celecoxib*	56 (0.1)	0.30 (0.08)	0.005
*Diclofenac*	3.5 (0.04)	16.6 (0.03)	4.74

Note: Data are shown as standard deviation (SD), NA stands for “not applicable”.

**Table 3:** Energies of the interactions of the tested compounds with COX-2 were obtained from the molecular study.

	ΔG° [kJmol^−1^]	ΔE_1_ [kJmol^−1^]	ΔE_2_ [kJmol^−1^]	ΔE_3_ [kJmol^−1^]	K_i_ [μM]
**4a**	−22.57	−33.81	−34.52	0.75	109
**5a**	−15.38	−27.84	−28.67	0.84	2010
**6a**	−23.28	−34.48	−35.44	0.96	83
**7a**	−11.32	−23.83	−23.83	0.00	10,250
**9a**	−21.02	−33.48	−33.41	−0.16	206
**4b**	−20.40	−32.85	−34.36	1.50	226
**5b**	11.60	−2.17	−3.89	1.76	-
**6b**	13.29	−0.83	−0.25	1.08	-
**7b**	−5.93	−19.64	−19.10	−0.84	90,800
**4c**	−8.07	−21.82	−23.24	1.42	38,200
**5c**	29.63	14.67	11.87	2.71	-
**6c**	52.36	36.65	35.44	1.21	-
**7c**	6.18	−8.78	−8.66	−0.12	-
*Meloxicam*	−34.02	−37.74	−37.32	−0.42	1.09
*Celecoxib*	−30.17	−36.40	−36.11	−0.08	5.13
*Diclofenac*	−29.59	−35.82	−30.18	−5.63	6.50

Notes: ΔG°—binding free energy; ΔE_1_—intermolecular interaction energy, which is the sum of van der Waals energy, hydrogen bonding energy, desolvation free energy and electrostatic energy; ΔE_2_—the sum of van der Waals energy, hydrogen bonding energy and desolvation free energy; ΔE_3_—electrostatic energy, K_i_—inhibit constants.

**Table 4:** The Stern–Volmer constant K_sv_ and the quenching rate constant k_q_, the binding constants K_b_ and number of binding sites n and the thermodynamic parameters for the interaction of BSA with studied compounds at different temperatures.

		Quenching		Binding			Thermodynamic		
	T [K]	K_sv_ × 10^5^ [dm^3^mol^−1^]	k_q_ × 10^13^ [dm^3^mol^−1^·s^−1^]	logK_b_	K_b_ × 10^4^ [dm^3^mol^−1^]	n	ΔG° [kJmol^−1^]	ΔH° [kJmol^−1^]	ΔS° [Jmol^−1^K^−1^]
**4a**	297 303 308	0.82 0.56 0.24	0.82 0.56 0.24	4.95 ± 0.09 4.65 ± 0.09 4.35 ± 0.27	8.91 4.47 2.24	1.00 ± 0.02 0.98 ± 0.02 0.99 ± 0.05	−28.20	−95.14	−225.40
**5a**	297 303 308	0.89 0.97 0.80	0.89 0.97 0.80	4.91 ± 0.14 4.74 ± 0.04 4.30 ± 0.07	8.13 5.50 2.00	0.99 ± 0.02 0.96 ± 0.01 0.89 ± 0.01	−28.20	−95.17	−225.50
**6a**	297 303 308	0.51 0.40 0.14	0.51 0.40 0.14	4.69 ± 0.13 4.27 ± 0.14 3.51 ± 0.22	4.90 1.86 0.33	0.99 ± 0.02 0.94 ± 0.02 0.89 ± 0.04	−27.07	−186.69	−537.43
**7a**	297 303 308	0.66 0.27 0.10	0.66 0.27 0.10	4.67 ± 0.07 4.09 ± 0.20 3.26 ± 0.21	4.68 1.23 0.99	0.97 ± 0.01 0.94 ± 0.03 0.87 ± 0.04	−26.89	−222.14	−657.43
**9a**	297 303 308	1.71 0.48 0.20	1.71 0.48 0.20	4.91 ± 0.05 4.52 ± 0.09 4.23 ± 0.18	4.90 1.86 0.33	0.94 ± 0.01 0.97 ± 0.02 0.98 ± 0.03	−27.89	−108.40	−271.08
**4b**	297 303 308	1.76 0.97 0.42	1.76 0.97 0.42	4.82 ± 0.20 4.47 ± 0.22 3.82 ± 0.20	6.61 2.95 0.61	0.92 ± 0.03 0.91 ± 0.03 0.86 ± 0.02	−27.75	−156.84	−434.67
**5b**	297 303 308	1.72 0.97 0.25	1.72 0.97 0.25	4.76 ± 0.12 4.22 ± 0.13 3.94 ± 0.17	5.75 1.66 0.87	0.92 ± 0.02 0.93 ± 0.02 0.92 ± 0.03	−26.92	−131.53	−352.20
**6b**	297 303 308	1.78 0.52 0.48	1.78 0.52 0.48	4.91 ± 0.15 4.32 ± 0.12 3.86 ± 0.06	8.13 2.09 0.72	0.95 ± 0.02 0.93 ± 0.02 0.90 ± 0.01	−27.90	−167.25	−469.18
**7b**	297 303 308	3.07 1.95 0.78	3.07 1.95 0.78	4.97 ± 0.17 4.53 ± 0.16 4.07 ± 0.10	9.33 3.39 1.17	0.91 ± 0.03 0.87 ± 0.02 0.86 ± 0.02	−28.36	−142.60	−384.65
**4c**	297 303 308	0.59 0.24 0.37	0.59 0.24 0.37	4.82 ± 0.19 4.37 ± 0.40 3.97 ± 0.12	5.61 2.34 0.93	1.01 ± 0.03 0.99 ± 0.08 0.90 ± 0.02	−27.44	−135.07	−362.40
**5c**	297 303 308	0.81 0.60 0.36	0.81 0.60 0.36	4.97 ± 0.18 4.51 ± 0.10 4.11 ± 0.13	9.33 3.24 1.29	1.01 ± 0.03 0.95 ± 0.02 0.92 ± 0.02	−28.29	−136.72	−365.09
**6c**	297 303 308	2.68 0.76 0.31	2.68 0.76 0.31	4.98 ± 0.12 4.61 ± 0.03 4.28 ± 0.12	9.55 4.07 1.91	0.92 ± 0.02 0.95 ± 0.01 0.96 ± 0.02	−28.34	−111.23	−279.08
**7c**	297 303 308	2.37 1.03 0.41	2.37 1.03 0.41	4.97 ± 0.20 4.53 ± 0.10 3.94 ± 0.18	9.33 3.39 0.87	0.93 ± 0.03 0.92 ± 0.02 0.88 ± 0.03	−28.48	−162.47	−451.14

**Table 5:** The values of the calculated α-helix(%) for BSA with the absence and presence of all analyzed compounds.

BSA/Analyzed Compound Molar Ratio	α-Helix [%]												
	4a	5a	6a	7a	9a	4b	5b	6b	7b	4c	5c	6c	7c
**1:0**	51.83	52.15	51.93	51.59	51.90	51.08	52.80	52.44	52.87	52.70	53.10	53.49	52.77
**1:0.5**	51.35	51.52	51.28	50.49	50.79	51.40	51.05	51.89	51.78	52.64	52.24	52.41	52.07
**1:1**	51.16	50.62	50.74	49.99	50.74	50.48	50.86	49.81	51.75	51.66	52.49	51.81	51.22
**1:5**	49.80	50.26	49.59	48.79	49.76	49.74	51.21	49.15	49.92	51.10	50.36	50.74	50.31
**1:10**	48.09	47.56	47.45	48.87	46.94	47.00	48.53	47.66	46.99	47.45	50.12	48.01	48.19

**Table 6:** Secondary structure of free BSA and BSA-drug complexes at pH = 7.5 calculated from Amide I bond obtained by FT-IR.

Compound	α-Helix [%]	β-Sheet [%]	β-Turn [%]	β-Anti [%]	Random-Coil [%]
Free—BSA	53.8	29.2	6.6	1.6	8.8
					
**4a**—BSA	50.2	16.5	11.8	11.8	9.7
**5a**—BSA	50.4	16.5	8.9	15.4	8.8
**6a**—BSA	50.3	17.8	4.9	19.5	7.5
**7a**—BSA	51.7	17.1	8.5	15.6	7.1
**9a**—BSA	51.8	16.2	5.8	18.4	7.8
					
**4b**—BSA	50.2	14.3	11.7	16.4	7.4
**5b**—BSA	49.7	13.8	13.7	12.5	10.3
**6b**—BSA	49.8	12.4	12.3	13.4	12.1
**7b**—BSA	51.5	14.2	15.6	9.9	8.8
					
**4c**—BSA	50.1	17.3	13.2	8.3	11.1
**5c**—BSA	49.1	6.1	16.4	3.6	24.8
**6c**—BSA	49.9	5.6	18.8	4.1	21.6
**7c**—BSA	48.3	6.6	18.6	4.2	22.3

**Table 7:** The binding constant of the studied compounds with BSA in the presence of site markers *Phenylbutazone* (PHB) and *Ibuprofen* (IBP) at 297 K.

	Site Marker	logK_b_		Site Marker	logK_b_		Site Marker	logK_b_
**4a**	- BSA + IBP BSA + PHP	4.95 ± 0.09 3.50 ± 0.07 4.54 ± 0.05	**4b**	- BSA + IBP BSA + PHP	4.82 ± 0.20 3.55 ± 0.19 4.55 ± 0.07	**4c**	- BSA + IBP BSA + PHP	4.82 ± 0.19 3.30 ± 0.08 4.57 ± 0.12
**5a**	- BSA + IBP BSA + PHP	4.91 ± 0.14 3.60 ± 0.09 4.36 ± 0.07	**5b**	- BSA + IBP BSA + PHP	4.76 ± 0.12 3.45 ± 0.05 4.25 ± 0.13	**5c**	- BSA + IBP BSA + PHP	4.97 ± 0.18 3.27 ± 0.09 4.58 ± 0.05
**6a**	- BSA + IBP BSA + PHP	4.69 ± 0.13 3.40 ± 0.11 4.10 ± 0.09	**6b**	- BSA + IBP BSA + PHP	4.91 ± 0.15 3.60 ± 0.12 4.46 ± 0.15	**6c**	- BSA + IBP BSA + PHP	4.98 ± 0.12 3.68 ± 0.06 4.63 ± 0.05
**7a**	- BSA + IBP BSA + PHP	4.67 ± 0.07 3.37 ± 0.09 4.20 ± 0.11	**7b**	- BSA + IBP BSA + PHP	4.97 ± 0.17 3.20 ± 0.08 4.39 ± 0.07	**7c**	- BSA + IBP BSA + PHP	4.97 ± 0.20 3.50 ± 0.14 4.58 ± 0.09
**9a**	- BSA + IBP BSA + PHP	4.91 ± 0.05 3.50 ± 0.09 4.55 ± 0.10						

**Table 8:** Energies of the binding complexes BSA with studied compounds obtained from molecular docking.

	Binding Site	ΔG° [kJmol^−1^]	ΔE_1_ [kJmol^−1^]	ΔE_2_ [kJmol^−1^]	ΔE_3_ [kJmol^−1^]
**4a**	site I site II(m) site II(l)	−31.02 −40.08 −27.42	−42.22 −51.33 −38.66	−37.66 −46.73 −39.88	−4.56 −4.60 1.21
**5a**	site I site II(m) site II(l)	−29.93 −38.22 −28.88	−42.38 −50.74 −41.38	−39.00 −46.89 −42.38	−3.38 −3.85 1.00
**6a**	site I site II(m) site II(l)	−30.18 −38.03 −31.30	−42.79 −49.28 −42.55	−38.67 −46.48 −43.72	−4.10 −2.80 1.17
**7a**	site I site II(m) site II(l)	−30.51 −35.69 −28.55	−43.01 −48.20 −41.04	−42.93 −47.36 −40.38	−0.04 −0.84 −0.67
**9a**	site I site II(m) site II(l)	−31.06 −35.44 −25.16	−43.55 −47.95 −37.62	−43.35 −47.19 −37.12	−0.21 −0.76 −0.50
**4b**	site I site II(m) site II(l)	−35.53 −41.17 −25.37	−47.98 −53.62 −37.87	−44.02 −50.20 −38.03	−4.01 −3.43 0.20
**5b**	site I site II(m) site II(l)	−32.02 −39.54 −30.47	−45.73 −53.30 −44.18	−41.05 −50.95 −45.06	−4.68 −2.30 0.84
**6b**	site I site II(m) site II(l)	−33.56 −41.17 −28.30	−46.02 −53.63 −40.76	−41.08 −51.00 −39.67	−4.22 −2.63 −1.08
**7b**	site I site II(m) site II(l)	−29.30 −39.67 −23.57	−43.01 −53.38 −37.28	−42.64 −52.83 −37.57	−0.37 −0.55 0.29
**4c**	site I site II(m) site II(l)	−34.48 −38.79 −28.34	−48.19 −52.50 −45.01	−43.76 −49.87 −43.09	−4.43 −2.63 1.08
**5c**	site I site II(m) site II(l)	−31.51 −42.48 −22.40	−46.48 −57.81 −37.37	−42.18 −54.88 −39.17	−4.26 −2.93 1.80
**6c**	site I site II(m) site II(l)	−31.77 −37.20 −24.03	−45.48 −50.91 −37.75	−42.39 −47.82 −36.12	−3.09 −3.09 −1.63
**7c**	site I site II(m) site II(l)	−27.21 −38.12 −33.89	−42.17 −53.08 −48.86	−41.08 −52.37 −48.91	−1.09 −0.71 0.05

ΔG°—binding free energy; ΔE_1_—intermolecular interaction energy, which is the sum of van der Waals energy, hydrogen bonding energy, desolvation free energy and electrostatic energy; ΔE_2_—the sum of van der Waals energy, hydrogen bonding energy and desolvation free energy; ΔE_3_—electrostatic energy.

**Table 9:** Predicted physicochemical properties of studied compounds using SWISSADME server.

Compound	Physicochemical Properties—Lipinski’s Rule of Five (Ro5)				
	#H-Bond Acceptors	#H-Bond Donors	Log *P_o/w_* (MLOGP)	MW [g/mol]	#Violations
**4a**	5	0	2.88	550.72	1
**5a**	8	0	3.36	618.72	1
**6a**	5	0	3.07	564.75	1
**7a**	6	0	2.99	578.73	2
**9a**	6	0	3.18	592.76	2
**4b**	5	0	3.89	612.79	1
**5b**	8	0	4.35	680.79	2
**6b**	5	0	4.08	626.81	1
**7b**	5	0	3.99	640.80	2
**4c**	6	0	3.58	642.81	2
**5c**	9	0	4.03	710.81	2
**6c**	6	0	3.76	656.84	2
**7c**	7	0	3.68	670.82	2

**Table 10:** Predicted ADME parameters of studied compounds using SWISSADME server.

Compound	Pharmacokinetics			
	GI Absorption	BBB Permeant	P-gp Substrate	Water Solubility
**4a**	High	No	Yes	Moderately soluble
**5a**	High	No	Yes	Poorly soluble
**6a**	High	No	Yes	Moderately soluble
**7a**	High	No	Yes	Poorly soluble
**9a**	High	No	Yes	Poorly soluble
**4b**	High	No	Yes	Poorly soluble
**5b**	Low	No	Yes	Poorly soluble
**6b**	High	No	Yes	Poorly soluble
**7b**	Low	No	Yes	Poorly soluble
**4c**	High	No	Yes	Poorly soluble
**5c**	Low	No	Yes	Poorly soluble
**6c**	High	No	Yes	Poorly soluble
**7c**	Low	No	Yes	Poorly soluble

**Table 11:** Predicted drug-likeness properties of studied compounds using SWISSADME server.

Compound	Drug-Likeness			
	Lipinski	Veber	Bioavailability Score	TPSA [Å^2^]
**4a**	Yes, 1 violation	Yes	0.55	110.37
**5a**	Yes, 1 violation	Yes	0.55	110.37
**6a**	Yes, 1 violation	Yes	0.55	110.37
**7a**	No, 2 violations	No, 1 violation	0.17	128.61
**9a**	No, 2 violations	No, 1 violation	0.17	128.61
**4b**	Yes, 1 violation	Yes	0.55	110.37
**5b**	No, 2 violations	No, 1 violation	0.17	110.37
**6b**	Yes, 1 violation	Yes	0.55	110.37
**7b**	No, 2 violations	No, 1 violation	0.17	128.61
**4c**	No, 2 violations	No, 1 violation	0.17	119.60
**5c**	No, 2 violations	No, 1 violation	0.17	119.60
**6c**	No, 2 violations	No, 1 violation	0.17	119.60
**7c**	No, 2 violations	No, 1 violation	0.17	137.84

## Data Availability

Calculations have been carried out in Wroclaw Centre for Networking and Supercomputing (http://www.wcss.wroc.pl, accessed on 1 May 2021).

## Supplementary Materials

The following are available online at https://www.mdpi.com/article/10.3390/ijms222011235/s1: The structures of all reported compounds **2a-c-9a-c** (Table S1), the ^1^H and ^13^C NMR (Table S2), ESI-MS (Table S3), FT-IR (Table S4), spectra of reported derivatives. Molecular formula strings (CSV) (Table S5). BSA binding interactions—CD spectra (Table S6), FT-IR spectra (Tables S7 and S8).

## Abbreviations

ADME, Absorption Distribution Metabolism Excretion; Arg, Arginine; ATR, Attenuated Total Reflectance; BBB. Blood Brain Barrier, BSA, Bovine Serum Albumin; CD, Circular Dichroism; COX, Cyclooxygenase; DCF-DA, Dichlorofluorescin Diacetate; DMEM, Dulbecco’s Modified Eagle’s Medium; DMSO, Dimethyl Sulfoxide; ELISA, Enzyme-Linked Immunosorbent Assay; ESI-MS, Electrospray Ionization Mass Spectrometry; FBS, Fetal Bovine Serum; FT-IR, Fourier Transform Infrared; GI, Gastrointestinal; HSA, Human Serum Albumine; IBP, *Ibuprofen*; IC, Inhibitory Concentration; Leu, Leucine; LPS, Lipopolysaccharide; MPO, Myeloperoxidase; MTT 3-(4,5-dimethylthiazol-2-yl)-2,5-diphenyltetrazolium bromide; MW, Molecular Weight; NA, Not Applicable; NHDF, Normal Human Dermal Fibroblasts; NMR, Nuclear Magnetic Resonance; NO, Nitric Oxide; NSAIDs, Non-Steroidal Anti-Inflammatory Drugs; PBS, Phosphate Buffered Saline; PGE_2_, Prostaglandin E_2_; PGs, Prostaglandins; PHB, *Phenylbutazone*; RONS, Reactive Oxygene and Nitrogen Species; Ro5, Rule of Five; ROS, Reactive Oxygen Species; RT, Room Temperature; SAR, Structure-Activity Relationship; SD, Standard Deviation; Ser, Serine; TLC, Thin Layer Chromatography; TMS, Tetramethylsilane; TPSA, Topological Polar Surface Area; Trp, Tryptophan; Tyr, Tyrosine.
