# Supplementary material for: New N-Substituted-1,2,4-triazole Derivatives of Pyrrolo[3,4-d]pyridazinone with Significant Anti-Inflammatory Activity—Design, Synthesis and Complementary In Vitro, Computational and Spectroscopic Studies
Source: Int J Mol Sci. 2021 Oct 18;22(20):11235. doi: 10.3390/ijms222011235 (PMC8540742; doi:10.3390/ijms222011235)
Supplement: Supplementary file 1 [file ijms-22-11235-s001.zip › ijms-1413077-supplementary.pdf]

SUPPLEMENTARY DATA

New *N*-substituted-1,2,4-triazole Derivatives of Pyrrolo[3,4-*d*]pyridazinone with Significant Anti-Inflammatory Activity - Design, Synthesis and Complementary *In Vitro*, Computational and Spectroscopic Studies

Łukasz Szczukowski[1,*], Edward Krzyżak[2], Benita Wiatrak[3], Paulina Jawień[3], Aleksandra Marciniak[2], Aleksandra Kotynia[2], Piotr Świątek[1,*]

[1] *Department of Medicinal Chemistry, Wroclaw Medical University, Borowska 211, 50-556 Wroclaw, Poland*

[2] *Department of Inorganic Chemistry, Wroclaw Medical University, Borowska 211a, 50-556, Wroclaw, Poland*

[3] *Department of Pharmacology, Wroclaw Medical University, Mikulicza-Radeckiego 2, 50-345 Wroclaw, Poland*

Correspondence: lukasz.szczukowski@umed.wroc.pl; piotr.swiatek@umed.wroc.pl

Table of contents

Table S1 - Structures of new compounds S2

Table S2 - NMR spectra of new compounds S4

Table S3 - Mass spectra of new compounds S12

Table S4 – FT-IR spectra of new compounds S23

Table S5 – Molecular formula strings (CSV) of reported compounds S32

Table S6 – CD spectra of BSA in the absence and presence of all analyzed compounds S33

Table S7 - FT-IR spectra - The curve-fitted Amide I region S35

Table S8 - FT-IR spectra – The changes in Amide I (1650cm^{-1}) and Amide II (1545cm^{-1}) band contribution by BSA interaction with analyzed compounds. S37

Table S1 - Structures of new compounds

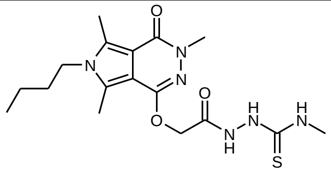	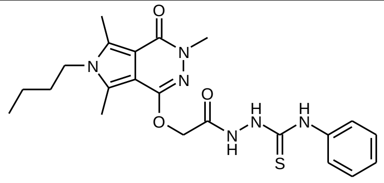	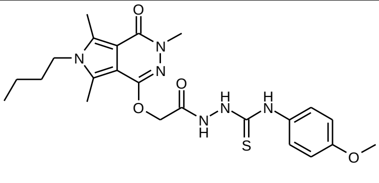
**2a**	**2b**	**2c**
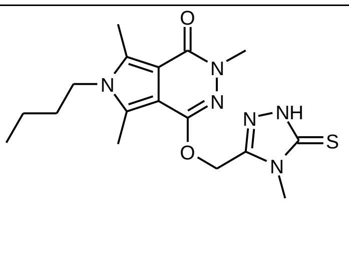	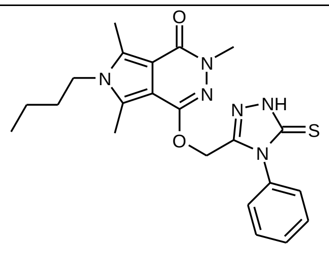	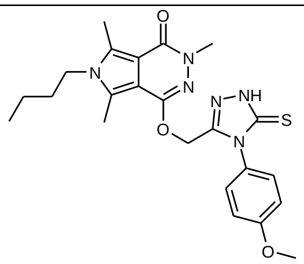
**3a**	**3b**	**3c**
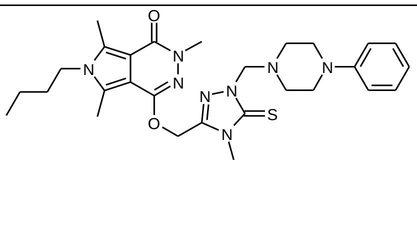	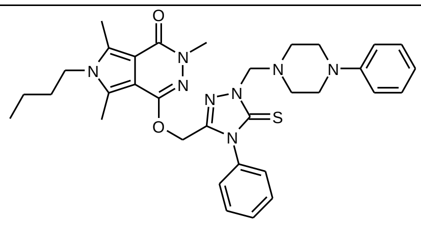	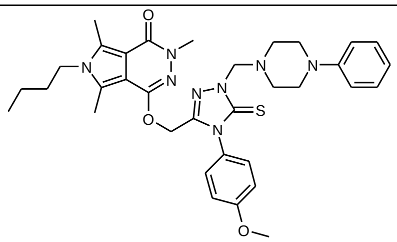
**4a**	**4b**	**4c**
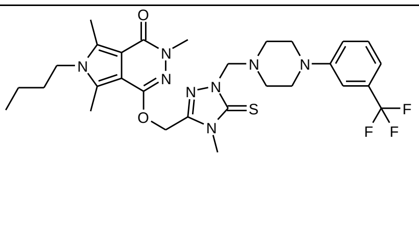	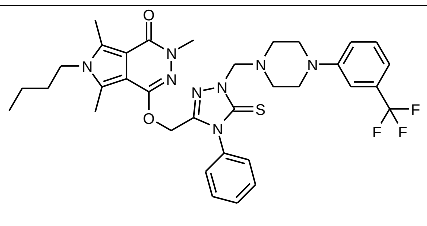	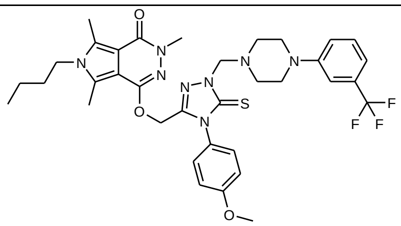
**5a**	**5b**	**5c**
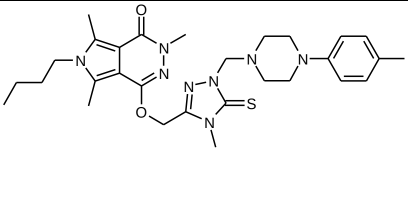	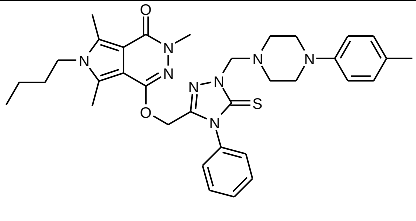	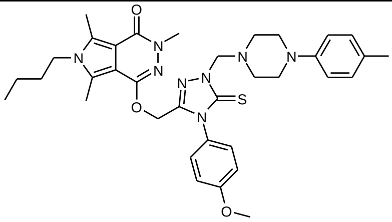
**6a**	**6b**	**6c**
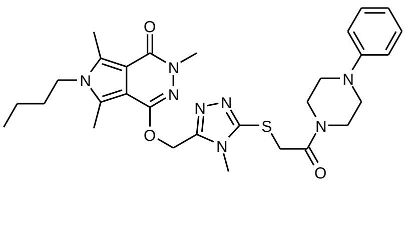	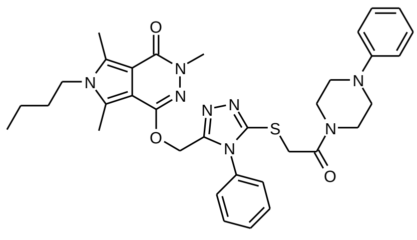	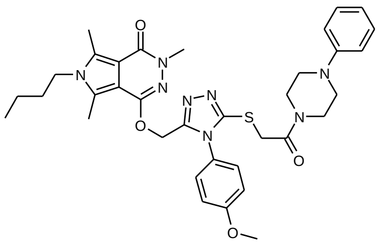
**7a**	**7b**	**7c**

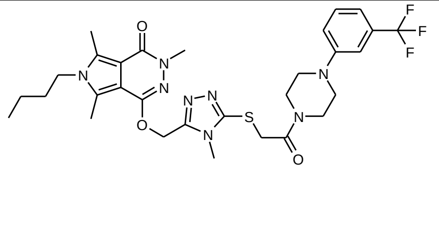	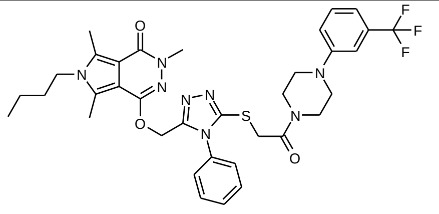	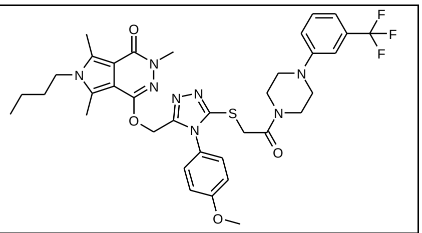
**8a**	**8b**	**8c**
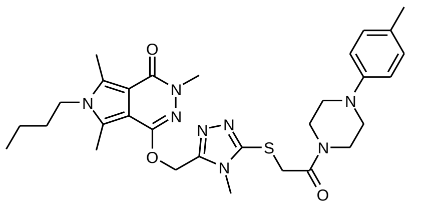	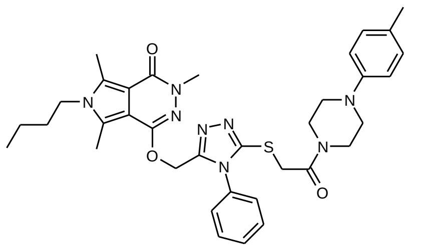	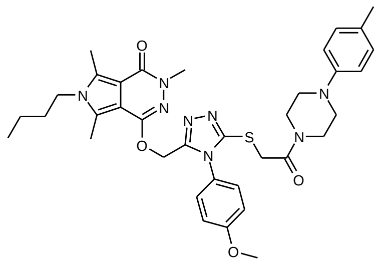
**9a**	**9b**	**9c**

Table S2 - NMR spectra of new compounds

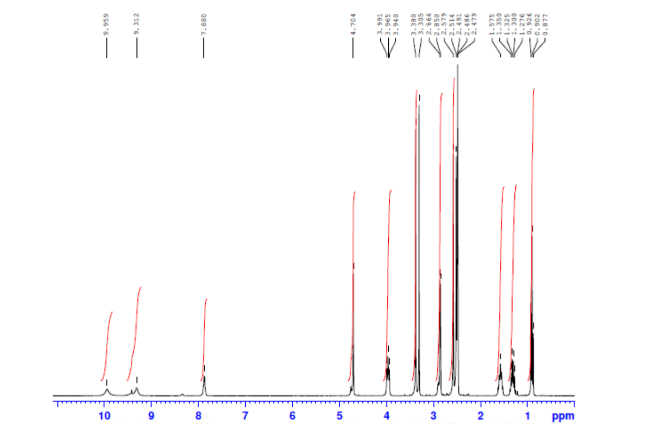	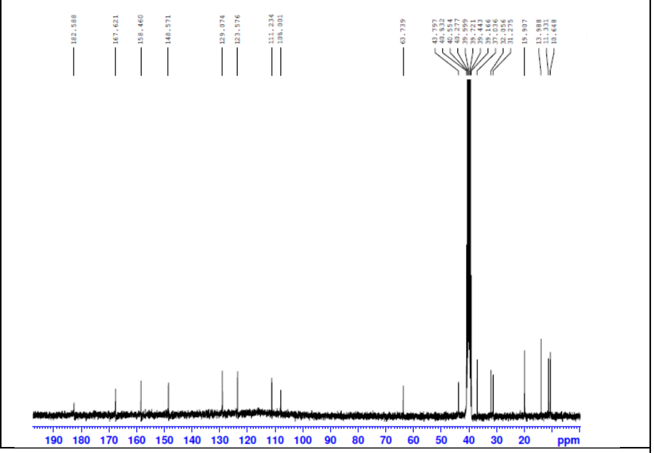
**2a**	
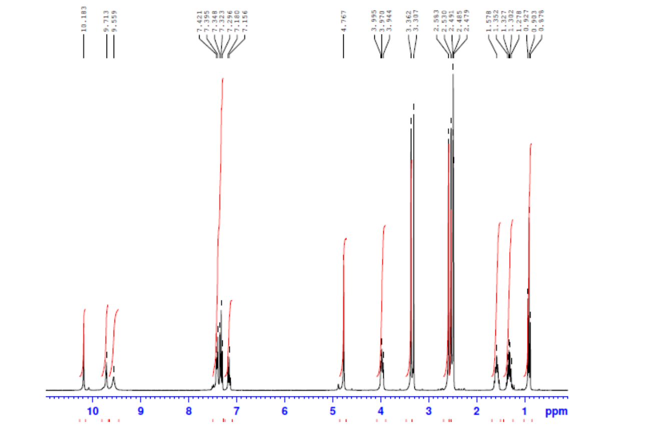	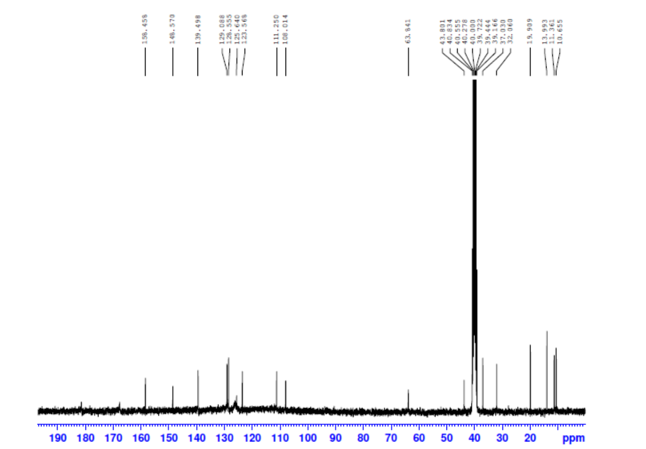
**2b**	
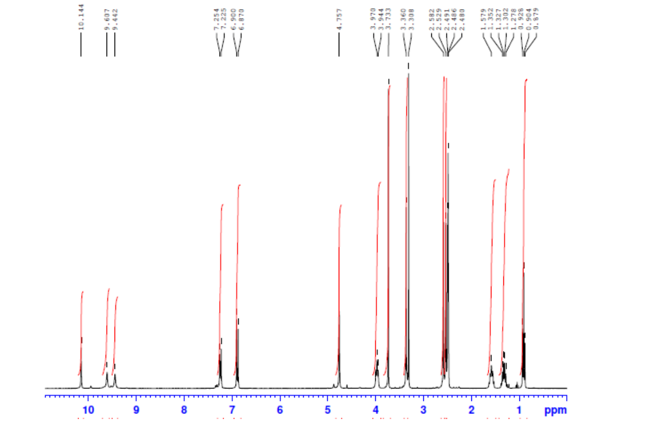	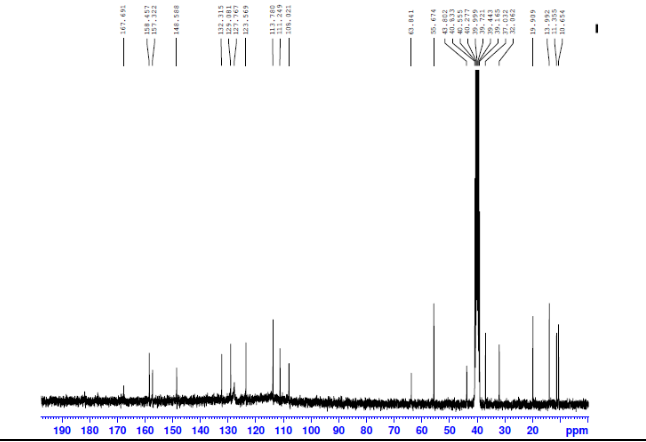
**2c**	

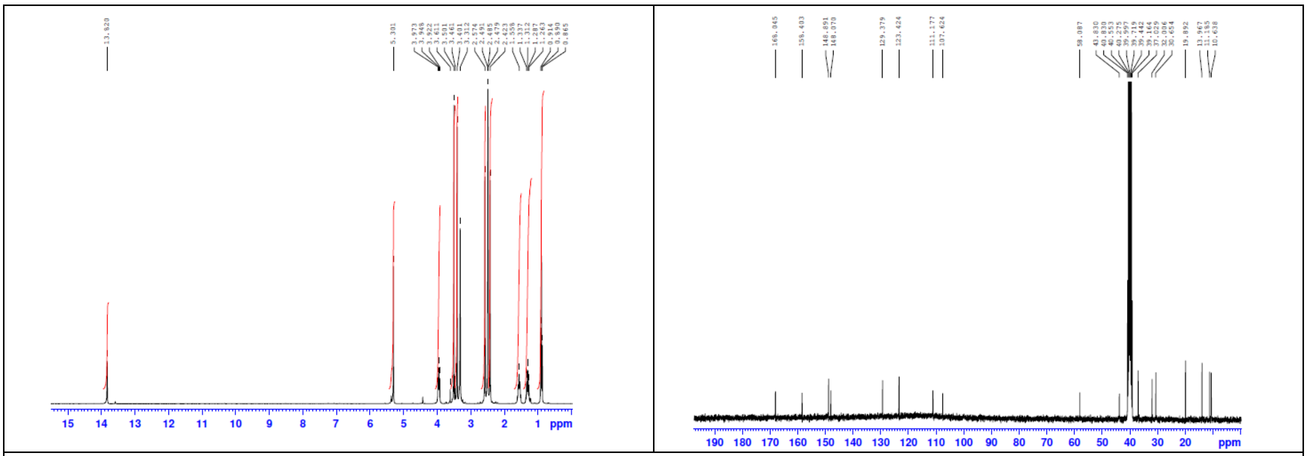

3a

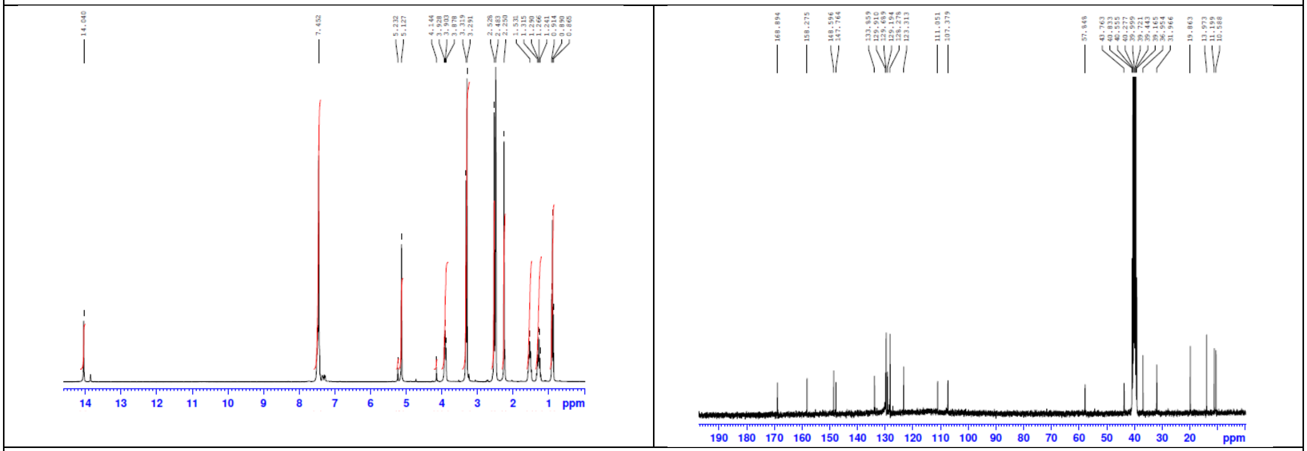

3b

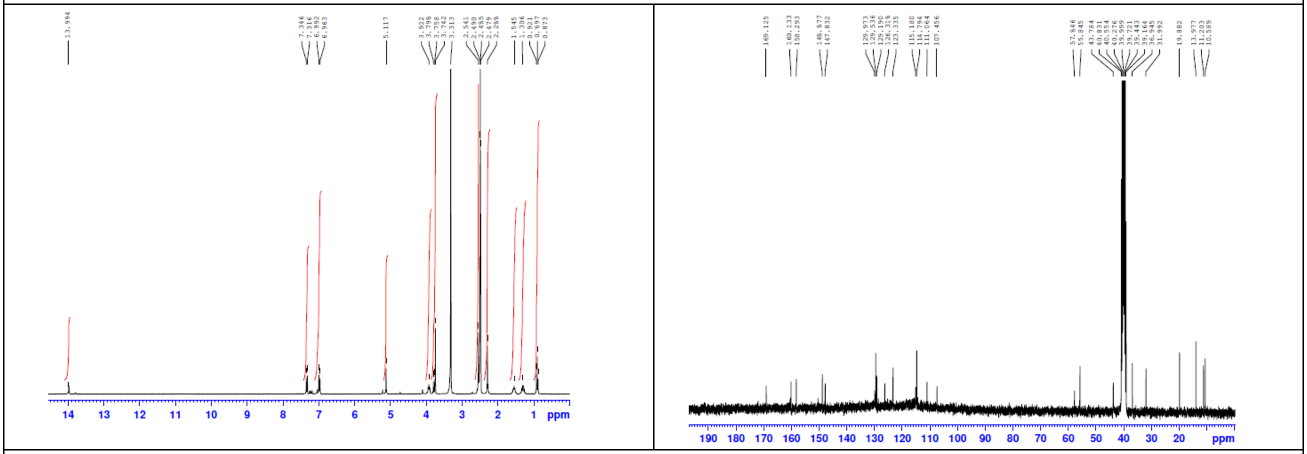

3c

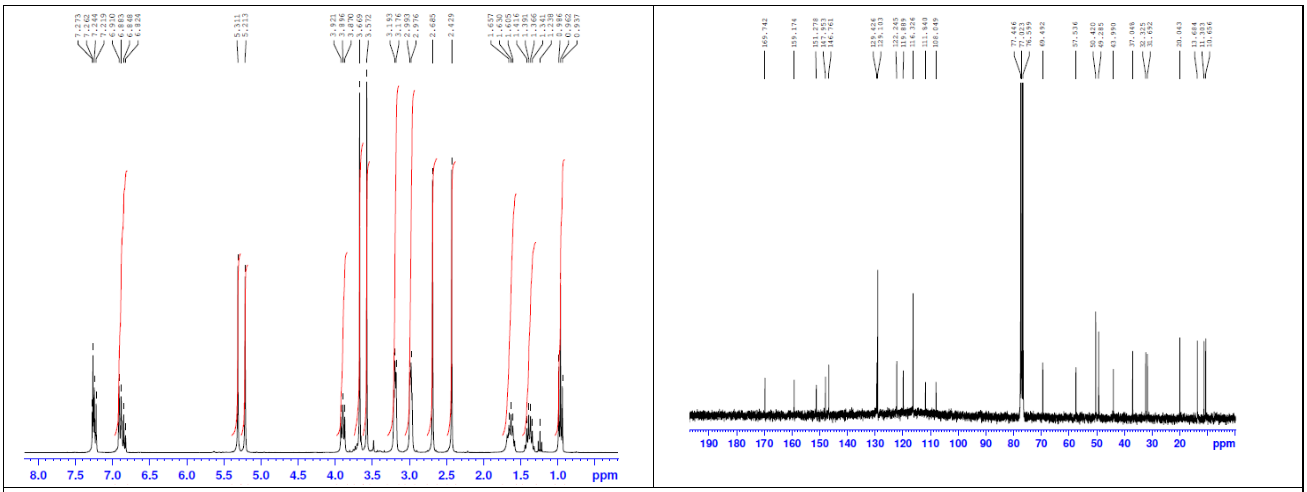
8.0 7.5 7.0 6.5 6.0 5.5 5.0 4.5 4.0 3.5 3.0 2.5 2.0 1.5 1.0 ppm
190 180 170 160 150 140 130 120 110 100 90 80 70 60 50 40 30 20 ppm

4a

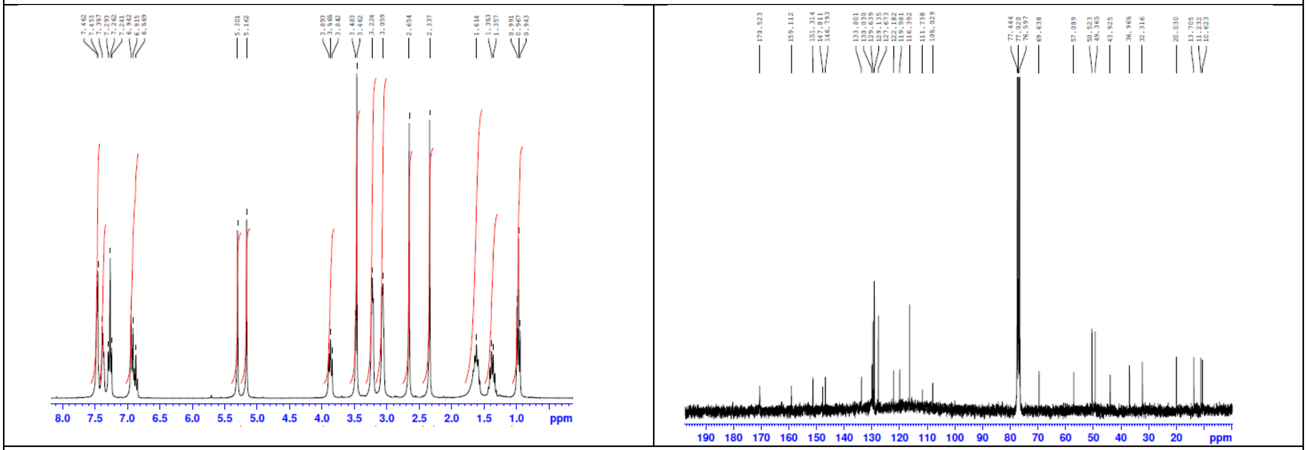
8.0 7.5 7.0 6.5 6.0 5.5 5.0 4.5 4.0 3.5 3.0 2.5 2.0 1.5 1.0 ppm
190 180 170 160 150 140 130 120 110 100 90 80 70 60 50 40 30 20 ppm

4b

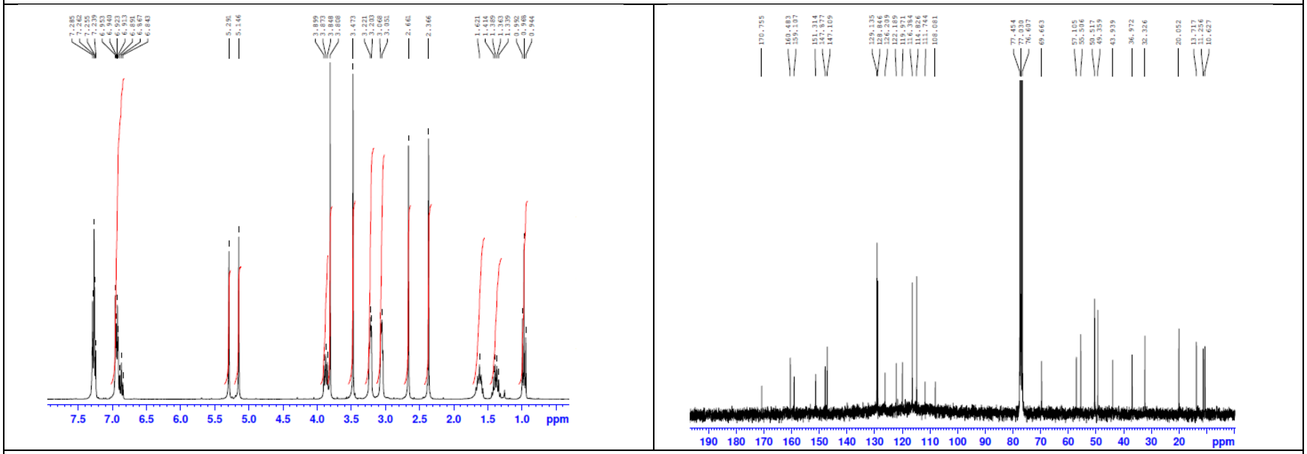
7.5 7.0 6.5 6.0 5.5 5.0 4.5 4.0 3.5 3.0 2.5 2.0 1.5 1.0 ppm
190 180 170 160 150 140 130 120 110 100 90 80 70 60 50 40 30 20 ppm

4c

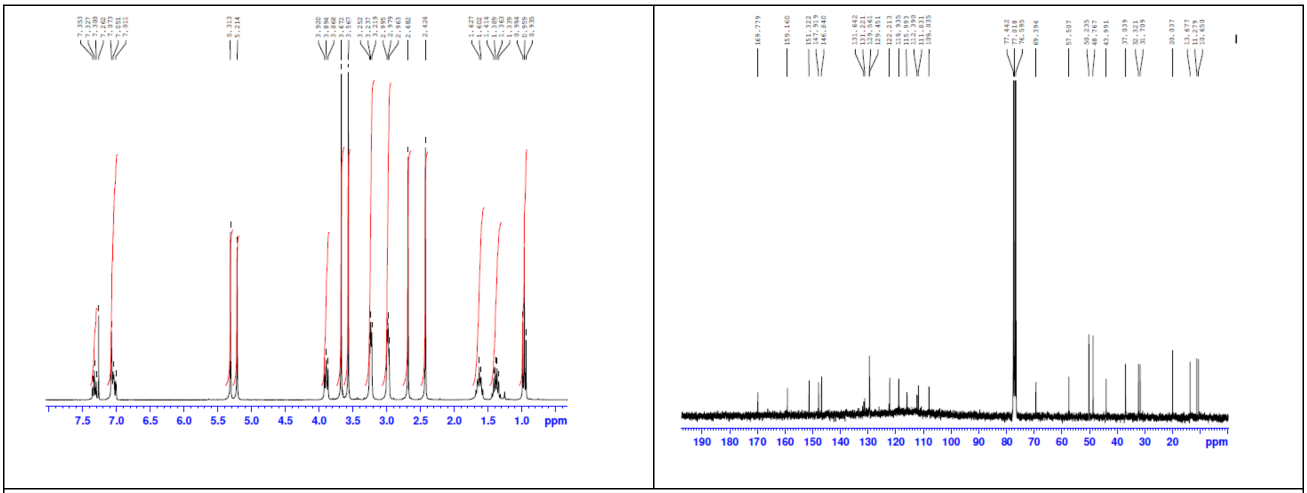

5a

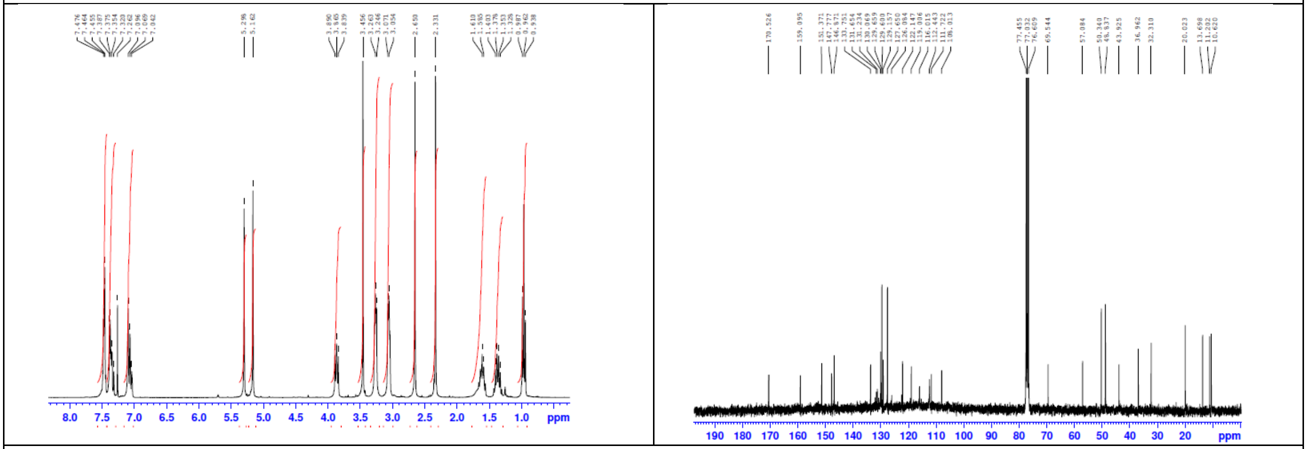

5b

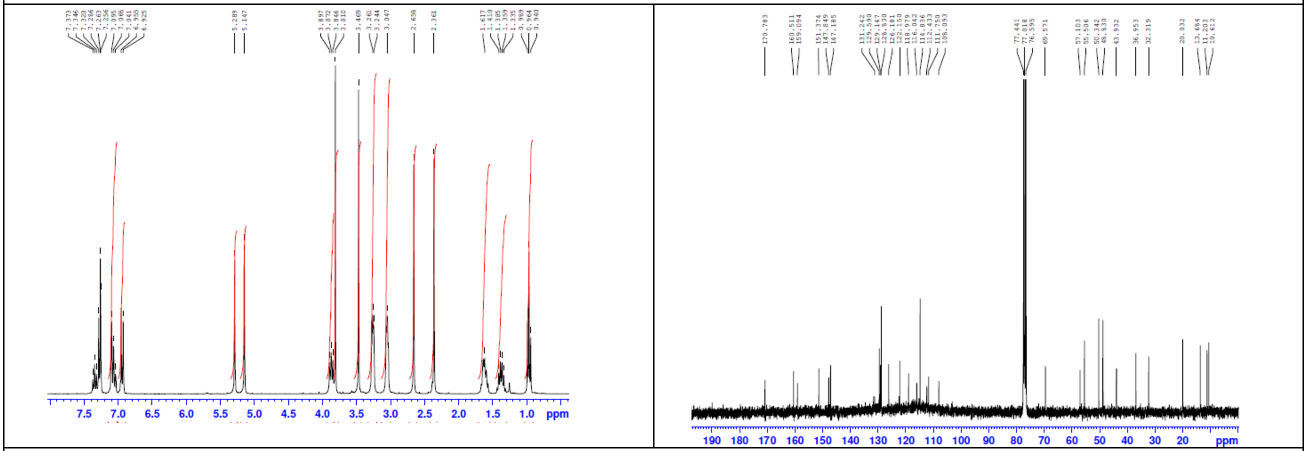

5c

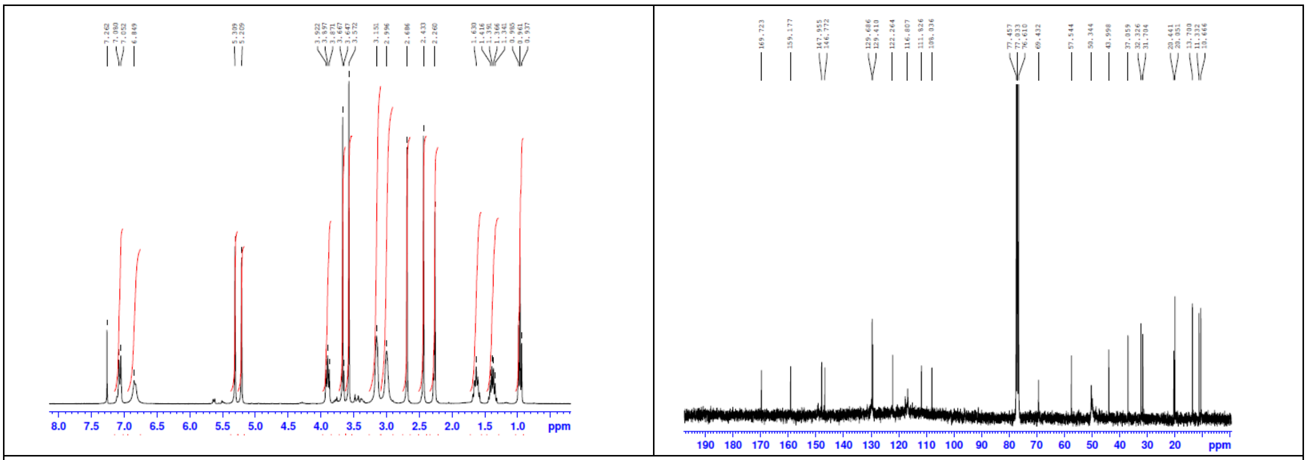

6a

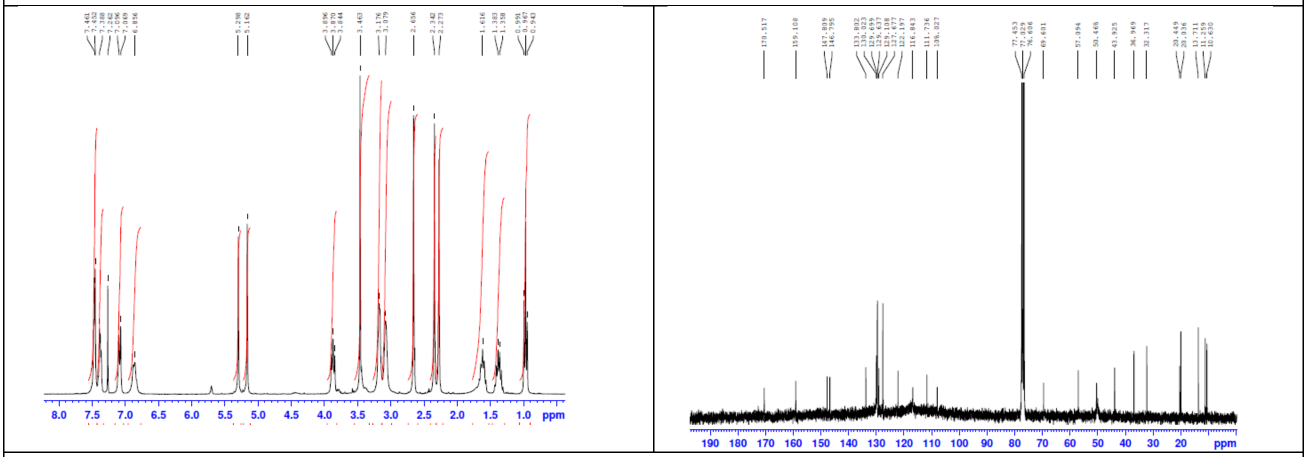

6b

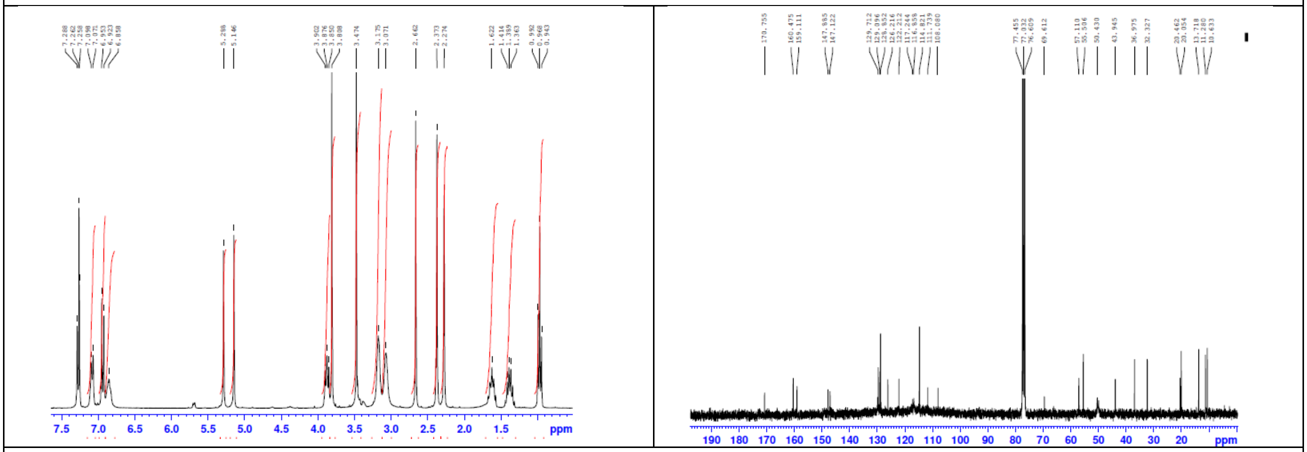

6c

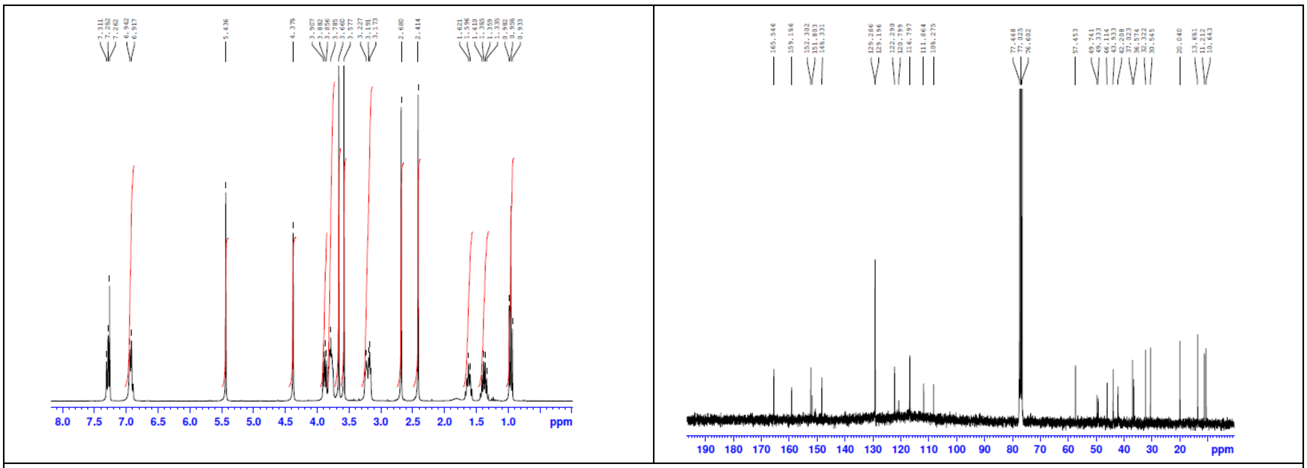

7a

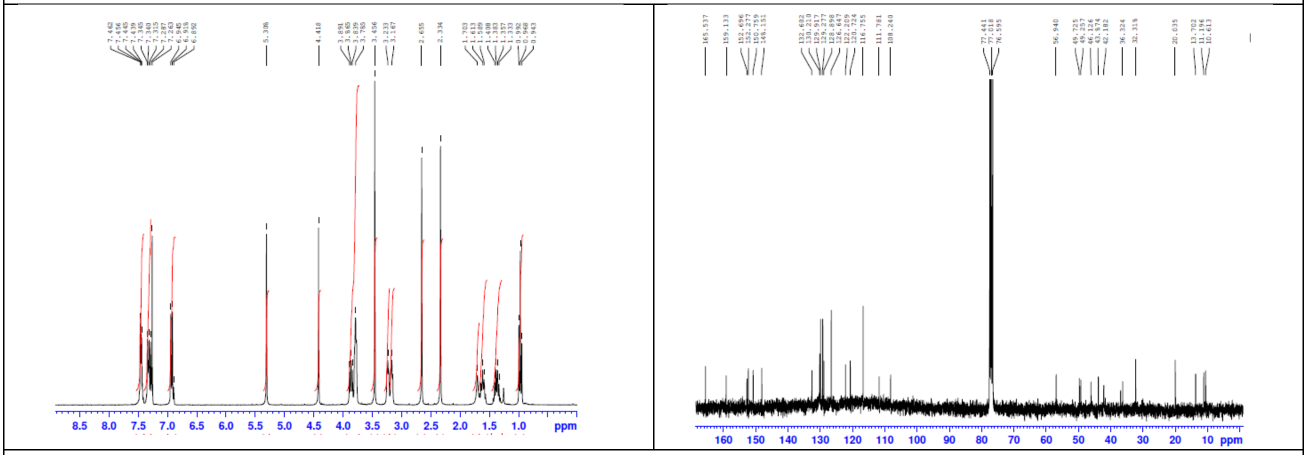

7b

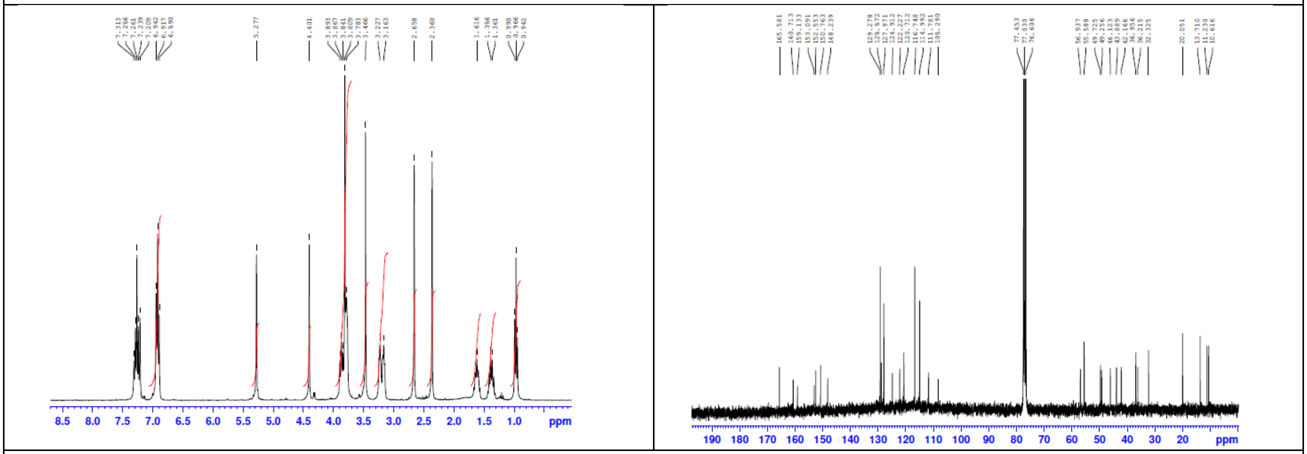

7c

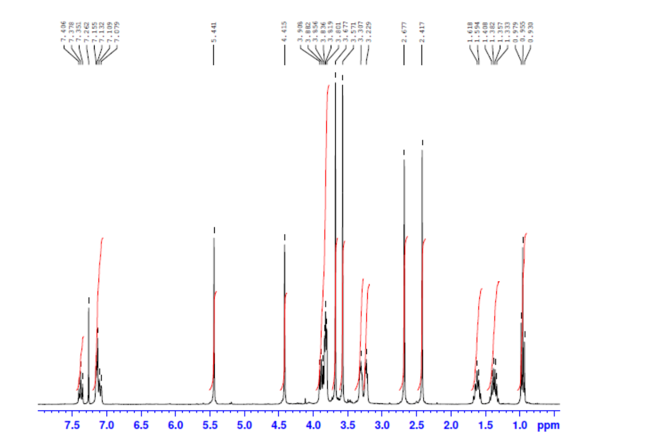
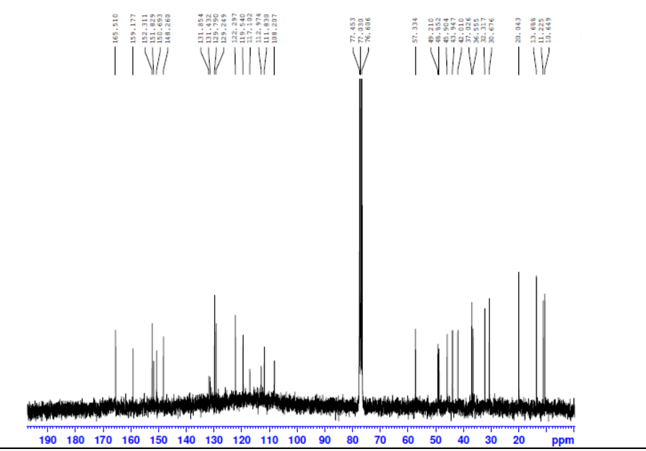

8a

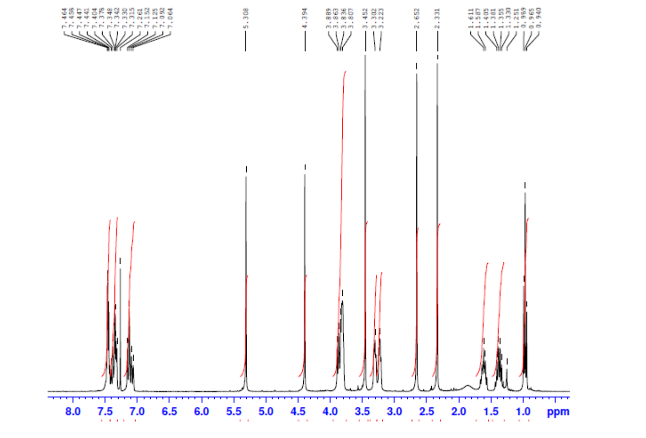
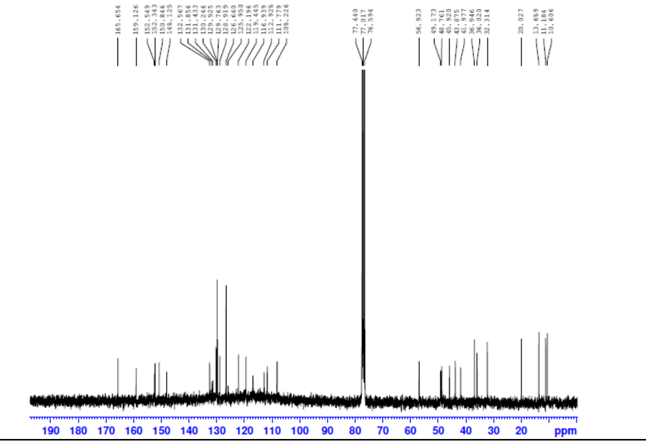

8b

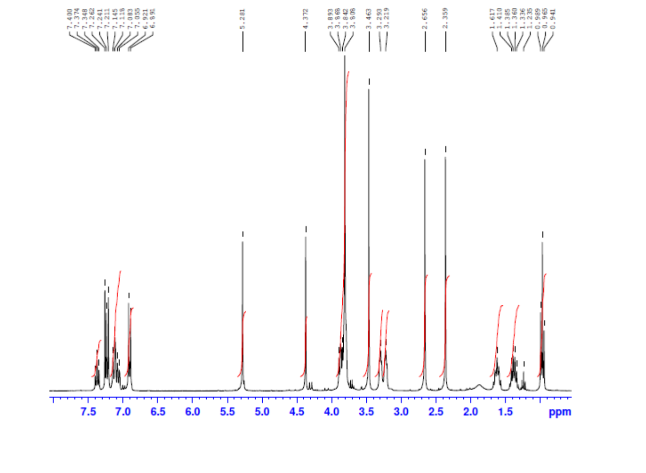
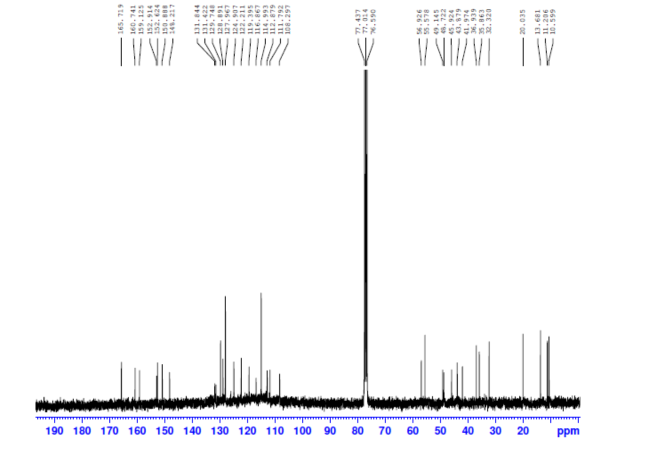

8c

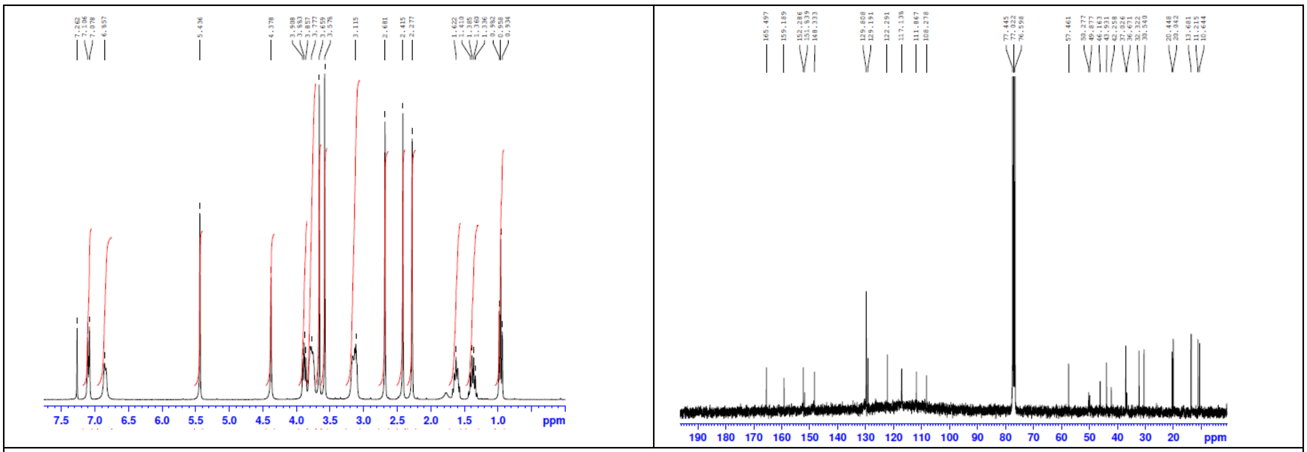

9a

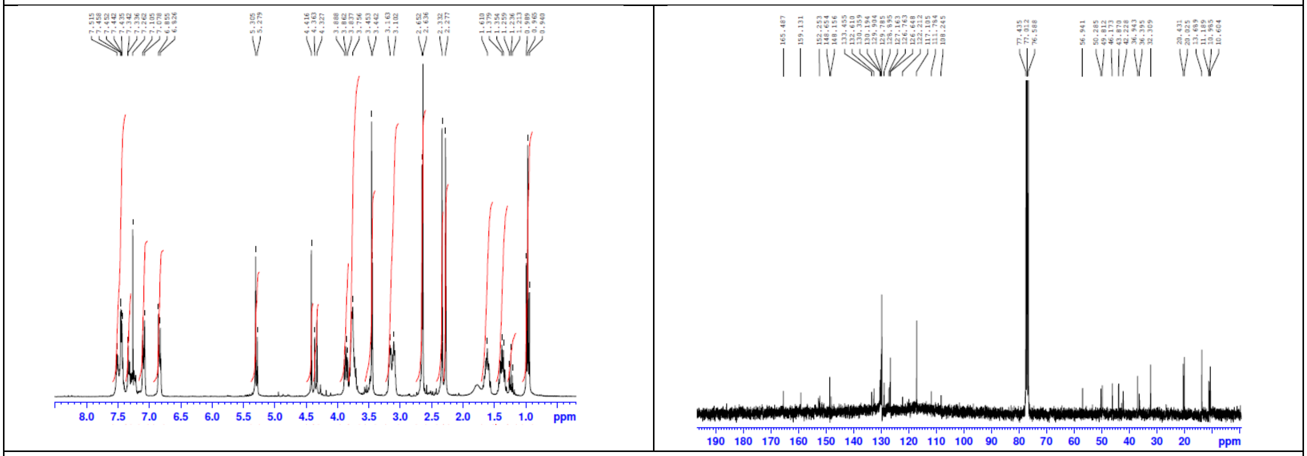

9b

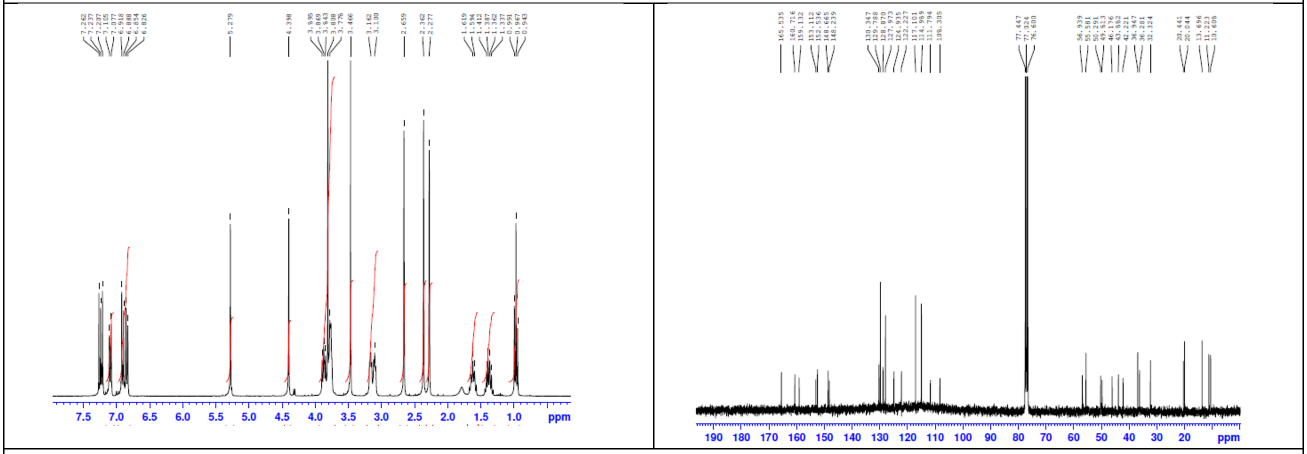

9c

Table S3 - Mass spectra of new compounds

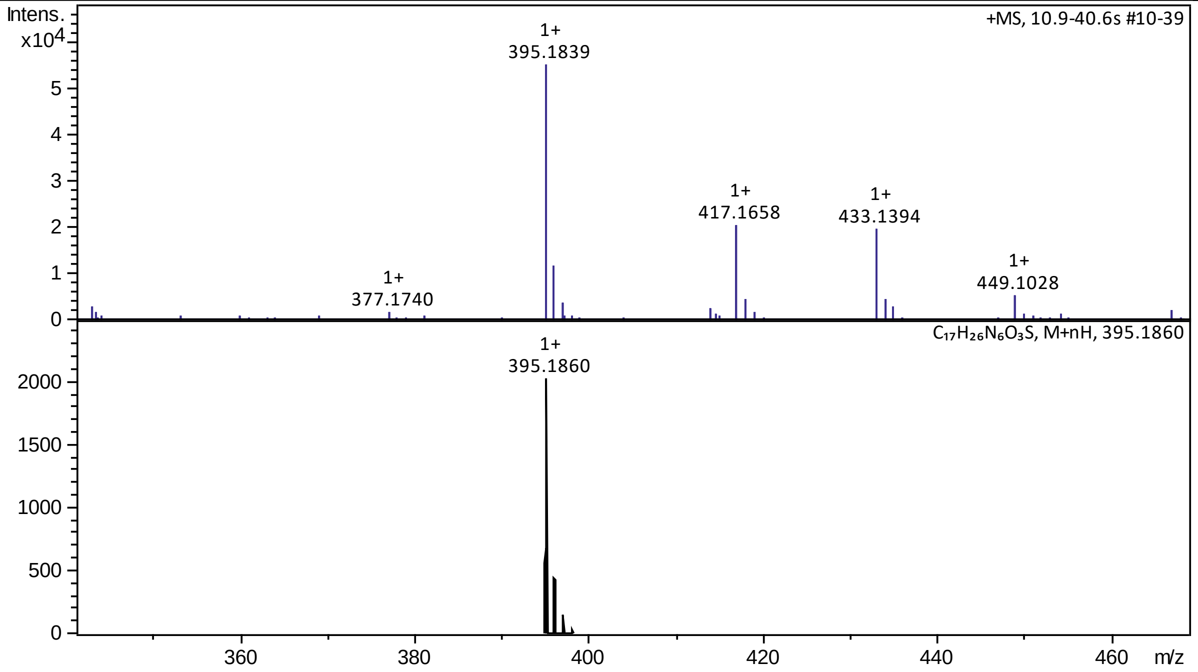

2a

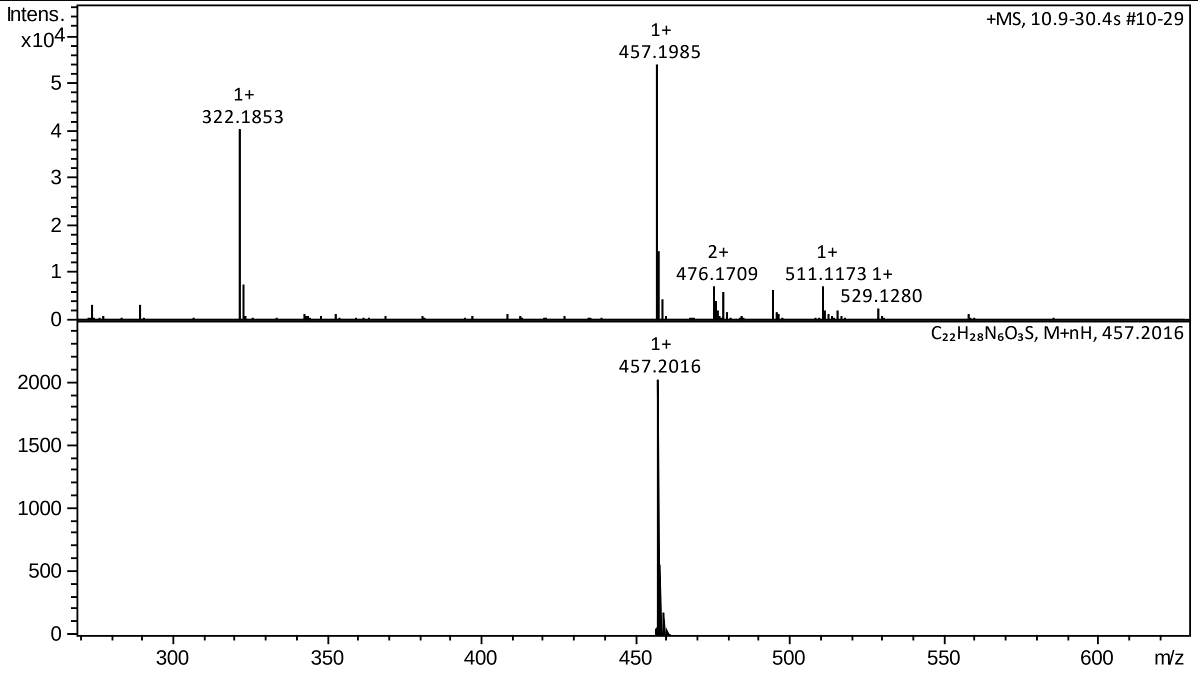

2b

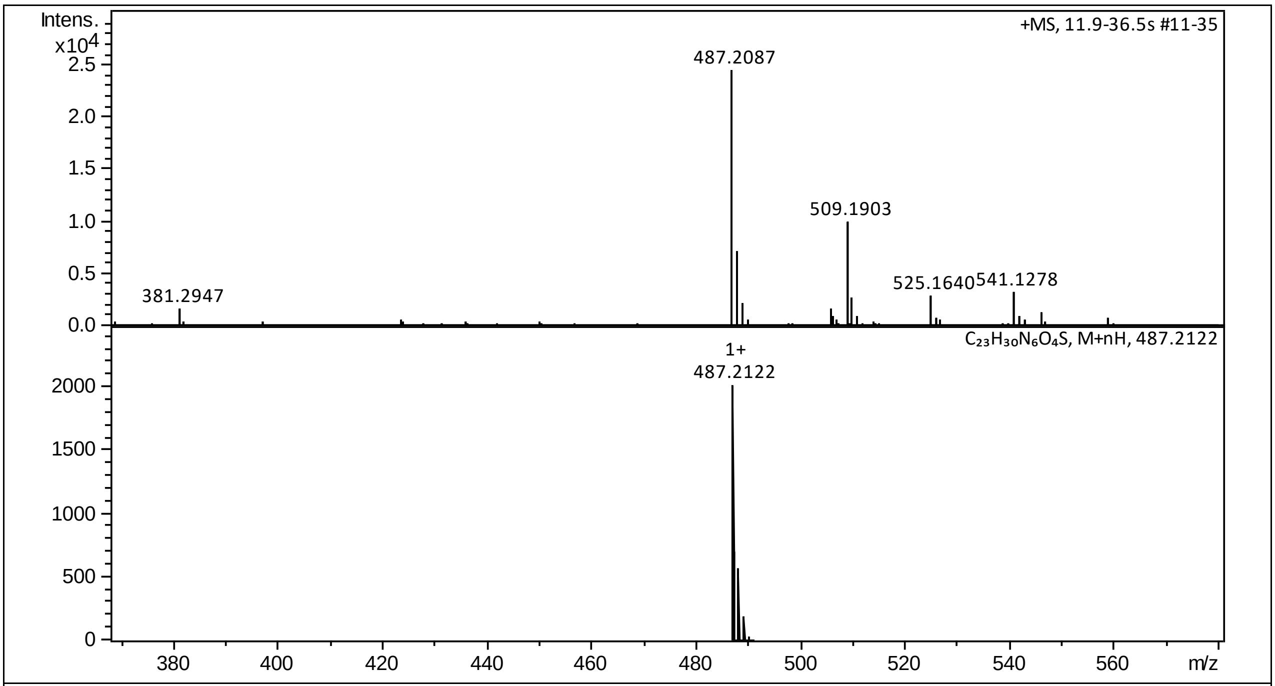
Intens.
x10^4
+MS, 11.9-36.5s #11-35
487.2087
509.1903
525.1640
541.1278
381.2947
C23H30N6O4S, M+nH, 487.2122
1+
487.2122
m/z

2c

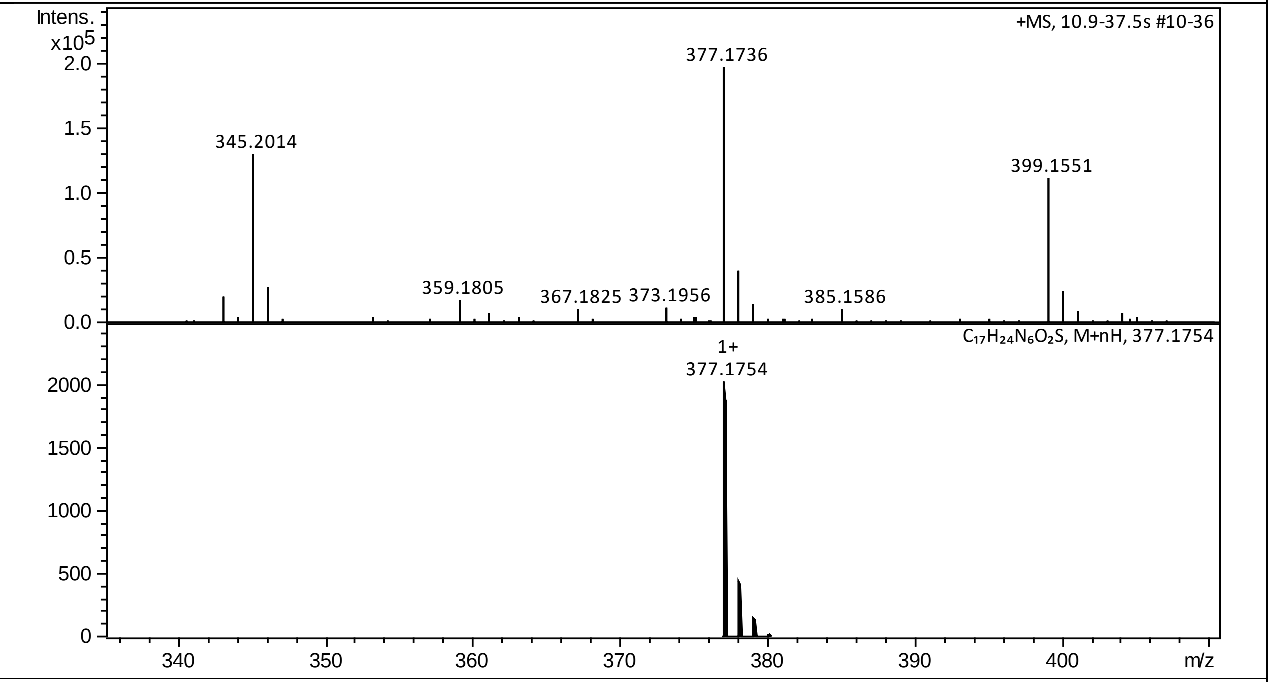
Intens.
x10^5
+MS, 10.9-37.5s #10-36
377.1736
345.2014
399.1551
359.1805
367.1825
373.1956
385.1586
C17H24N6O2S, M+nH, 377.1754
1+
377.1754
m/z

3a

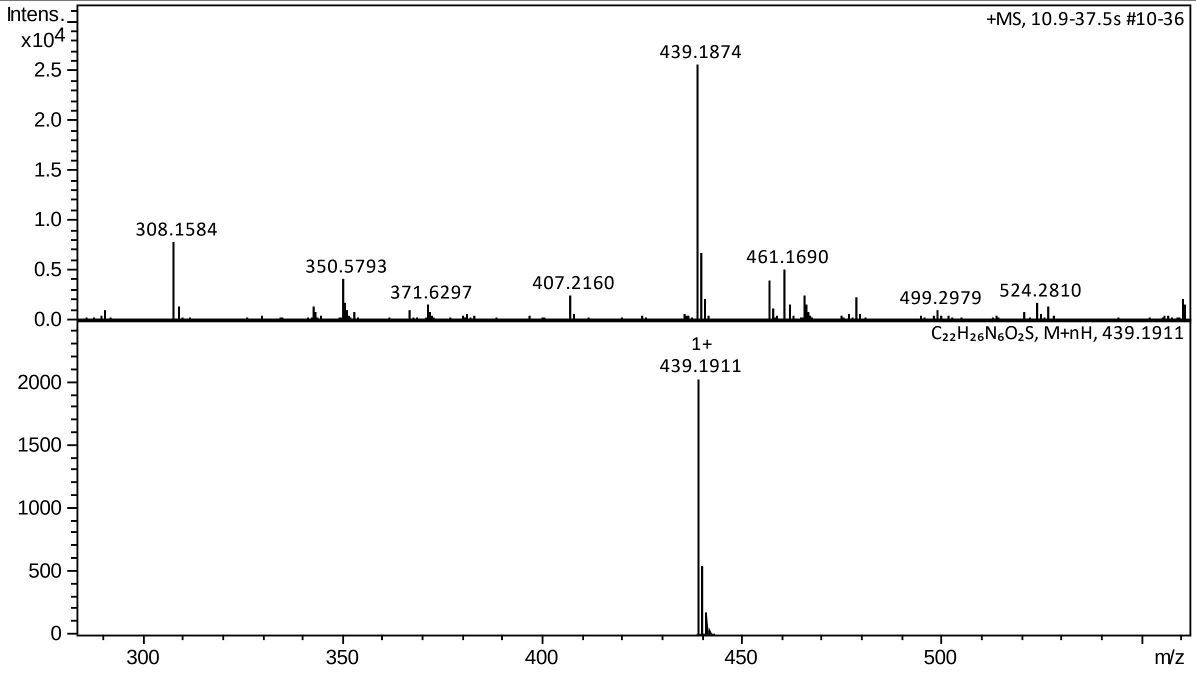
Intens.
x10^4
+MS, 10.9-37.5s #10-36
439.1874
308.1584
350.5793
371.6297
407.2160
461.1690
499.2979
524.2810
C22H26N6O2S, M+nH, 439.1911
1+
439.1911
m/z

3b

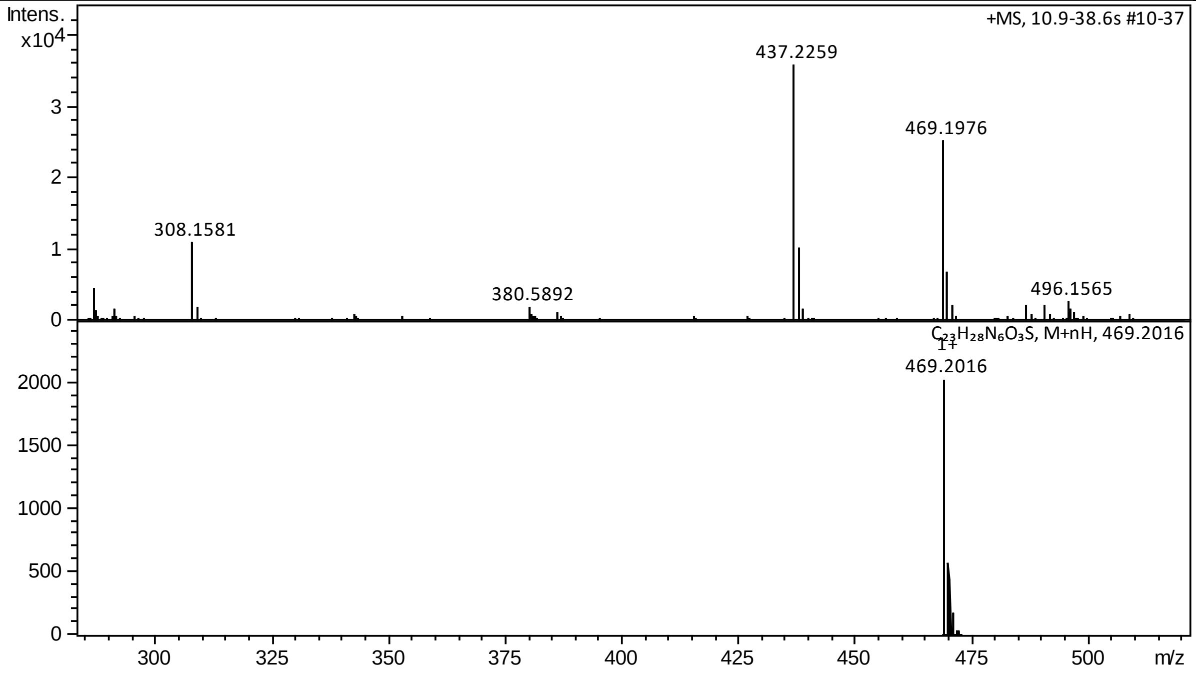
Intens.
x10^4
+MS, 10.9-38.6s #10-37
437.2259
469.1976
308.1581
380.5892
496.1565
C23H28N6O3S, M+nH, 469.2016
1+
469.2016
m/z

3c

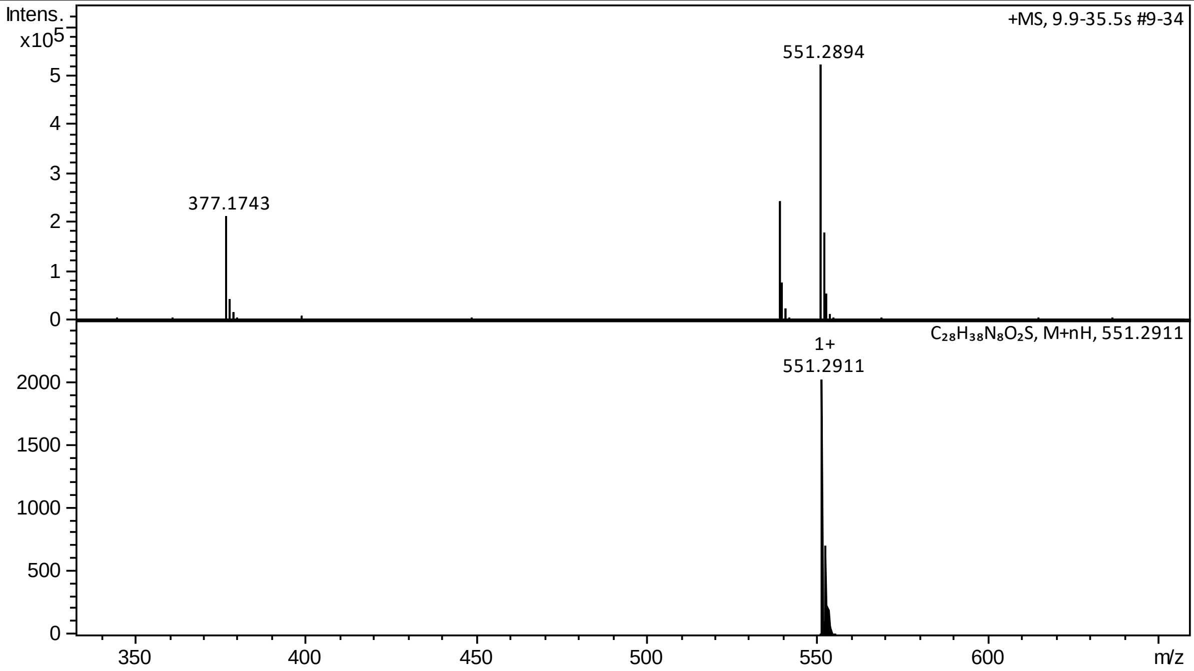

Intens.
x10^5
+MS, 9.9-35.5s #9-34
551.2894
377.1743
C28H38N8O2S, M+nH, 551.2911
1+
551.2911
m/z

4a

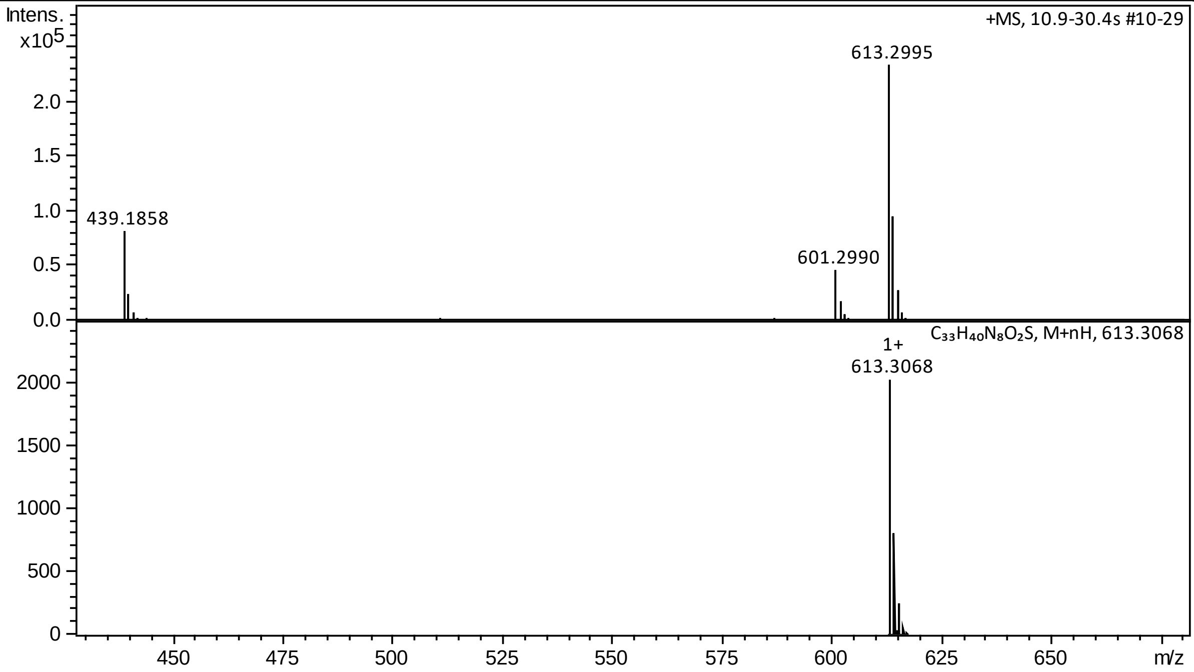

Intens.
x10^5
+MS, 10.9-30.4s #10-29
613.2995
439.1858
601.2990
C33H40N8O2S, M+nH, 613.3068
1+
613.3068
m/z

4b

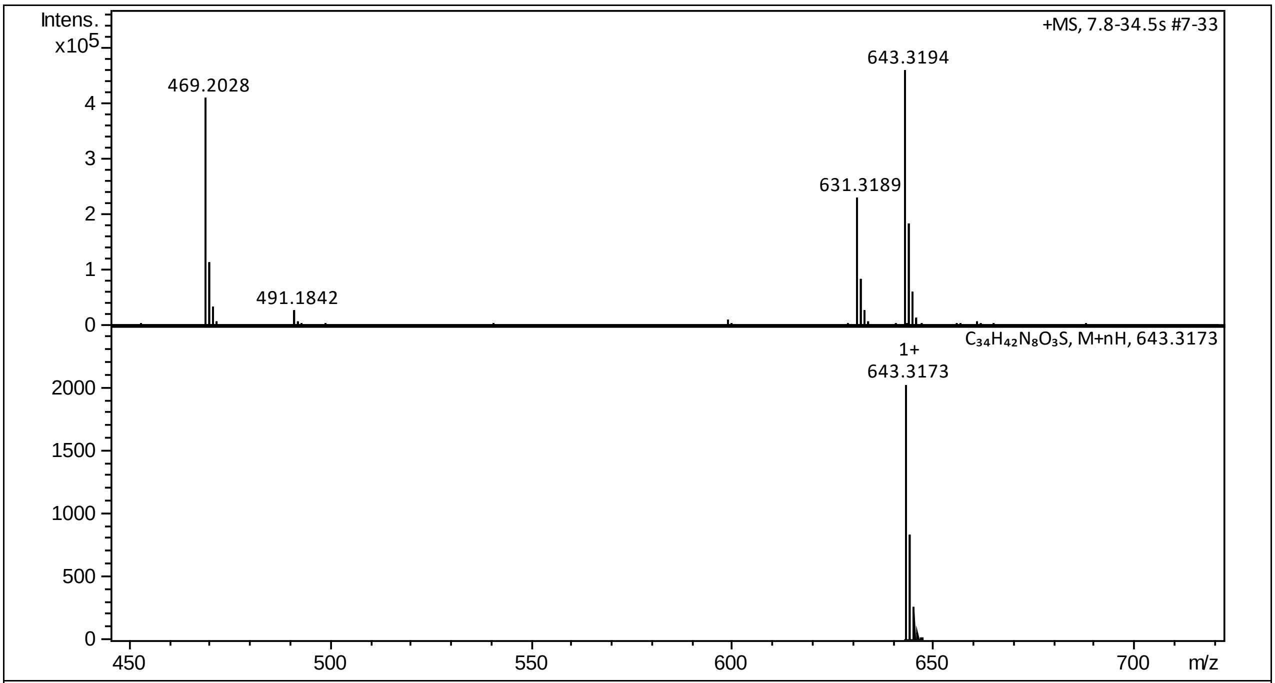
Intens.
x10^5
+MS, 7.8-34.5s #7-33
469.2028
491.1842
631.3189
643.3194
C₃₄H₄₂N₈O₃S, M+nH, 643.3173
1+
643.3173
m/z

4c

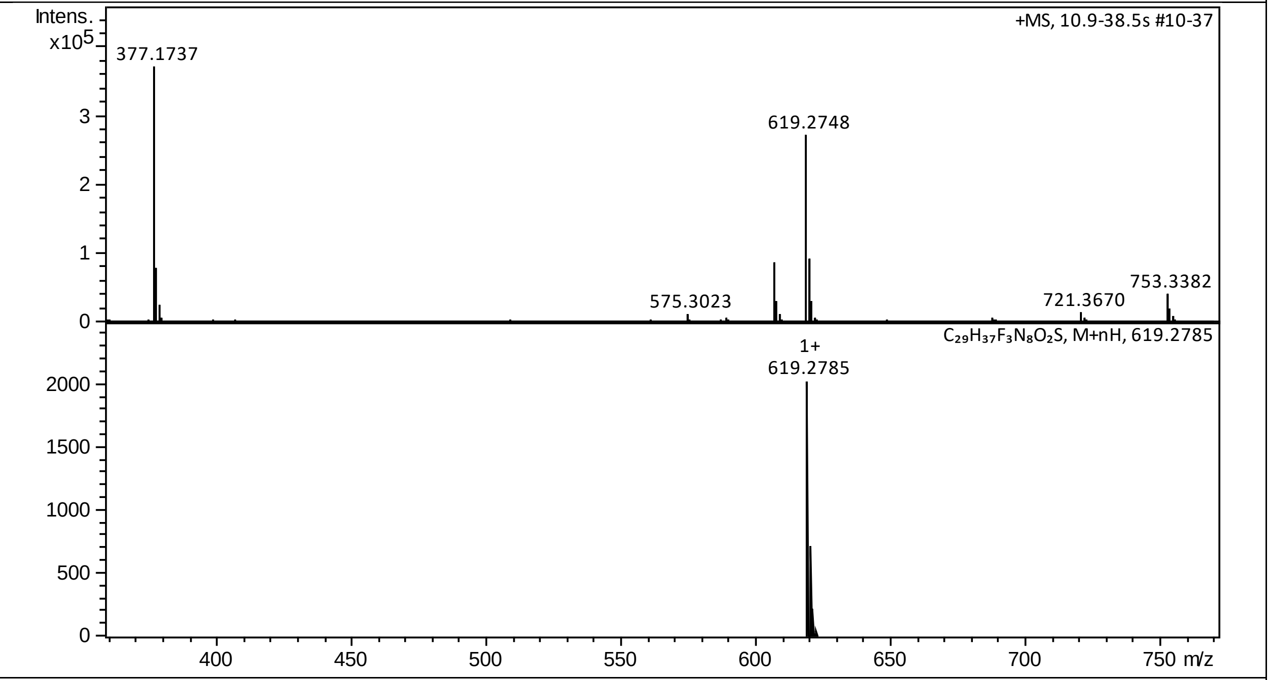
Intens.
x10^5
+MS, 10.9-38.5s #10-37
377.1737
575.3023
619.2748
721.3670
753.3382
C₂₉H₃₇F₃N₈O₂S, M+nH, 619.2785
1+
619.2785
m/z

5a

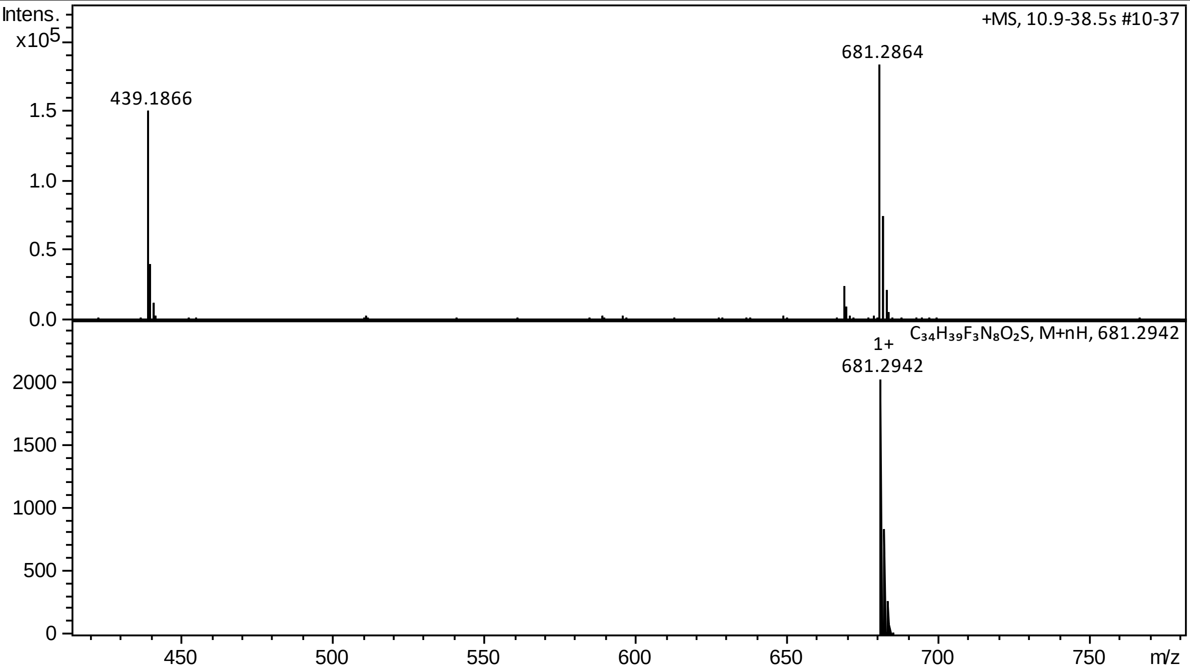

Intens.
x10^5
+MS, 10.9-38.5s #10-37
681.2864
439.1866
C34H39F3N8O2S, M+nH, 681.2942
1+
681.2942
m/z

5b

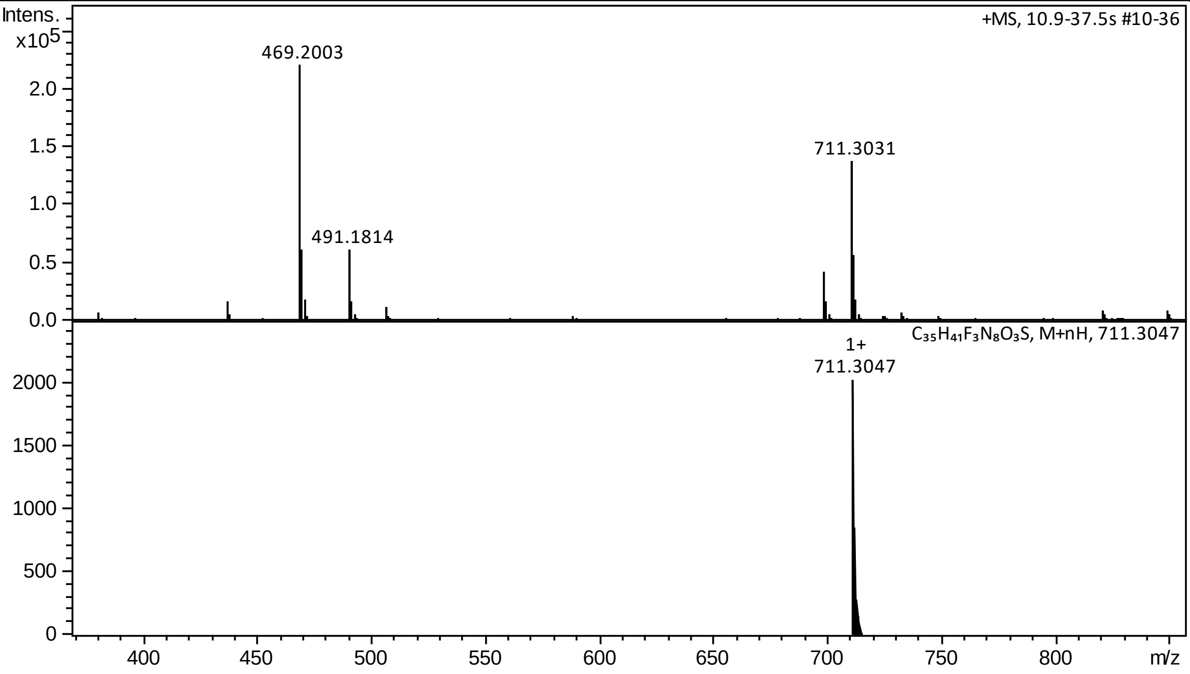

Intens.
x10^5
+MS, 10.9-37.5s #10-36
469.2003
711.3031
491.1814
C35H41F3N8O3S, M+nH, 711.3047
1+
711.3047
m/z

5c

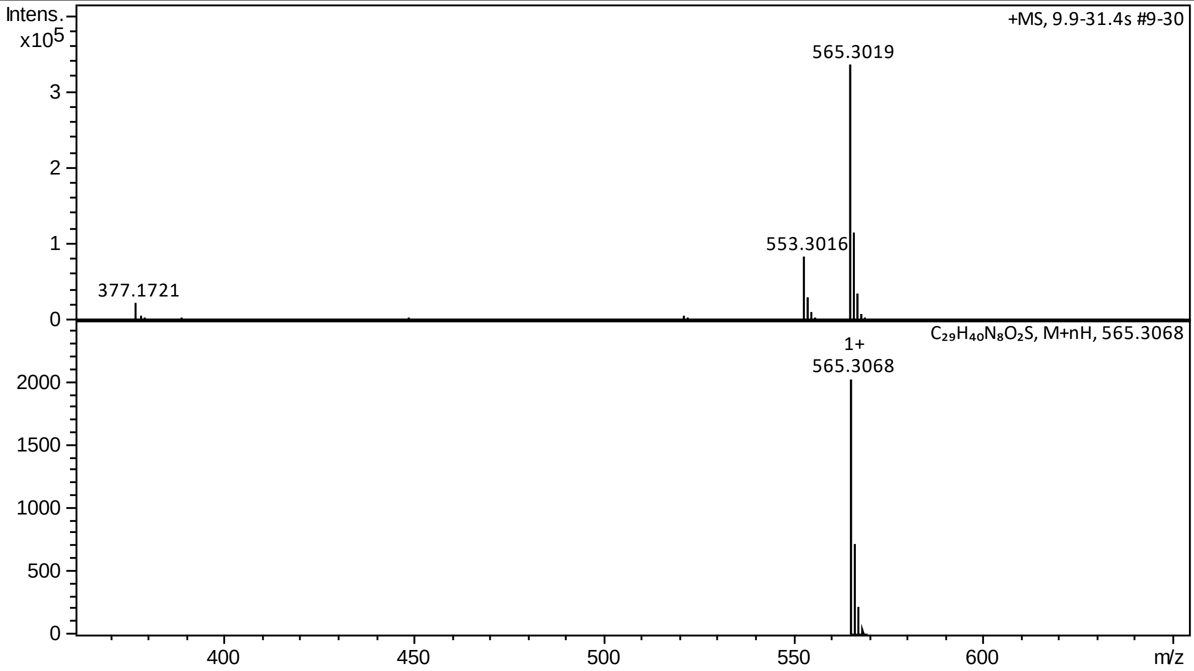
Intens.
x10^5
+MS, 9.9-31.4s #9-30
565.3019
553.3016
377.1721
C₂₉H₄₀N₈O₂S, M+nH, 565.3068
1+
565.3068
m/z

6a

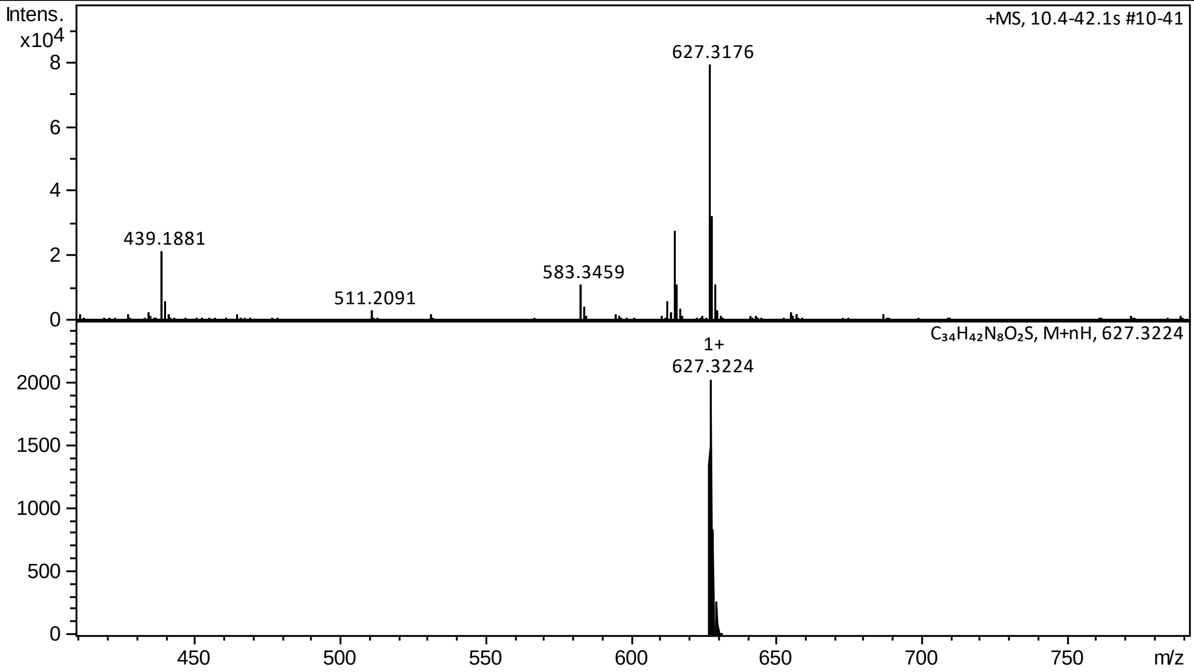
Intens.
x10^4
+MS, 10.4-42.1s #10-41
627.3176
439.1881
583.3459
511.2091
C₃₄H₄₂N₈O₂S, M+nH, 627.3224
1+
627.3224
m/z

6b

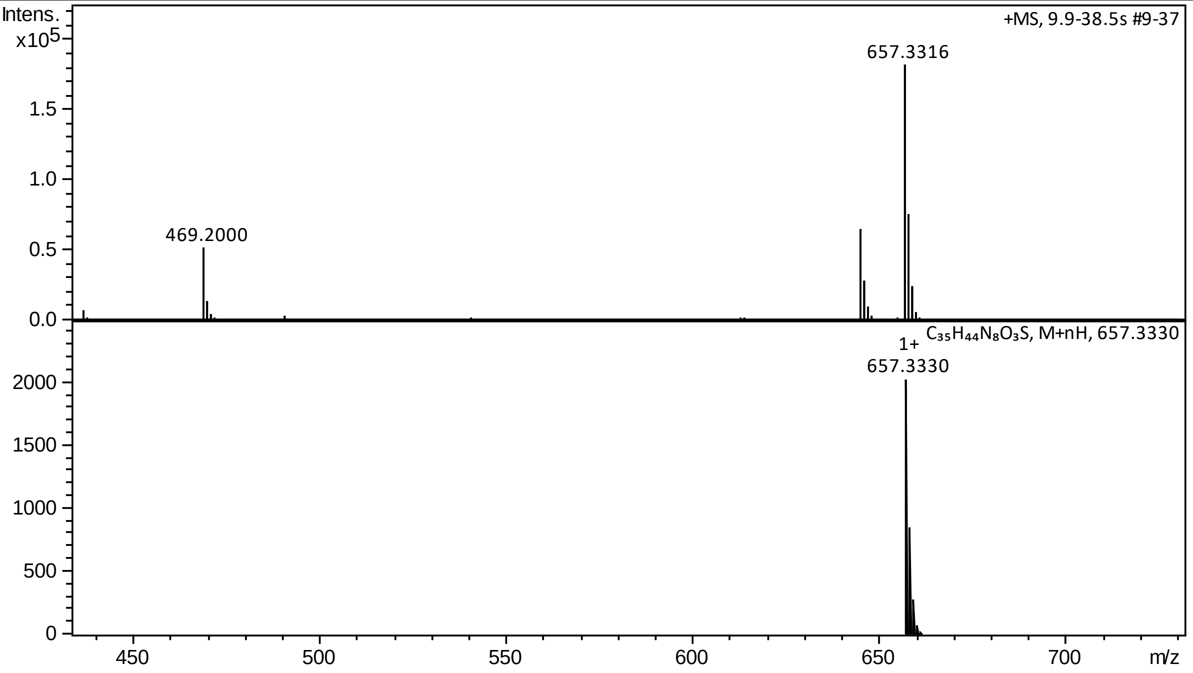
Intens.
x10^5
+MS, 9.9-38.5s #9-37
657.3316
469.2000
C₃₅H₄₄N₈O₃S, M+nH, 657.3330
1+
657.3330
m/z

6c

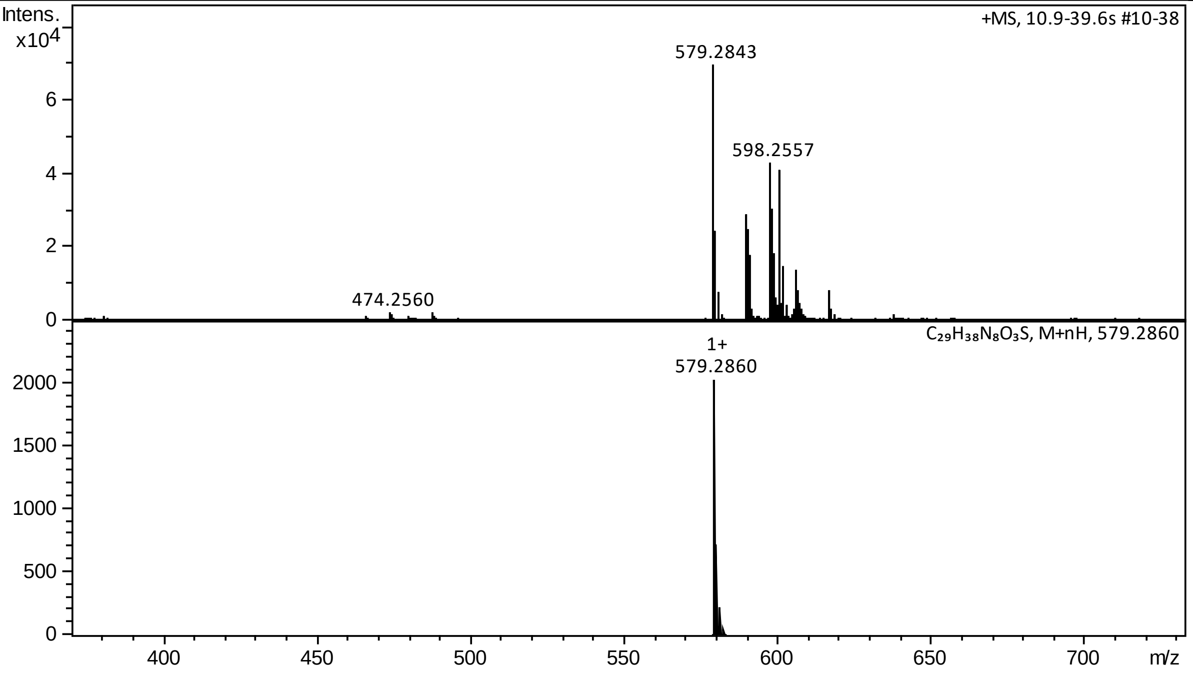
Intens.
x10^4
+MS, 10.9-39.6s #10-38
579.2843
598.2557
474.2560
C₂₉H₃₈N₈O₃S, M+nH, 579.2860
1+
579.2860
m/z

7a

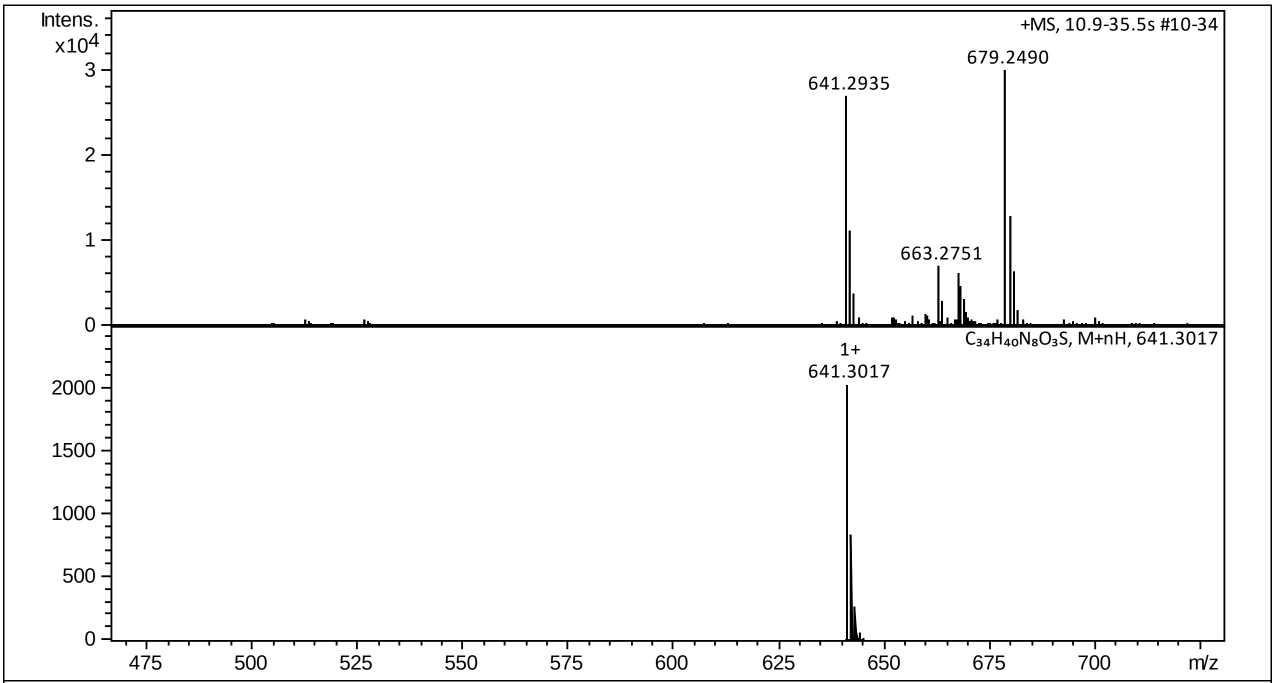

Intens.
x10^4
+MS, 10.9-35.5s #10-34
679.2490
641.2935
663.2751
C₃₄H₄₀N₈O₃S, M+nH, 641.3017
1+
641.3017
m/z

7b

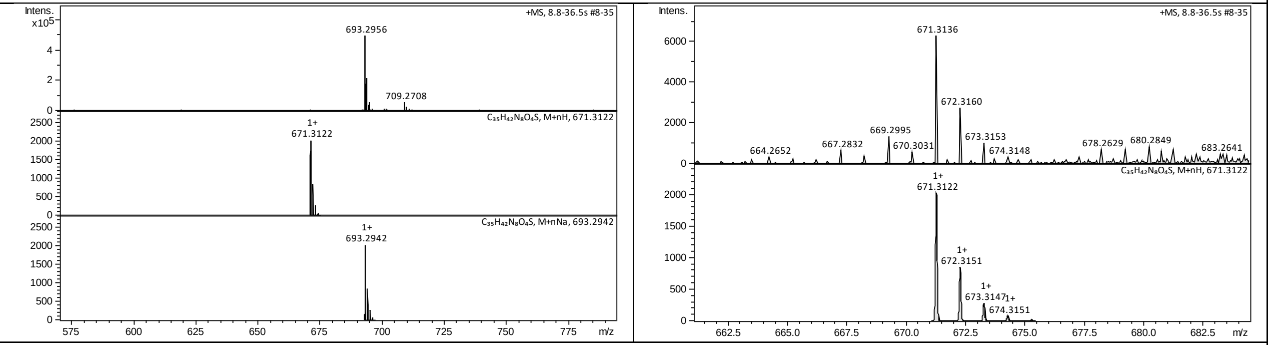

Intens.
+MS, 8.8-36.5s #8-35
693.2956
709.2708
C₃₅H₄₂N₈O₄S, M+nH, 671.3122
1+
671.3122
C₃₅H₄₂N₈O₄S, M+nNa, 693.2942
1+
693.2942
671.3136
672.3160
669.2995
673.3153
667.2832
670.3031
664.2652
674.3148
678.2629
680.2849
683.2641
1+
672.3151
673.3147
674.3151
m/z

7c

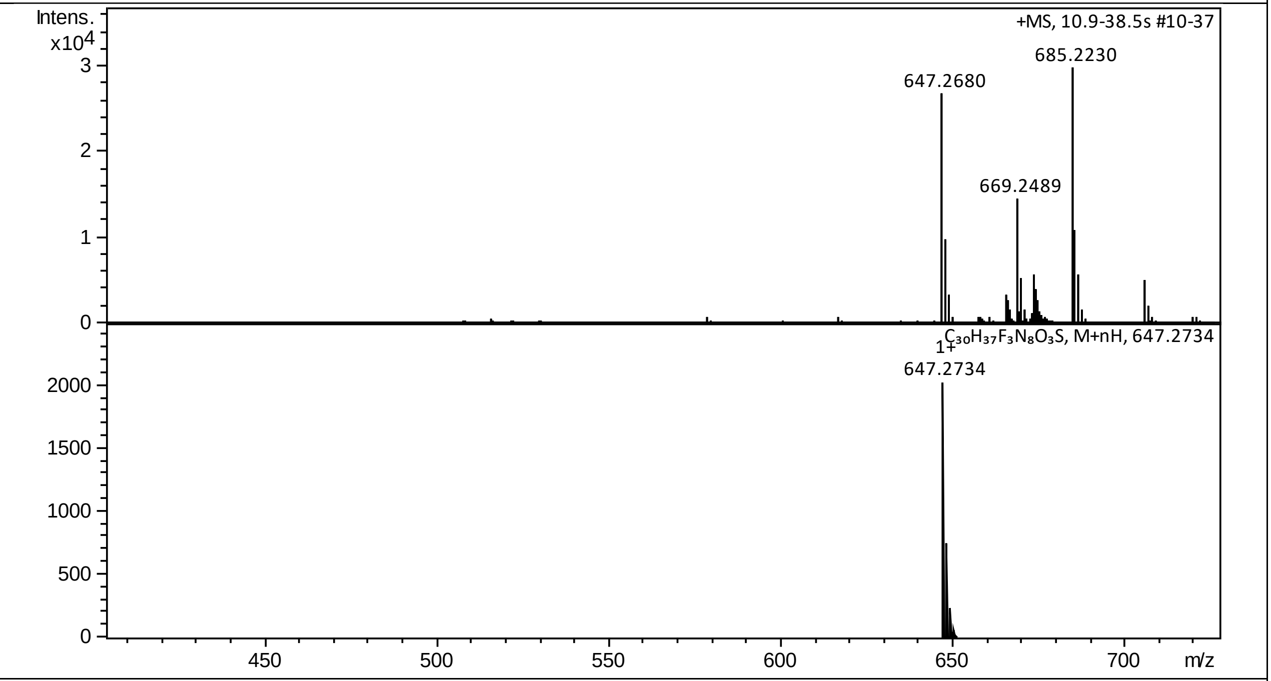

Intens.
x10^4
+MS, 10.9-38.5s #10-37
685.2230
647.2680
669.2489
C₃₀H₃₇F₃N₈O₃S, M+nH, 647.2734
1+
647.2734
m/z

8a

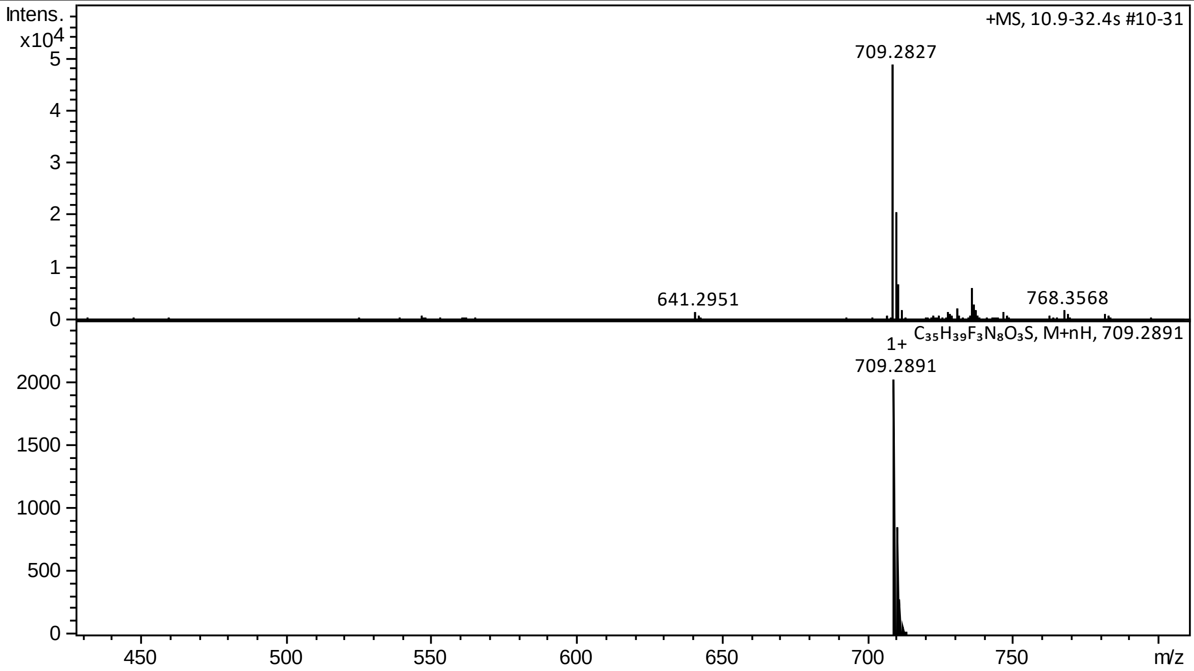
Intens.
x10^4
+MS, 10.9-32.4s #10-31
709.2827
641.2951
768.3568
C₃₅H₃₉F₃N₈O₃S, M+nH, 709.2891
1+
709.2891
m/z

8b

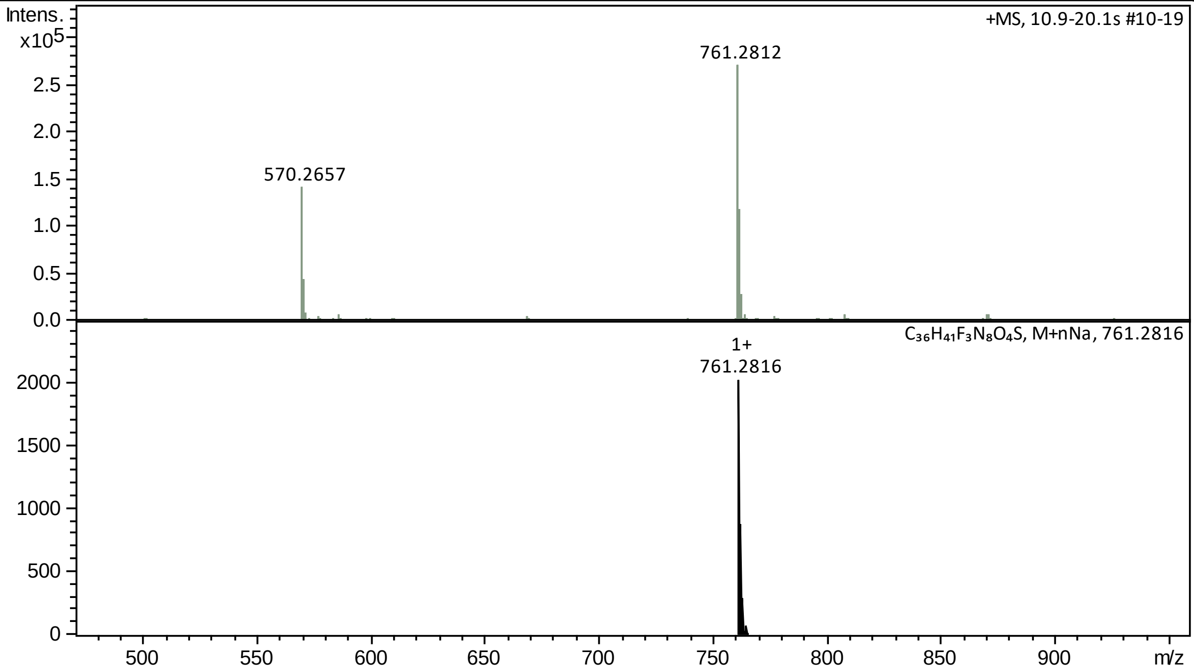
Intens.
x10^5
+MS, 10.9-20.1s #10-19
761.2812
570.2657
C₃₆H₄₁F₃N₈O₄S, M+nNa, 761.2816
1+
761.2816
m/z

8c

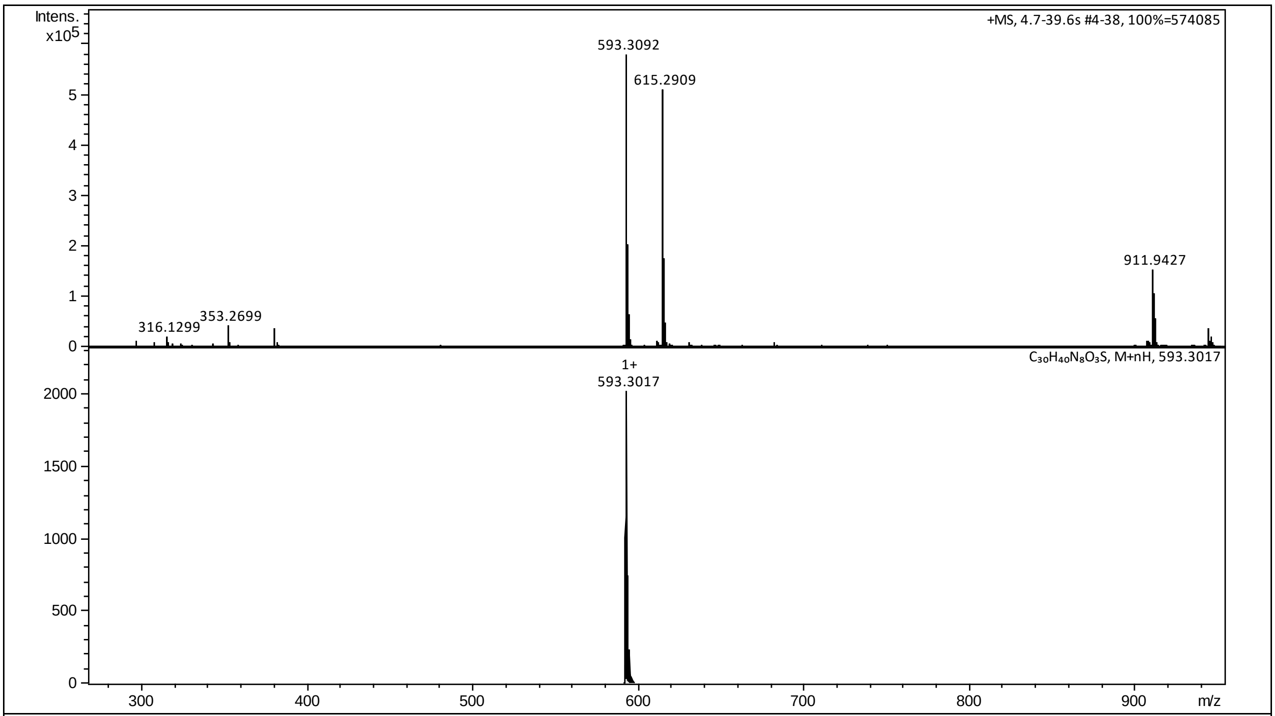
Intens.
x10^5
+MS, 4.7-39.6s #4-38, 100%=574085
593.3092
615.2909
911.9427
316.1299
353.2699
$C_{30}H_{40}N_8O_3S$, M+nH, 593.3017
1+
593.3017
m/z

9a

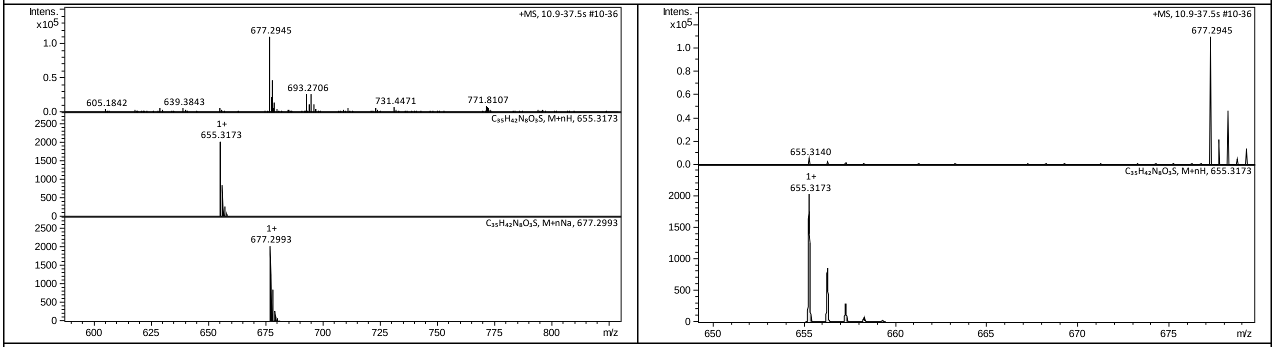
Intens.
x10^5
+MS, 10.9-37.5s #10-36
677.2945
605.1842
639.3843
693.2706
731.4471
771.8107
$C_{35}H_{42}N_8O_3S$, M+nH, 655.3173
1+
655.3173
$C_{35}H_{42}N_8O_3S$, M+nNa, 677.2993
1+
677.2993
m/z
Intens.
x10^5
+MS, 10.9-37.5s #10-36
677.2945
655.3140
$C_{35}H_{42}N_8O_3S$, M+nH, 655.3173
1+
655.3173
m/z

9b

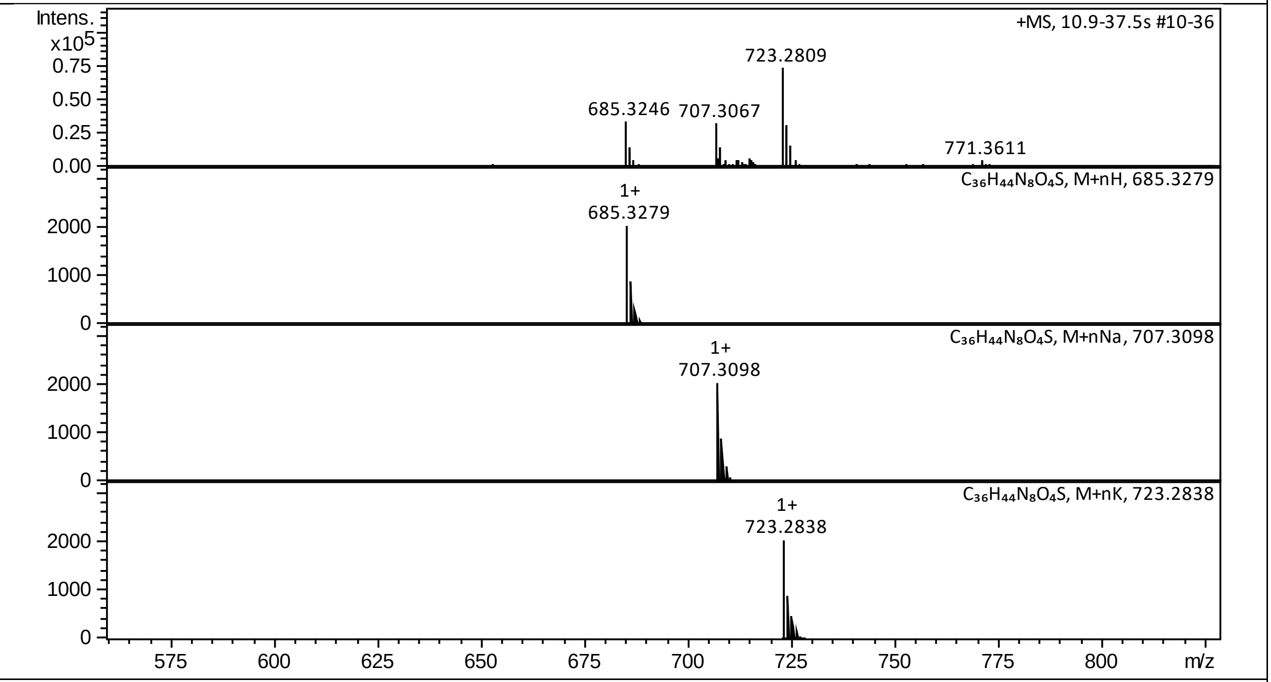
Intens.
x10^5
+MS, 10.9-37.5s #10-36
723.2809
685.3246
707.3067
771.3611
$C_{36}H_{44}N_8O_4S$, M+nH, 685.3279
1+
685.3279
$C_{36}H_{44}N_8O_4S$, M+nNa, 707.3098
1+
707.3098
$C_{36}H_{44}N_8O_4S$, M+nK, 723.2838
1+
723.2838
m/z

9c

Table S4 – FT-IR spectra of new compounds

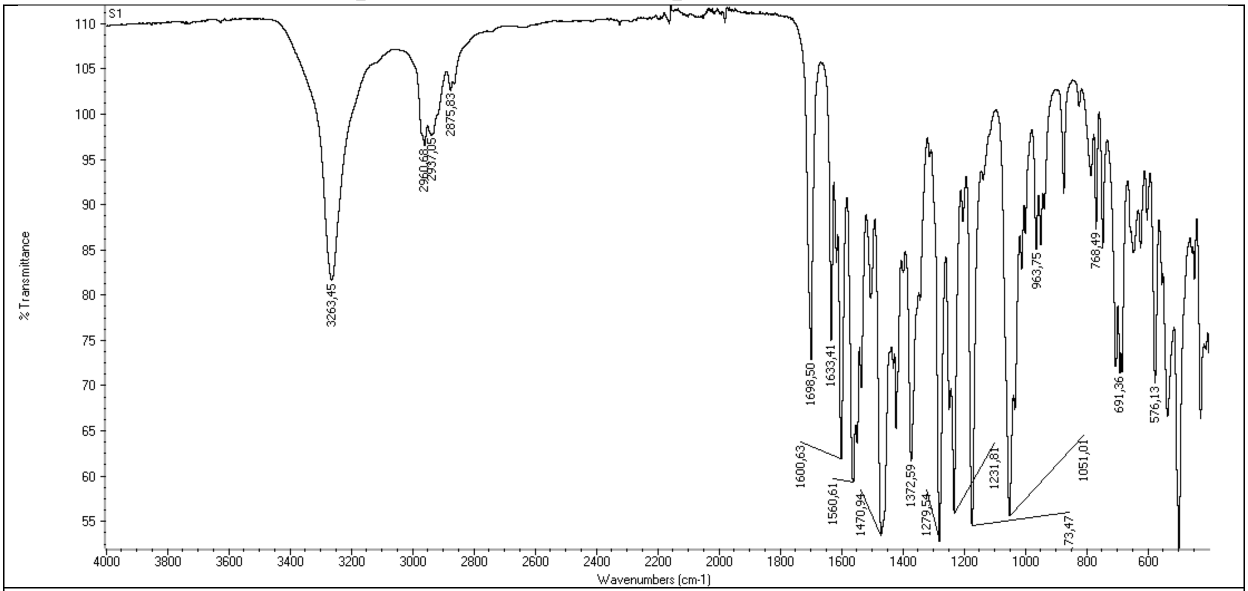

2a

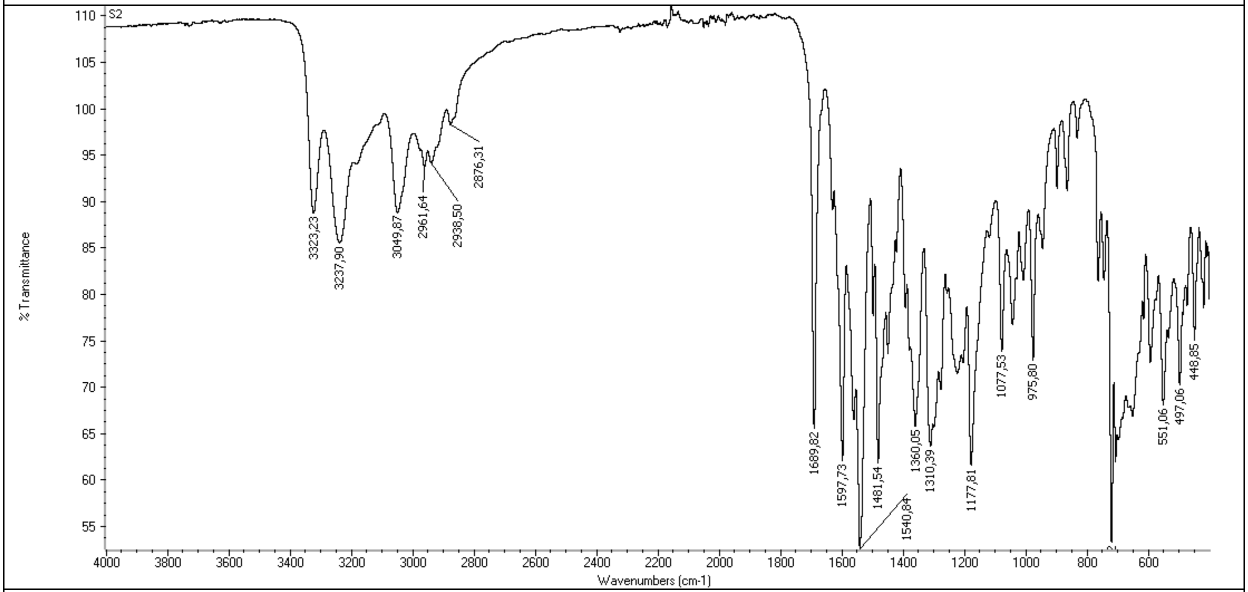

2b

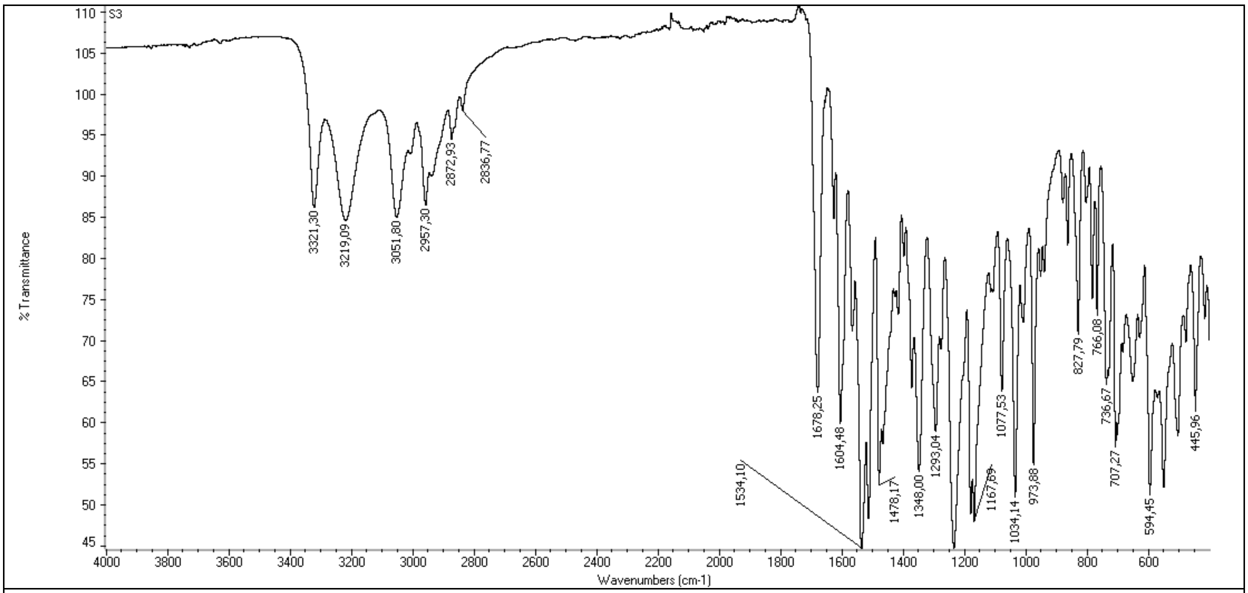
S3
% Transmittance
Wavenumbers (cm-1)
3321,30
3219,09
3051,80
2957,30
2872,93
2836,77
1678,25
1604,48
1534,10
1478,17
1348,00
1293,04
1167,69
1077,53
1034,14
973,88
827,79
766,08
736,67
707,27
594,45
445,96

2c

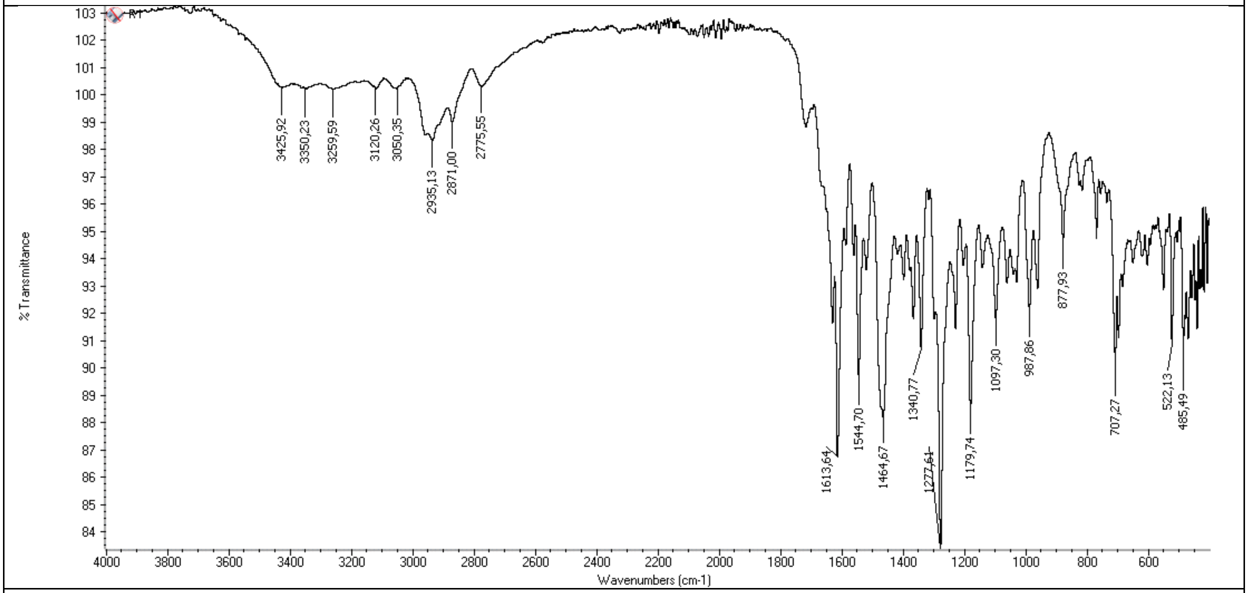
R1
% Transmittance
Wavenumbers (cm-1)
3425,92
3350,23
3259,59
3120,26
3050,35
2935,13
2871,00
2775,55
1613,64
1544,70
1464,67
1340,77
1277,61
1179,74
1097,30
987,86
877,93
707,27
522,13
485,49

3a

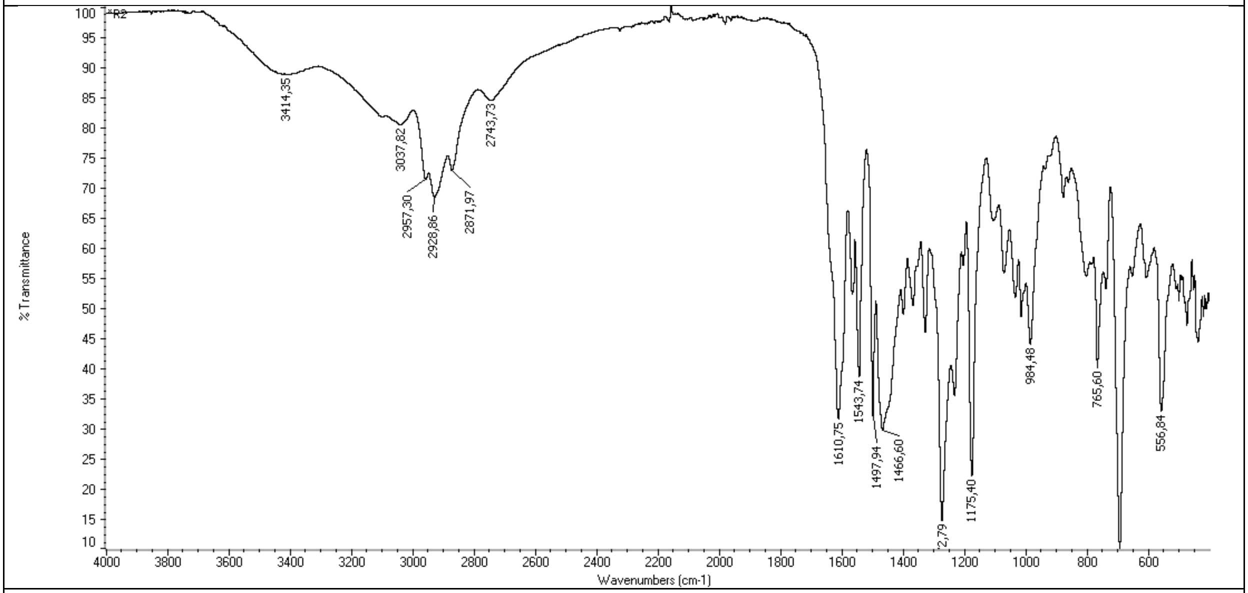
R2
% Transmittance
Wavenumbers (cm-1)
3414,35
3037,82
2957,30
2928,86
2871,97
2743,73
1610,75
1543,74
1497,94
1466,60
2,79
1175,40
984,48
765,60
556,84

3b

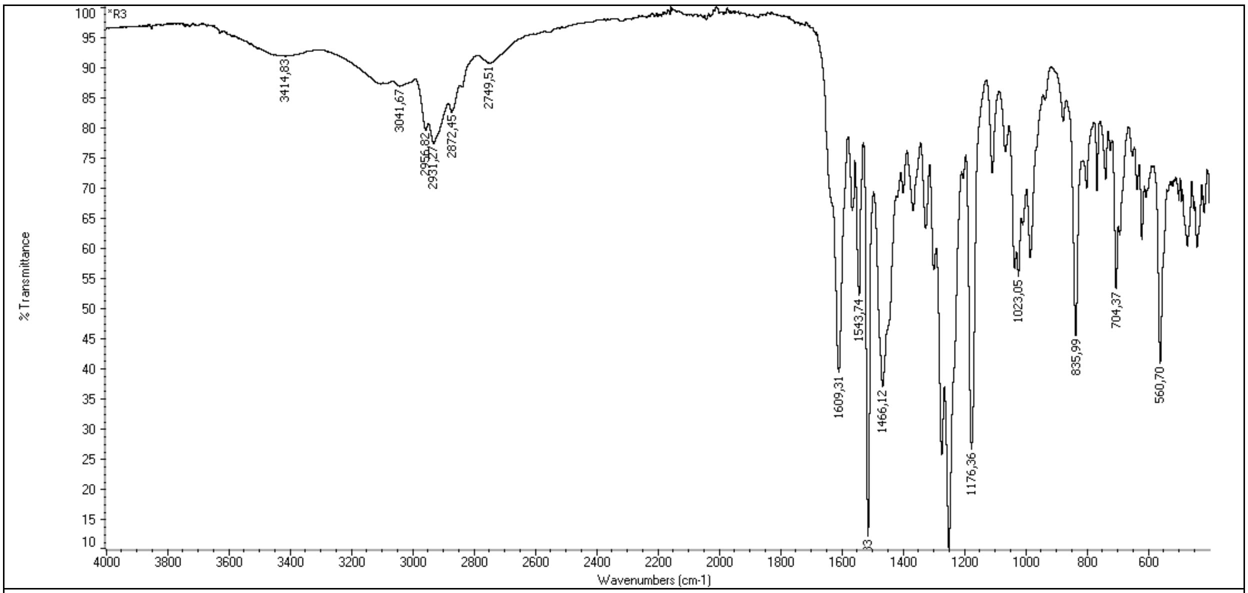
*R3
% Transmittance
Wavenumbers (cm-1)
3414,83
3041,67
2956,82
2931,27
2872,45
2749,51
1609,31
1543,74
33
1466,12
1176,36
1023,05
835,99
704,37
560,70

3c

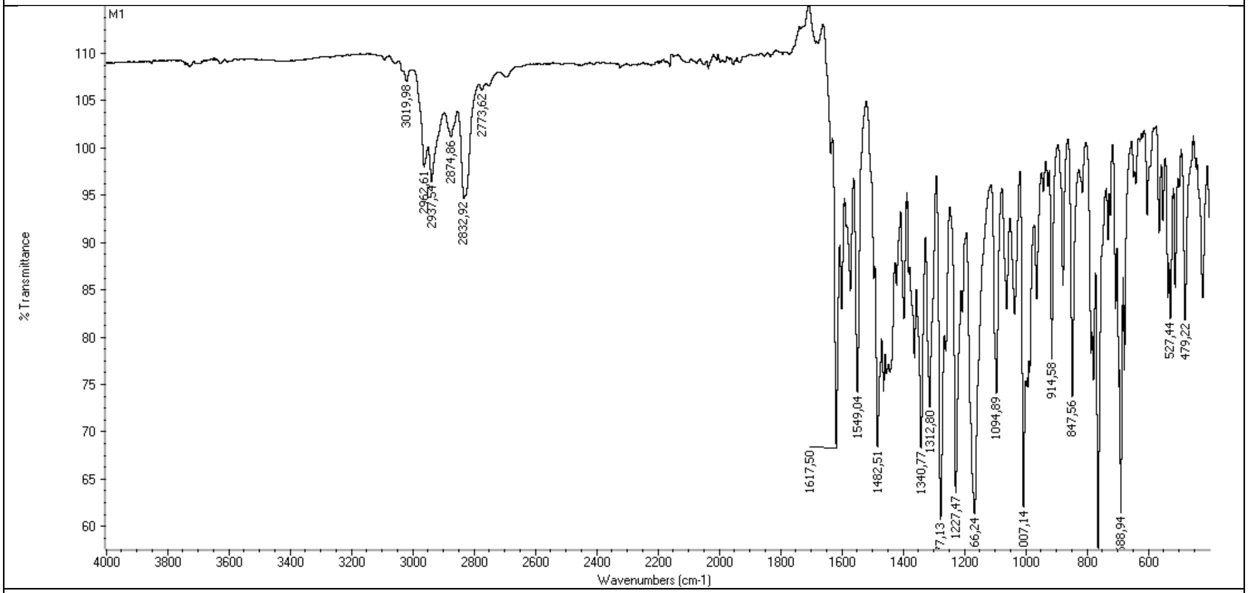
M1
% Transmittance
Wavenumbers (cm-1)
3019,98
2962,61
2937,34
2874,86
2832,92
2773,62
1617,50
1549,04
1482,51
1340,77
1312,80
77,13
1227,47
66,24
1094,89
007,14
914,58
847,56
688,94
527,44
479,22

4a

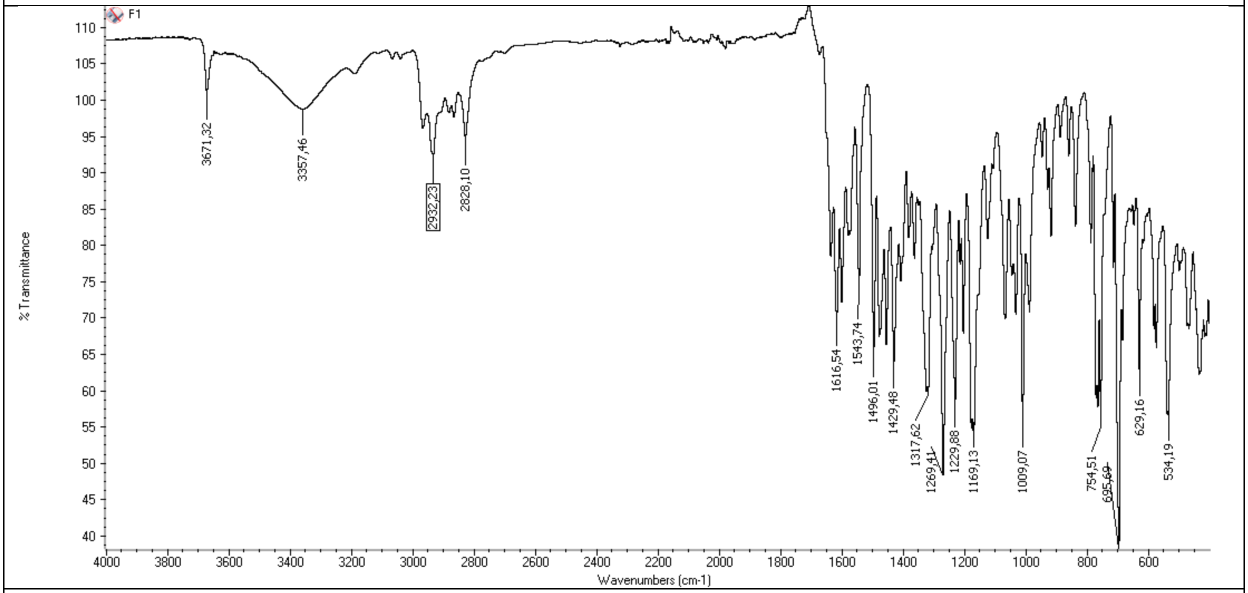
F1
% Transmittance
Wavenumbers (cm-1)
3671,32
3357,46
2932,23
2828,10
1616,54
1543,74
1496,01
1429,48
1317,62
1269,41
1229,88
1169,13
1009,07
754,51
695,69
629,16
534,19

4b

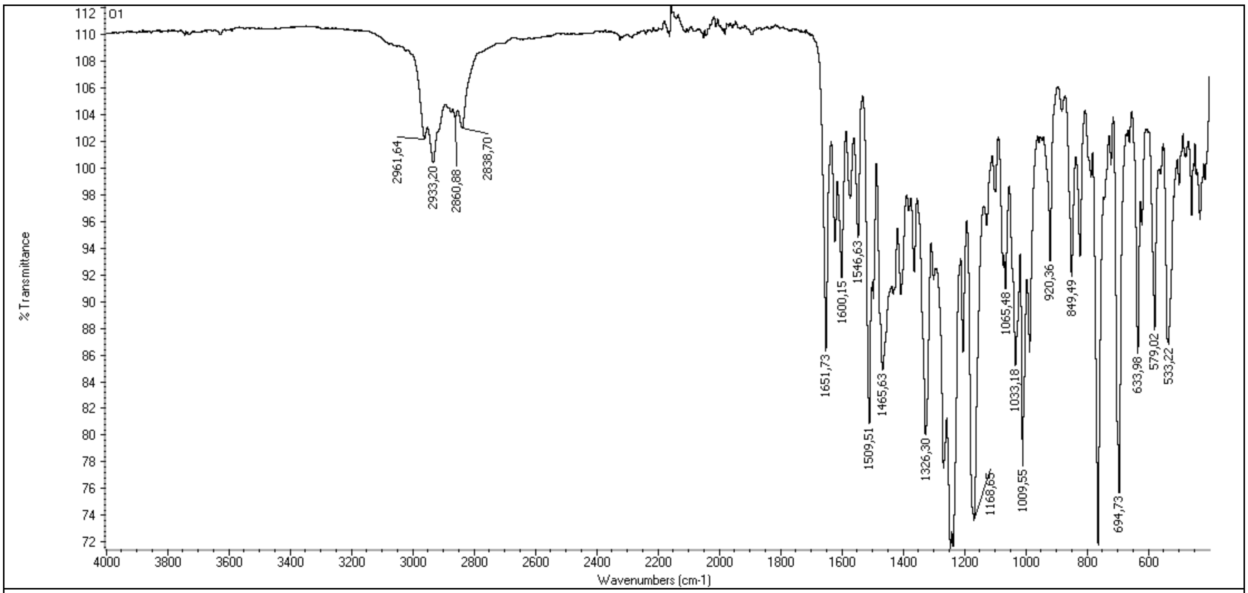
O1
% Transmittance
Wavenumbers (cm-1)
2961,64
2933,20
2860,88
2838,70
1651,73
1600,15
1546,63
1509,51
1465,63
1326,30
1168,65
1065,48
1033,18
1009,55
920,36
849,49
694,73
633,98
579,02
533,22

4c

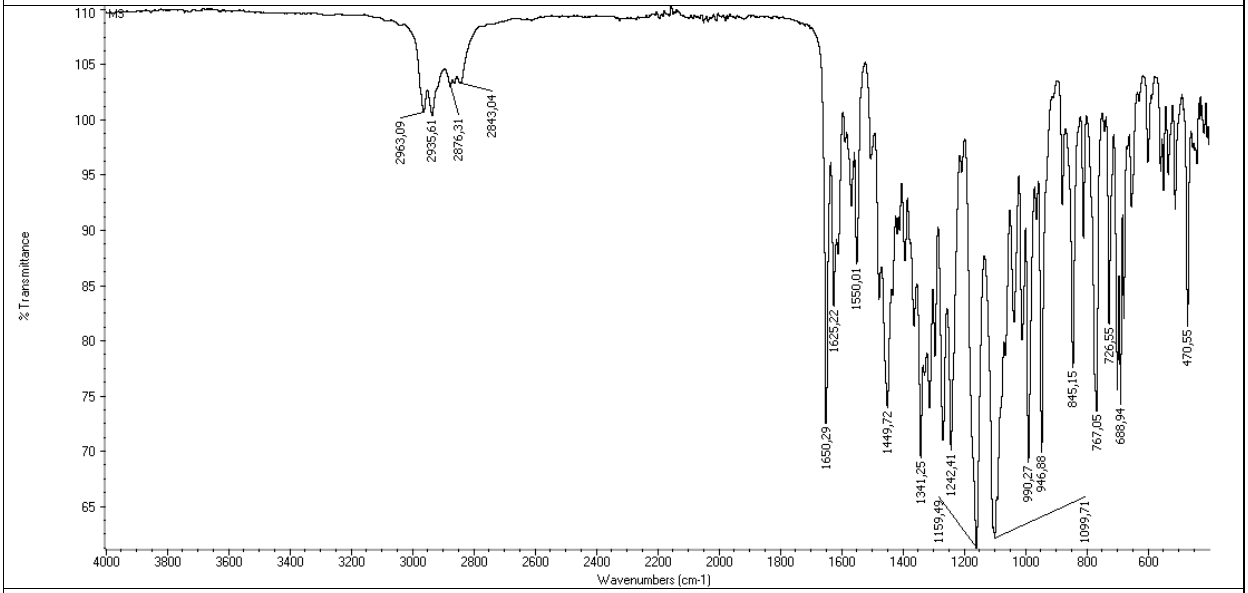
MS
% Transmittance
Wavenumbers (cm-1)
2963,09
2935,61
2876,31
2843,04
1650,29
1625,22
1550,01
1449,72
1341,25
1242,41
1159,49
1099,71
990,27
946,88
845,15
767,05
726,55
688,94
470,55

5a

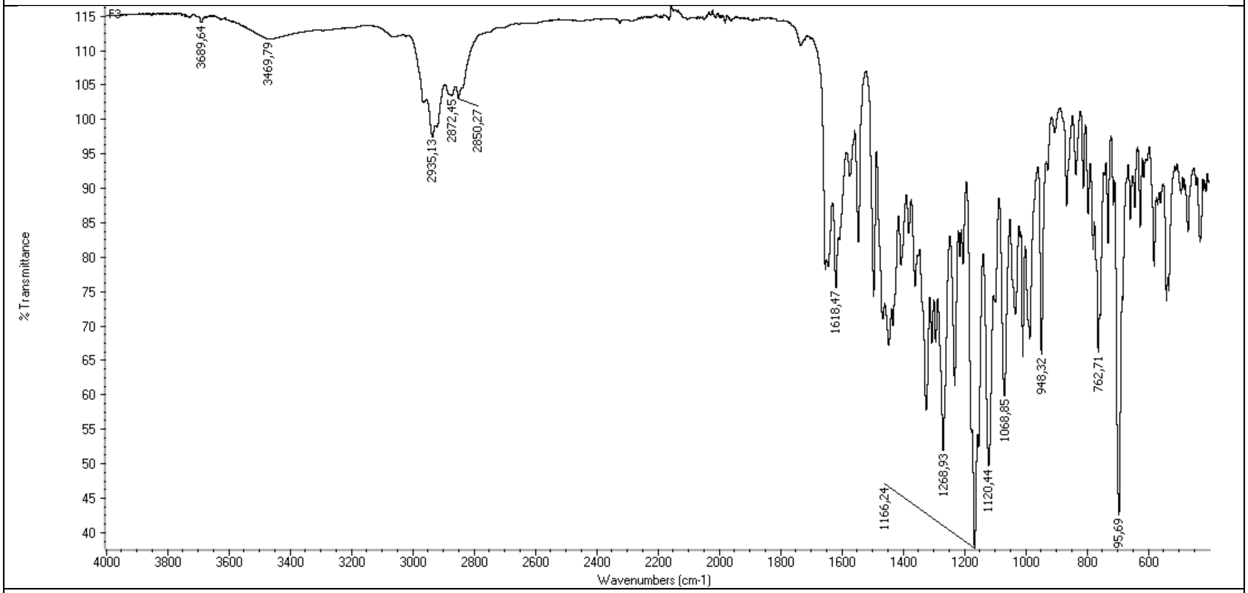
F3
% Transmittance
Wavenumbers (cm-1)
3689,64
3469,79
2935,13
2872,45
2850,27
1618,47
1268,93
1166,24
1120,44
1068,85
948,32
762,71
95,69

5b

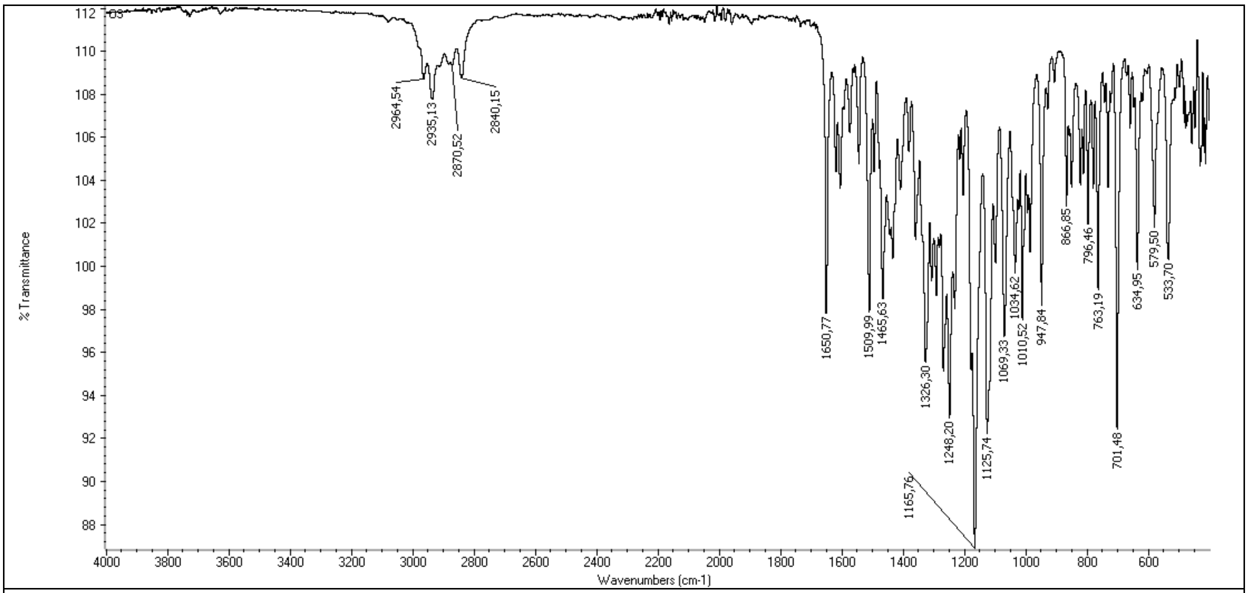
% Transmittance
Wavenumbers (cm-1)
2964,54
2935,13
2870,52
2840,15
1650,77
1509,99
1465,63
1326,30
1248,20
1165,76
1125,74
1069,33
1034,62
1010,52
947,84
866,85
796,46
763,19
701,48
634,95
579,50
533,70

5c

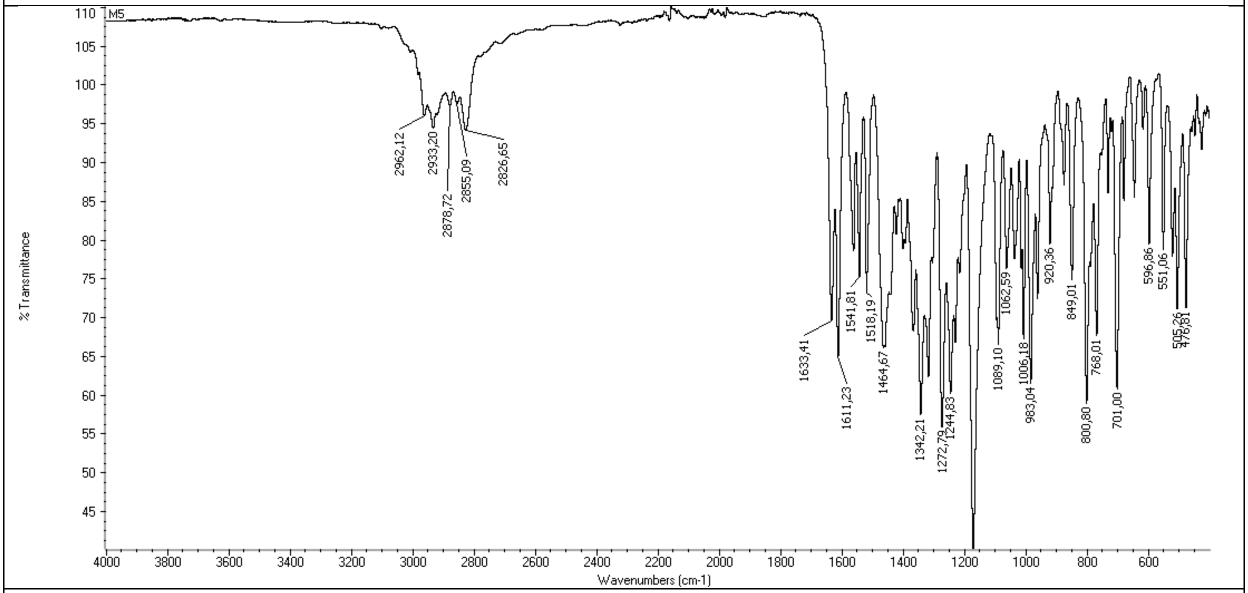
M5
% Transmittance
Wavenumbers (cm-1)
2962,12
2933,20
2878,72
2855,09
2826,65
1633,41
1611,23
1541,81
1518,19
1464,67
1342,21
1272,79
1244,83
1089,10
1062,59
1006,18
983,04
920,36
849,01
800,80
768,01
701,00
596,86
551,06
505,26
476,61

6a

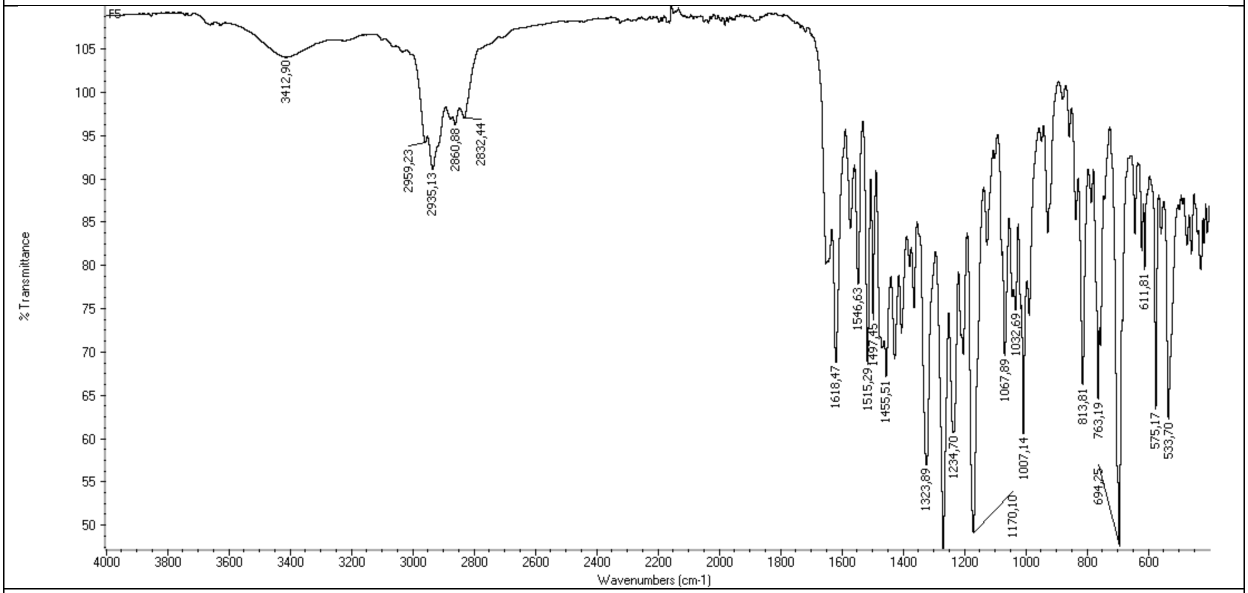
F5
% Transmittance
Wavenumbers (cm-1)
3412,90
2959,23
2935,13
2860,88
2832,44
1618,47
1546,63
1515,29
1497,45
1455,51
1323,89
1234,70
1170,10
1067,89
1032,69
1007,14
813,81
763,19
694,25
611,81
575,17
533,70

6b

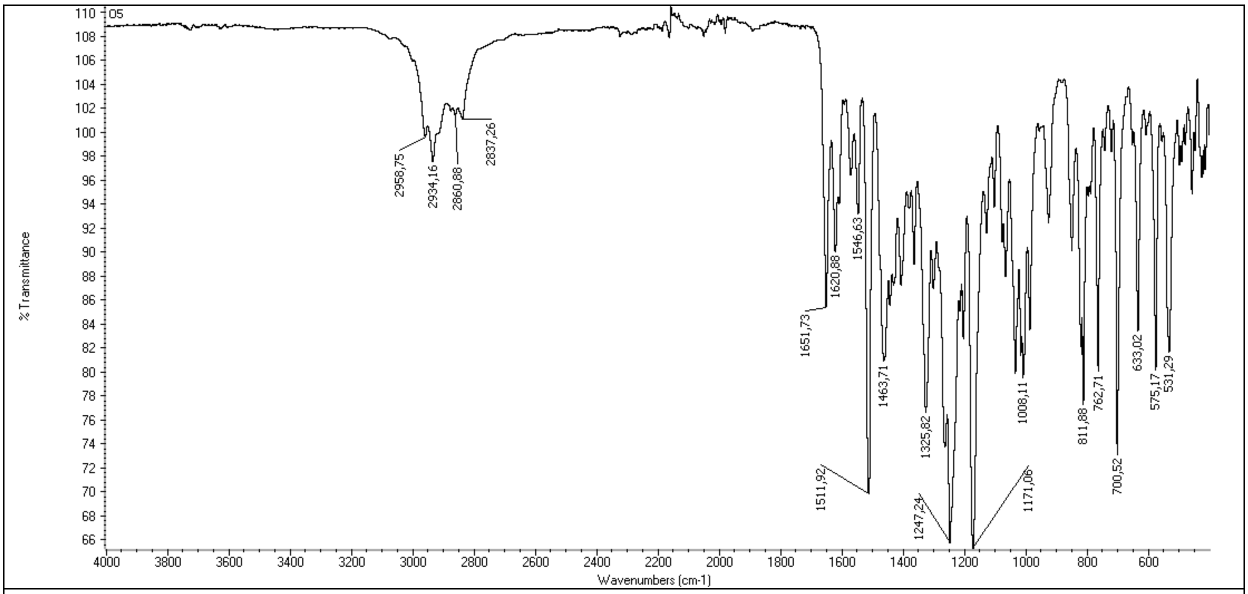
O5
% Transmittance
Wavenumbers (cm-1)
2958,75
2934,16
2860,88
2837,26
1651,73
1620,88
1546,63
1511,92
1463,71
1325,82
1247,24
1171,06
1008,11
811,88
762,71
700,52
633,02
575,17
531,29

6c

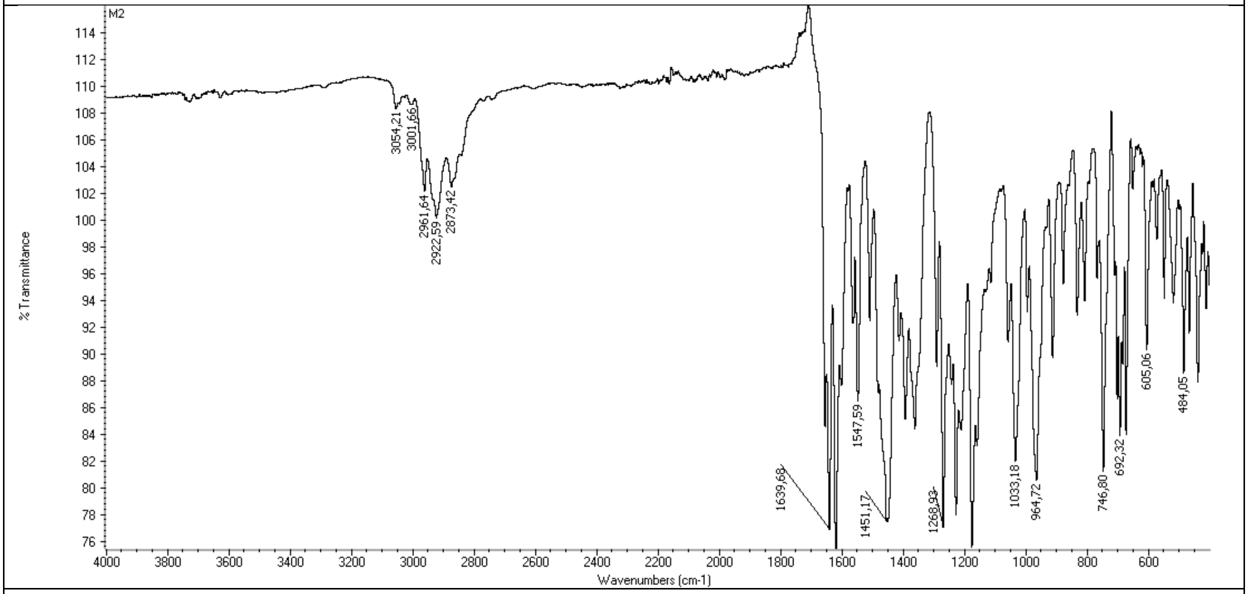
M2
% Transmittance
Wavenumbers (cm-1)
3054,21
3001,66
2961,64
2922,59
2873,42
1639,68
1547,59
1451,17
1268,93
1033,18
964,72
746,80
692,32
605,06
484,05

7a

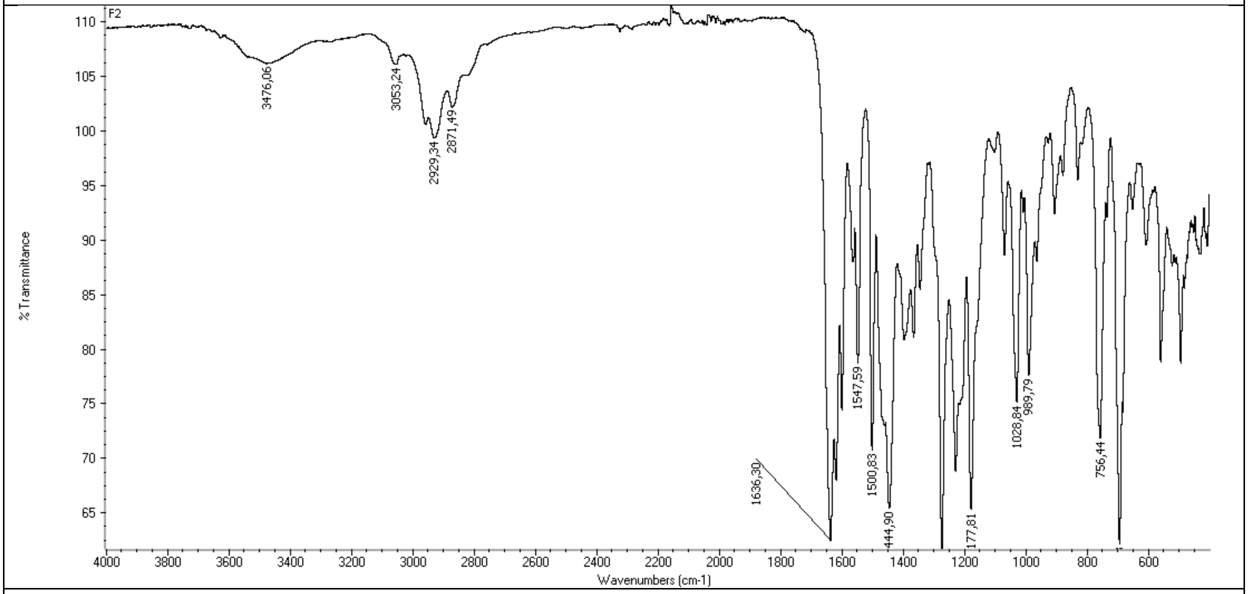
F2
% Transmittance
Wavenumbers (cm-1)
3476,06
3053,24
2929,34
2871,49
1636,30
1547,59
1500,83
444,90
177,81
1028,84
989,79
756,44

7b

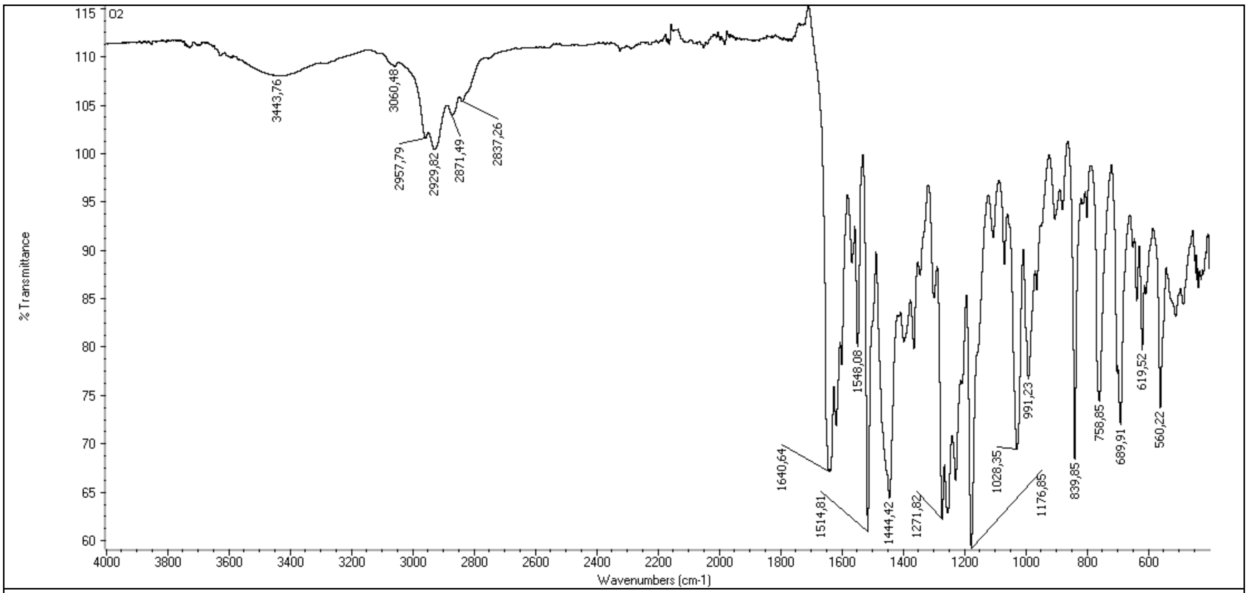
O2
% Transmittance
Wavenumbers (cm-1)
3443,76
3060,48
2957,79
2929,82
2871,49
2837,26
1640,64
1548,08
1514,81
1444,42
1271,82
1176,85
1028,35
991,23
839,85
758,85
689,91
619,52
560,22

7c

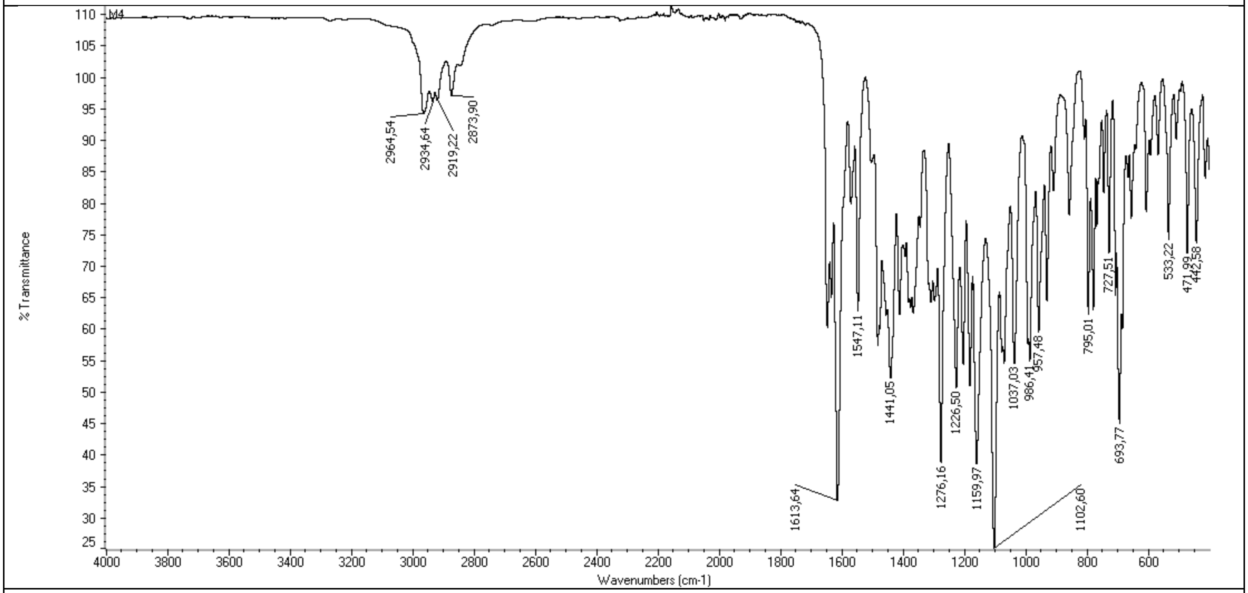
M4
% Transmittance
Wavenumbers (cm-1)
2964,54
2934,64
2919,22
2873,90
1613,64
1547,11
1441,05
1276,16
1226,50
1159,97
1102,60
1037,03
986,41
957,48
795,01
727,51
693,77
533,22
471,99
442,58

8a

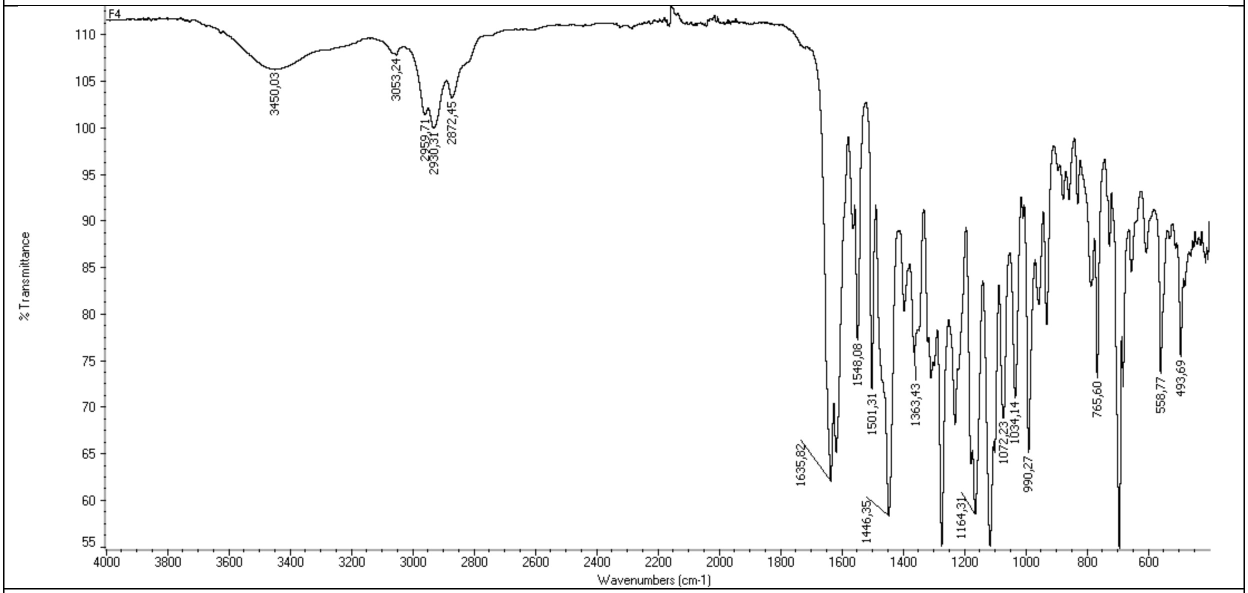
F4
% Transmittance
Wavenumbers (cm-1)
3450,03
3053,24
2959,71
2930,31
2872,45
1635,82
1548,08
1501,31
1446,35
1363,43
1164,31
1072,23
1034,14
990,27
765,60
558,77
493,69

8b

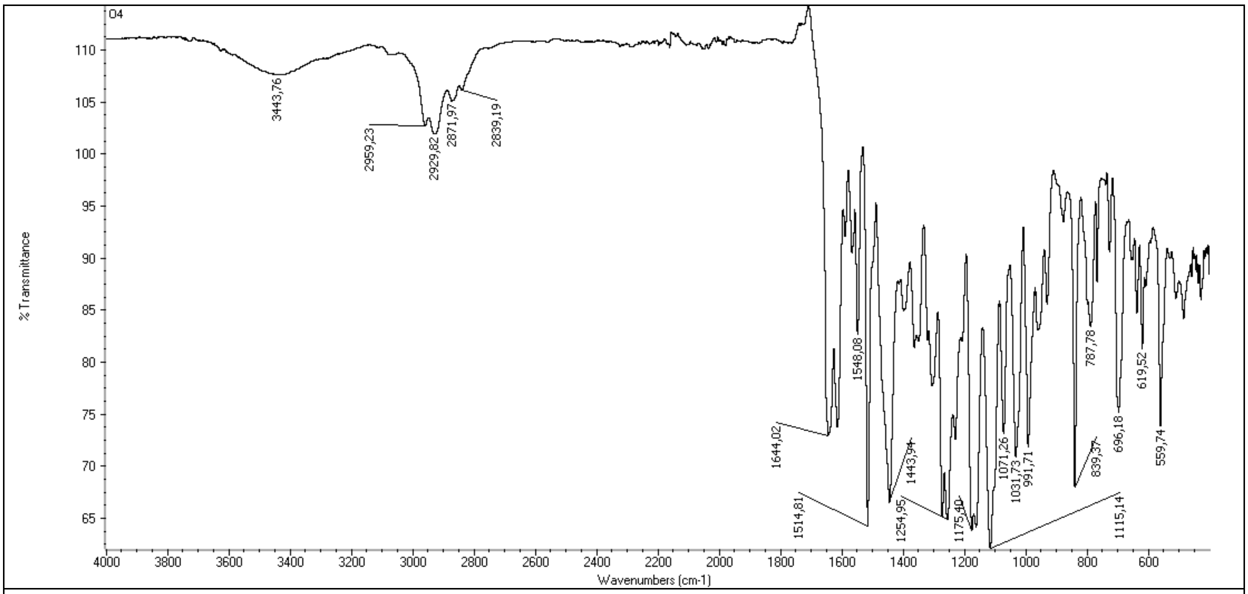
O4
% Transmittance
Wavenumbers (cm-1)
3443,76
2959,23
2929,82
2871,97
2839,19
1644,02
1514,81
1548,08
1443,94
1254,95
1175,40
1115,14
1071,26
1031,73
991,71
839,37
787,78
696,18
619,52
559,74
8c

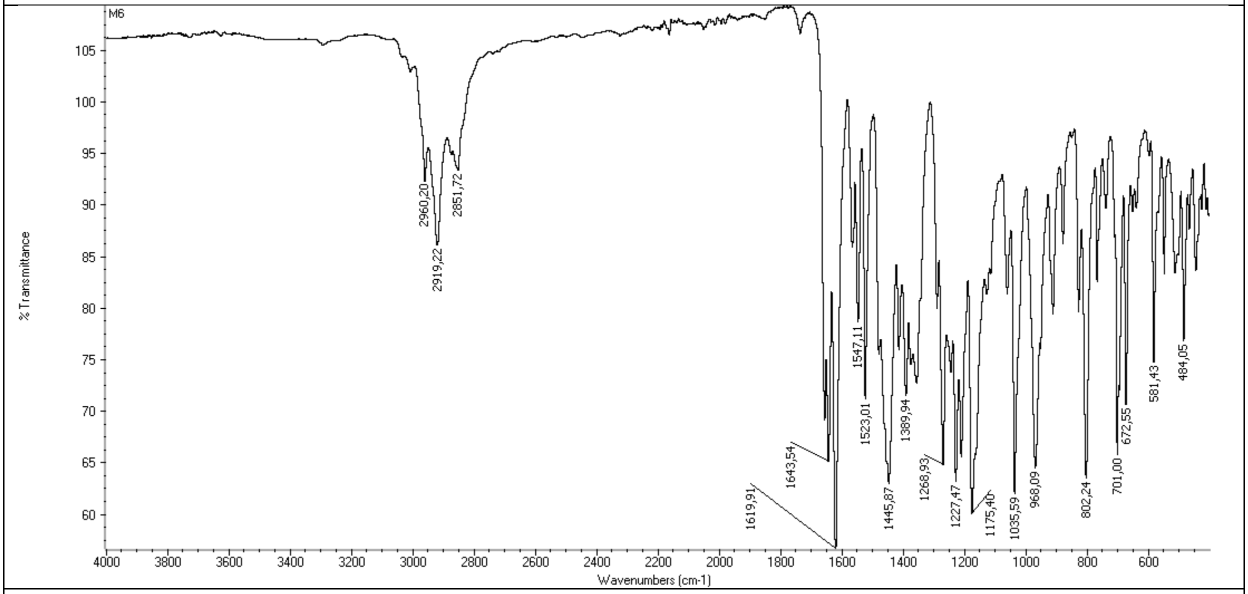
M6
% Transmittance
Wavenumbers (cm-1)
2960,20
2919,22
2851,72
1619,91
1643,54
1547,11
1523,01
1445,87
1389,94
1268,93
1227,47
1175,40
1035,59
968,09
802,24
701,00
672,55
581,43
484,05
9a

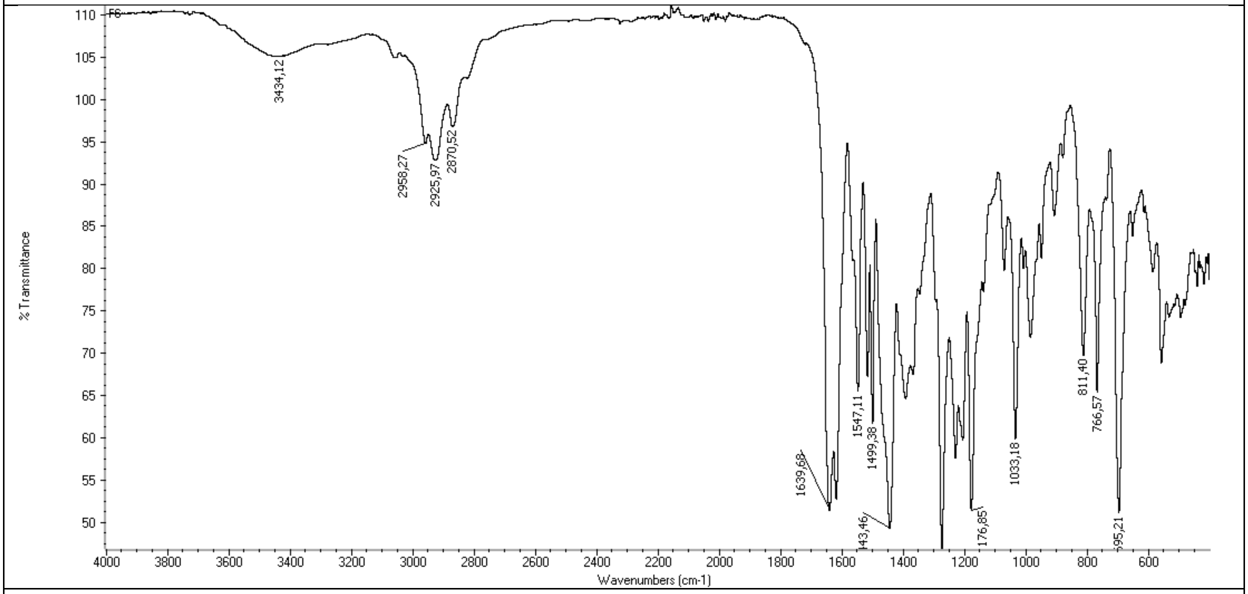
F6
% Transmittance
Wavenumbers (cm-1)
3434,12
2958,27
2925,97
2870,52
1639,68
1547,11
1499,38
143,46
176,85
1033,18
811,40
766,57
695,21
9b

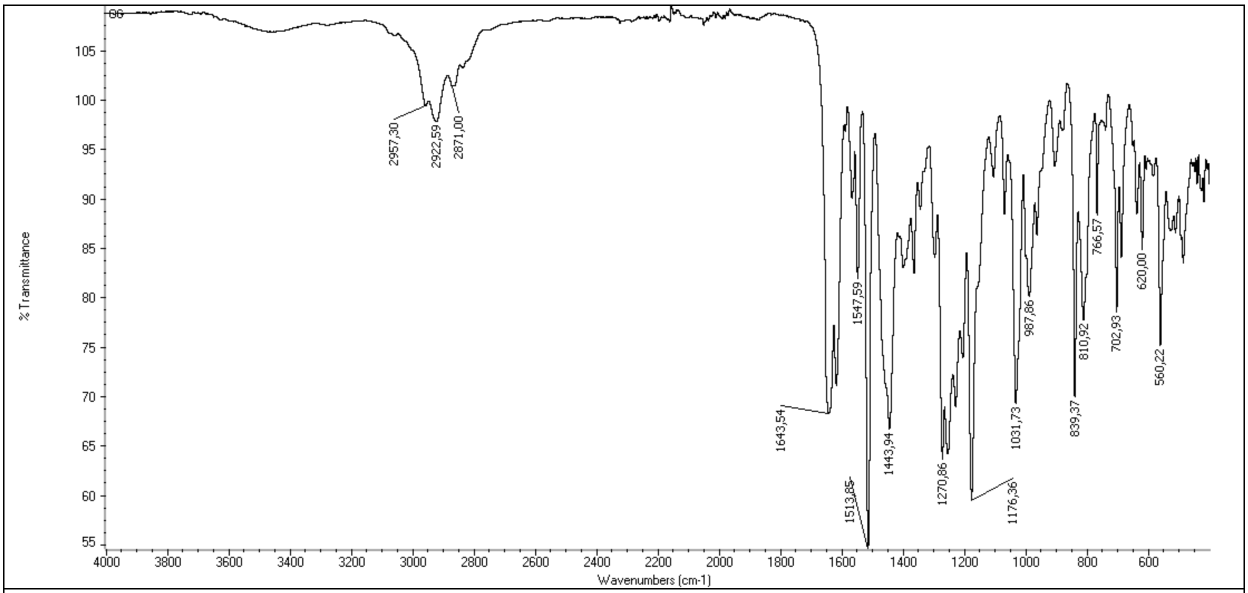
% Transmittance
Wavenumbers (cm-1)
2957,30
2922,59
2871,00
1643,54
1547,59
1513,85
1443,94
1270,86
1176,36
1031,73
987,86
839,37
810,92
766,57
702,93
620,00
560,22

9c

Table S5 – Molecular formula strings (CSV) of reported compounds

	SMILE
**2a**	CCCCn1c(c2c(c1C)c(=O)n(nc2OCC(=O)NNC(=S)NC)C)C
**2b**	CCCCn1c(c2c(c1C)c(=O)n(nc2OCC(=O)NNC(=S)Nc3ccccc3)C)C
**2c**	CCCCn1c(c2c(c1C)c(=O)n(nc2OCC(=O)NNC(=S)Nc3ccc(cc3)OC)C)C
**3a**	CCCCn1c(c2c(c1C)c(=O)n(nc2OCc3n[nH]c(=S)n3C)C)C
**3b**	CCCCn1c(c2c(c1C)c(=O)n(nc2OCc3n[nH]c(=S)n3c4ccccc4)C)C
**3c**	CCCCn1c(c2c(c1C)c(=O)n(nc2OCc3n[nH]c(=S)n3c4ccc(cc4)OC)C)C
**4a**	CCCCn1c(c2c(c1C)c(=O)n(nc2OCc3nn(c(=S)n3C)CN4CCN(CC4)c5ccccc5)C)C
**5a**	CCCCn1c(c2c(c1C)c(=O)n(nc2OCc3nn(c(=S)n3C)CN4CCN(CC4)c5cccc(c5)C(F)(F)F)C)C
**6a**	CCCCn1c(c2c(c1C)c(=O)n(nc2OCc3nn(c(=S)n3C)CN4CCN(CC4)c5ccc(cc5)C)C)C
**7a**	CCCCn1c(c2c(c1C)c(=O)n(nc2OCc3nnc(n3C)SCC(=O)N4CCN(CC4)c5ccccc5)C)C
**8a**	CCCCn1c(c2c(c1C)c(=O)n(nc2OCc3nnc(n3C)SCC(=O)N4CCN(CC4)c5cccc(c5)C(F)(F)F)C)C
**9a**	CCCCn1c(c2c(c1C)c(=O)n(nc2OCc3nnc(n3C)SCC(=O)N4CCN(CC4)c5ccc(cc5)C)C)C
**4b**	CCCCn1c(c2c(c1C)c(=O)n(nc2OCc3nn(c(=S)n3c4ccccc4)CN5CCN(CC5)c6ccccc6)C)C
**5b**	CCCCn1c(c2c(c1C)c(=O)n(nc2OCc3nn(c(=S)n3c4ccccc4)CN5CCN(CC5)c6cccc(c6)C(F)(F)F)C)C
**6b**	CCCCn1c(c2c(c1C)c(=O)n(nc2OCc3nn(c(=S)n3c4ccccc4)CN5CCN(CC5)c6ccc(cc6)C)C)C
**7b**	CCCCn1c(c2c(c1C)c(=O)n(nc2OCc3nnc(n3c4ccccc4)SCC(=O)N5CCN(CC5)c6ccccc6)C)C
**8b**	CCCCn1c(c2c(c1C)c(=O)n(nc2OCc3nnc(n3c4ccccc4)SCC(=O)N5CCN(CC5)c6cccc(c6)C(F)(F)F)C)C
**9b**	CCCCn1c(c2c(c1C)c(=O)n(nc2OCc3nnc(n3c4ccccc4)SCC(=O)N5CCN(CC5)c6ccc(cc6)C)C)C
**4c**	CCCCn1c(c2c(c1C)c(=O)n(nc2OCc3nn(c(=S)n3c4ccc(cc4)OC)CN5CCN(CC5)c6ccccc6)C)C
**5c**	CCCCn1c(c2c(c1C)c(=O)n(nc2OCc3nn(c(=S)n3c4ccc(cc4)OC)CN5CCN(CC5)c6cccc(c6)C(F)(F)F)C)C
**6c**	CCCCn1c(c2c(c1C)c(=O)n(nc2OCc3nn(c(=S)n3c4ccc(cc4)OC)CN5CCN(CC5)c6ccc(cc6)C)C)C
**7c**	CCCCn1c(c2c(c1C)c(=O)n(nc2OCc3nnc(n3c4ccc(cc4)OC)SCC(=O)N5CCN(CC5)c6ccccc6)C)C
**8c**	CCCCn1c(c2c(c1C)c(=O)n(nc2OCc3nnc(n3c4ccc(cc4)OC)SCC(=O)N5CCN(CC5)c6cccc(c6)C(F)(F)F)C)C
**9c**	CCCCn1c(c2c(c1C)c(=O)n(nc2OCc3nnc(n3c4ccc(cc4)OC)SCC(=O)N5CCN(CC5)c6ccc(cc6)C)C)C

Table S6 – CD spectra of BSA in the absence and presence of all analyzed compounds

BSA to analyzed compounds' molar ratios was changing from 1:0 to 1:10

<table>
<tr><td>a) 4a
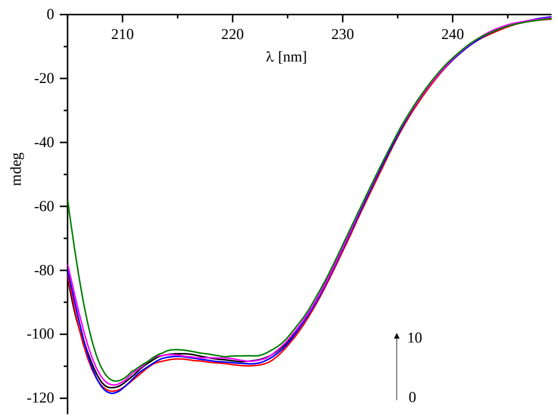</td><td>b) 5a
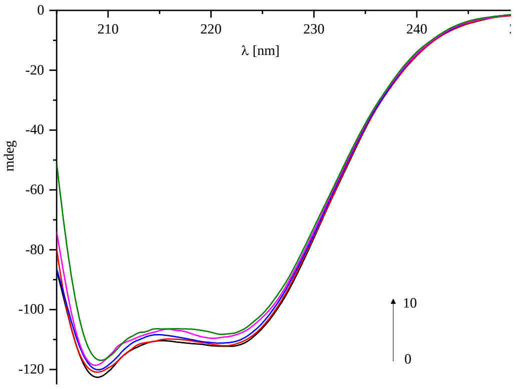</td></tr>
<tr><td>c) 6a
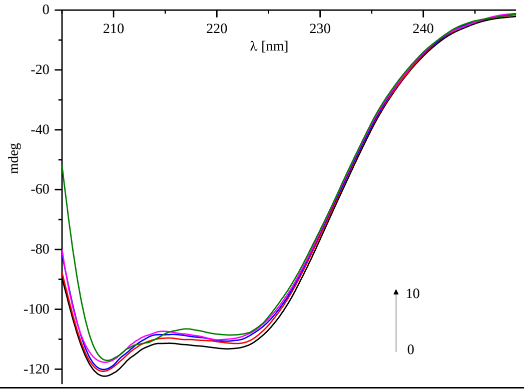</td><td>d) 7a
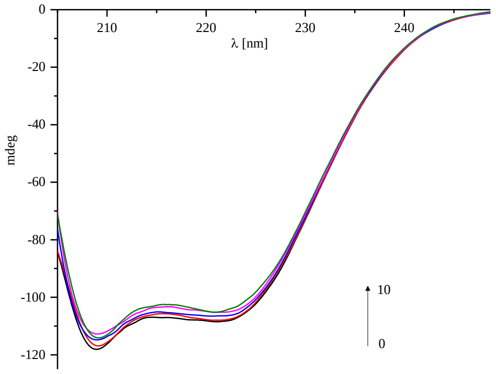</td></tr>
<tr><td>e) 9a
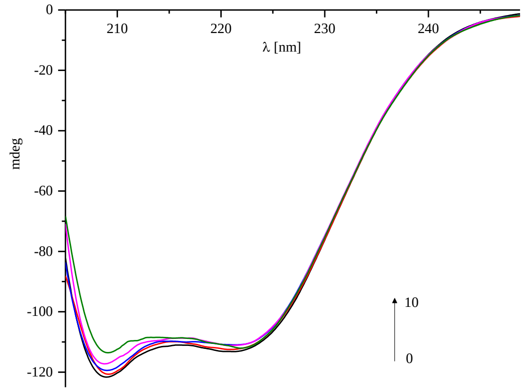</td><td>f) 4b
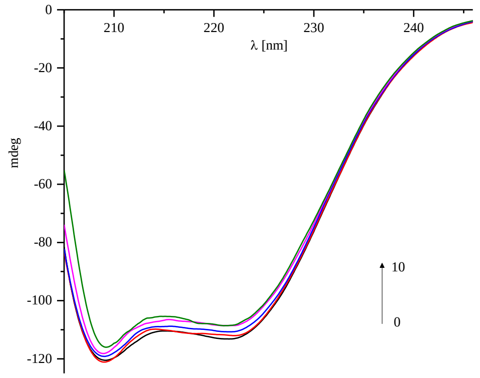</td></tr>
<tr><td>g) 5b</td><td>h) 6b</td></tr>
</table>

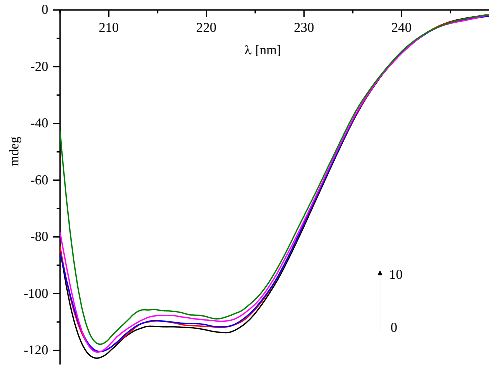

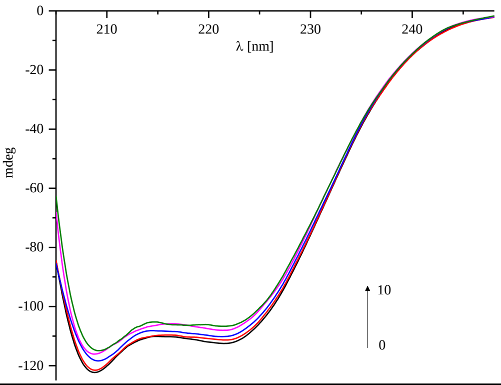

i) **7b**

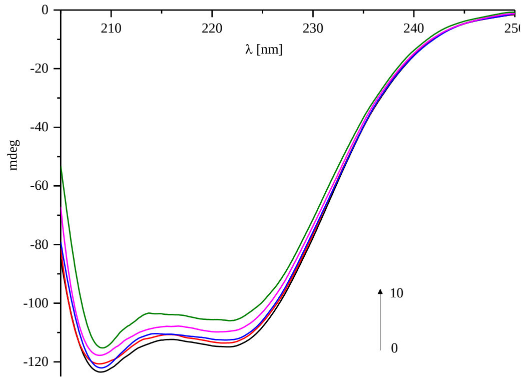

j) **4c**

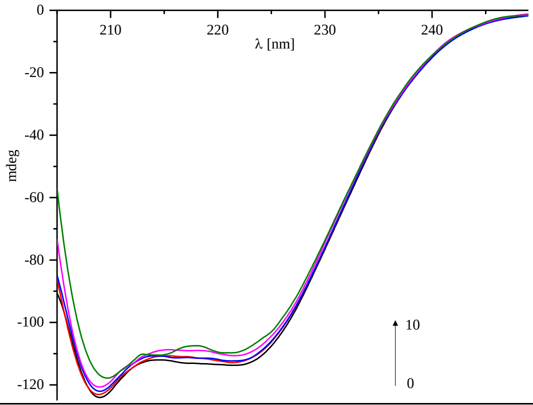

k) **5c**

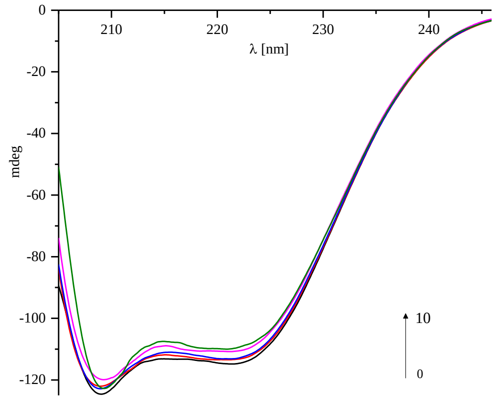

l) **6c**

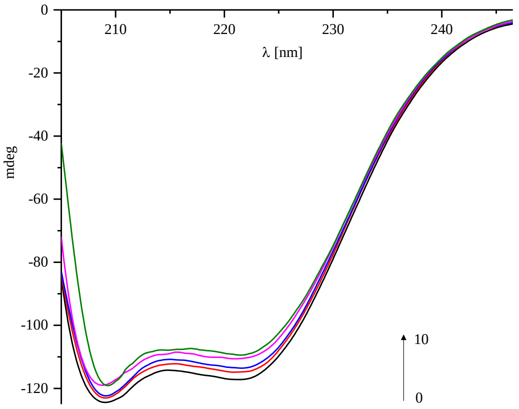

m) **7c**

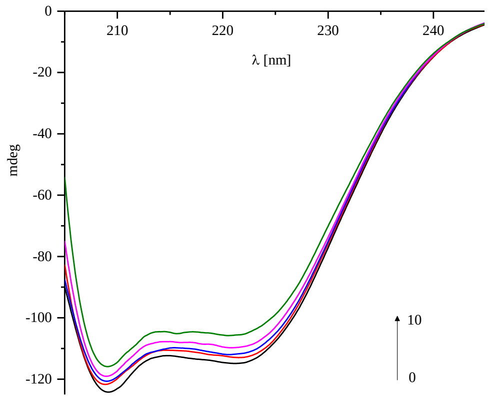

Table S7 - FT-IR spectra - The curve-fitted Amide I region

The curve-fitted Amide I region for a) free-BSA and equimolar complex samples: b) BSA-**4a**, c) BSA-**5a**, d) BSA-**6a**, e) BSA-**7a**, f) BSA-**9a**, g) BSA-**4b**, h) BSA-**5b**, i) BSA-**6b**, j) BSA-**7b**, k) BSA-**4c**, l) BSA-**5c**, m) BSA-**6c**, n) BSA-**7c**.

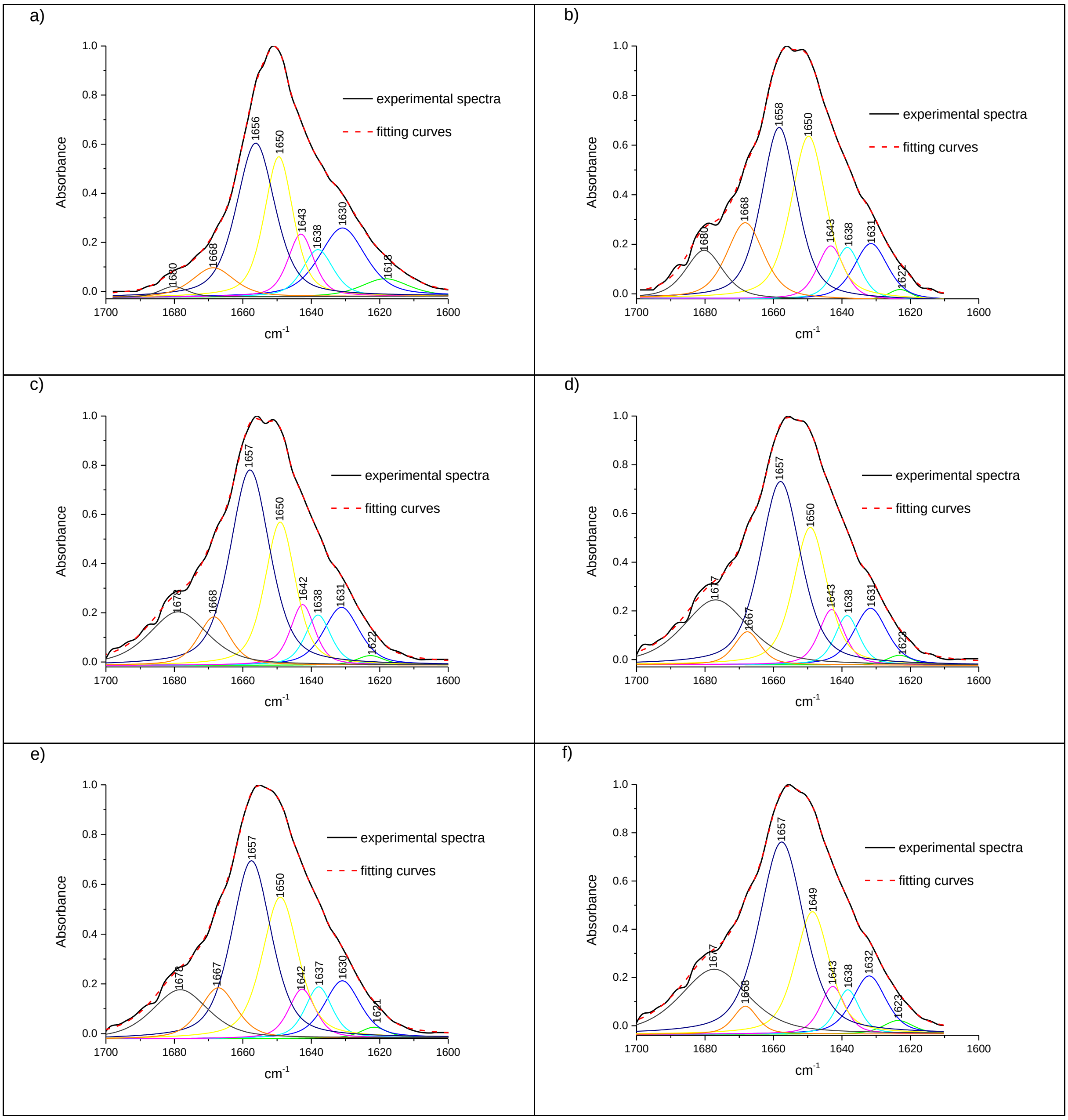

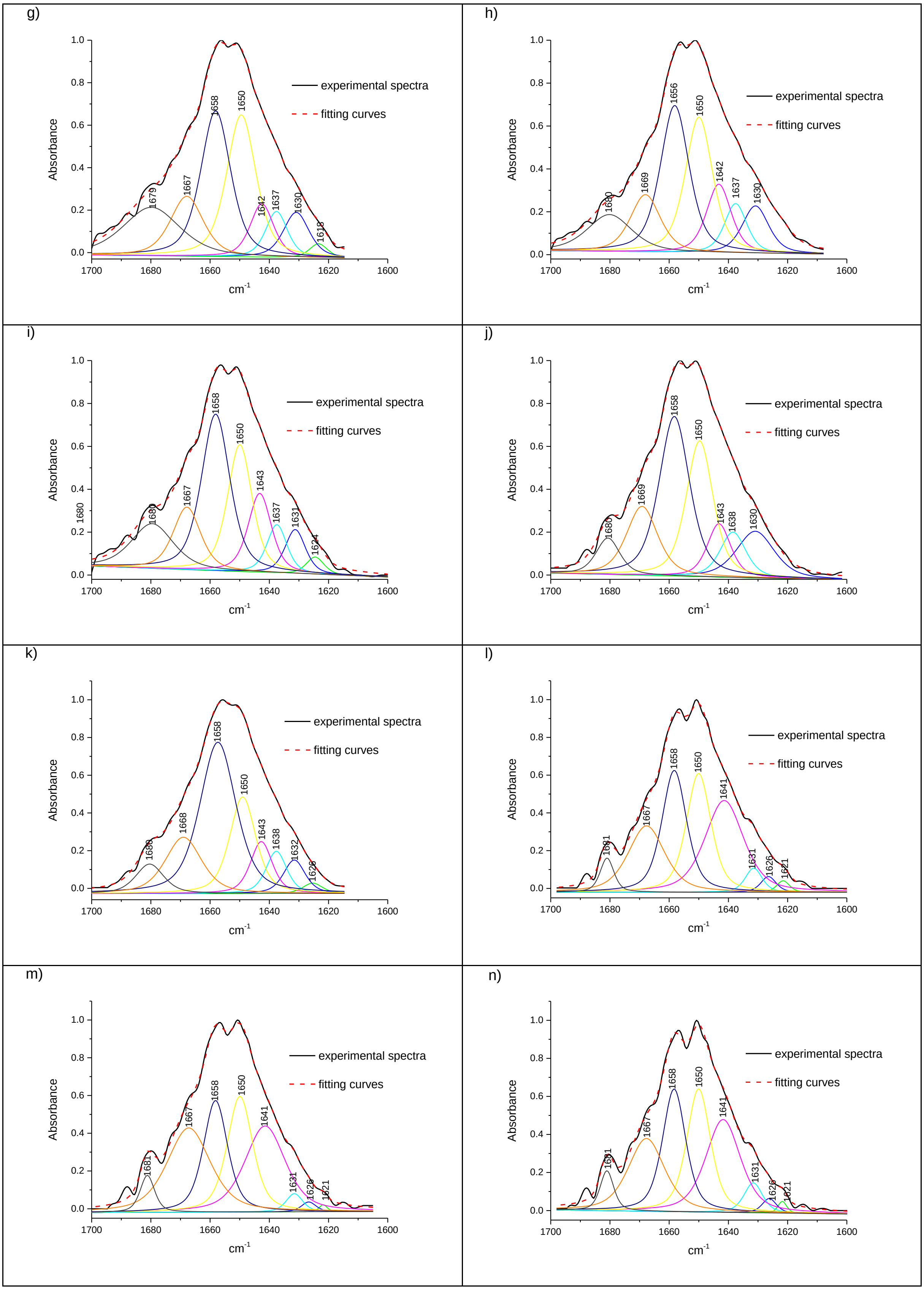

g)
Absorbance
experimental spectra
fitting curves
1679
1667
1658
1650
1642
1637
1630
1618
cm-1
h)
Absorbance
experimental spectra
fitting curves
1680
1669
1656
1650
1642
1637
1630
cm-1
i)
Absorbance
experimental spectra
fitting curves
1680
1667
1658
1650
1643
1637
1631
1624
cm-1
j)
Absorbance
experimental spectra
fitting curves
1680
1669
1658
1650
1643
1638
1630
cm-1
k)
Absorbance
experimental spectra
fitting curves
1680
1668
1658
1650
1643
1638
1632
1626
cm-1
l)
Absorbance
experimental spectra
fitting curves
1681
1667
1658
1650
1641
1631
1626
1621
cm-1
m)
Absorbance
experimental spectra
fitting curves
1681
1667
1658
1650
1641
1631
1626
1621
cm-1
n)
Absorbance
experimental spectra
fitting curves
1681
1667
1658
1650
1641
1631
1626
1621
cm-1

Table S8 - FT-IR spectra – The changes in Amide I (1650cm^{-1}) and Amide II (1545cm^{-1}) band contribution by BSA interaction with analyzed compounds.

a) **4a**, b) **5a**, c) **6a**, d) **7a**, e) **9a**, f) **4b**, g) **5b**, h) **6b**, i) **7b**, j) **4c**, k) **5c**, l) **6c**, m) **7c**.

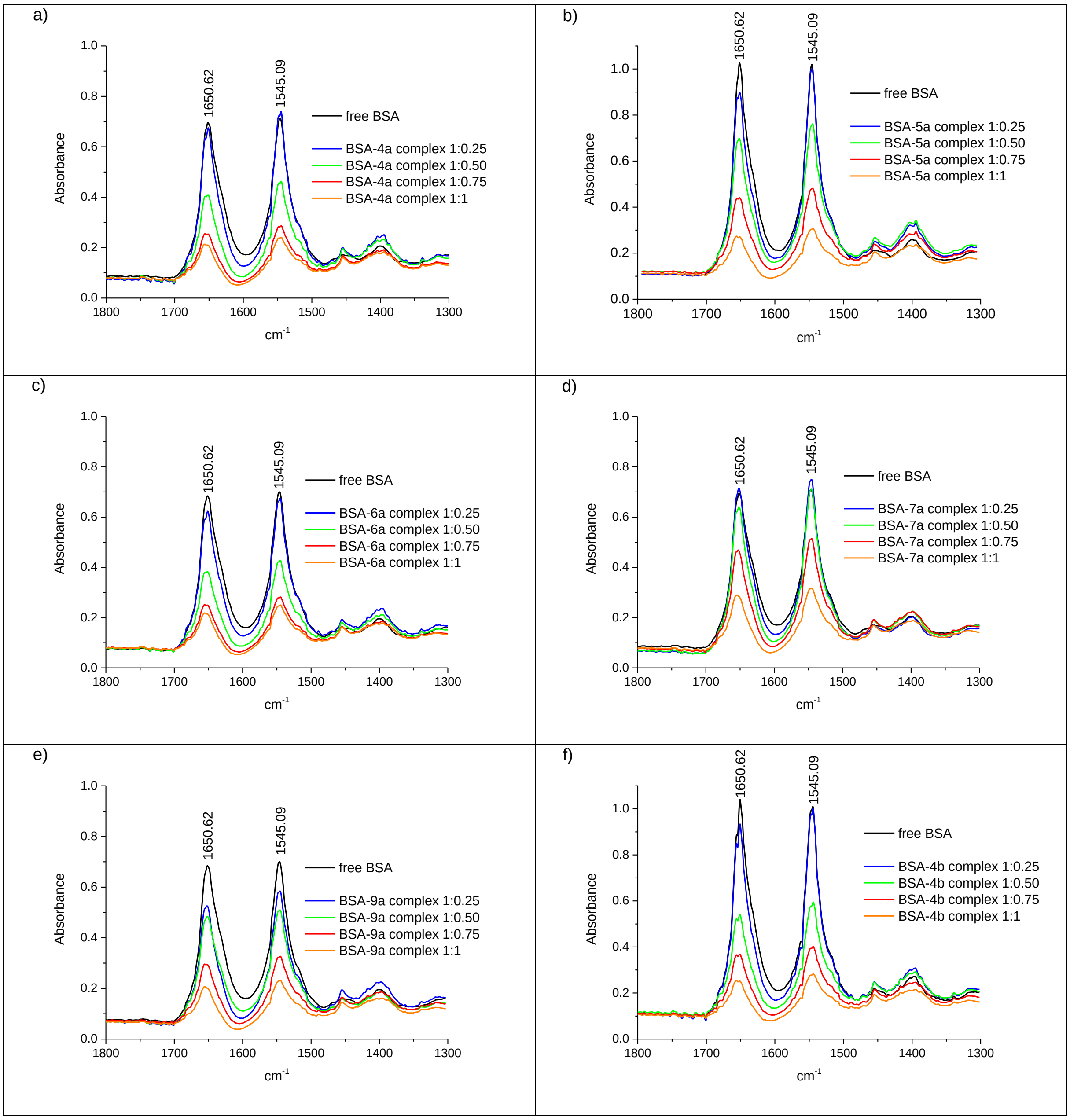

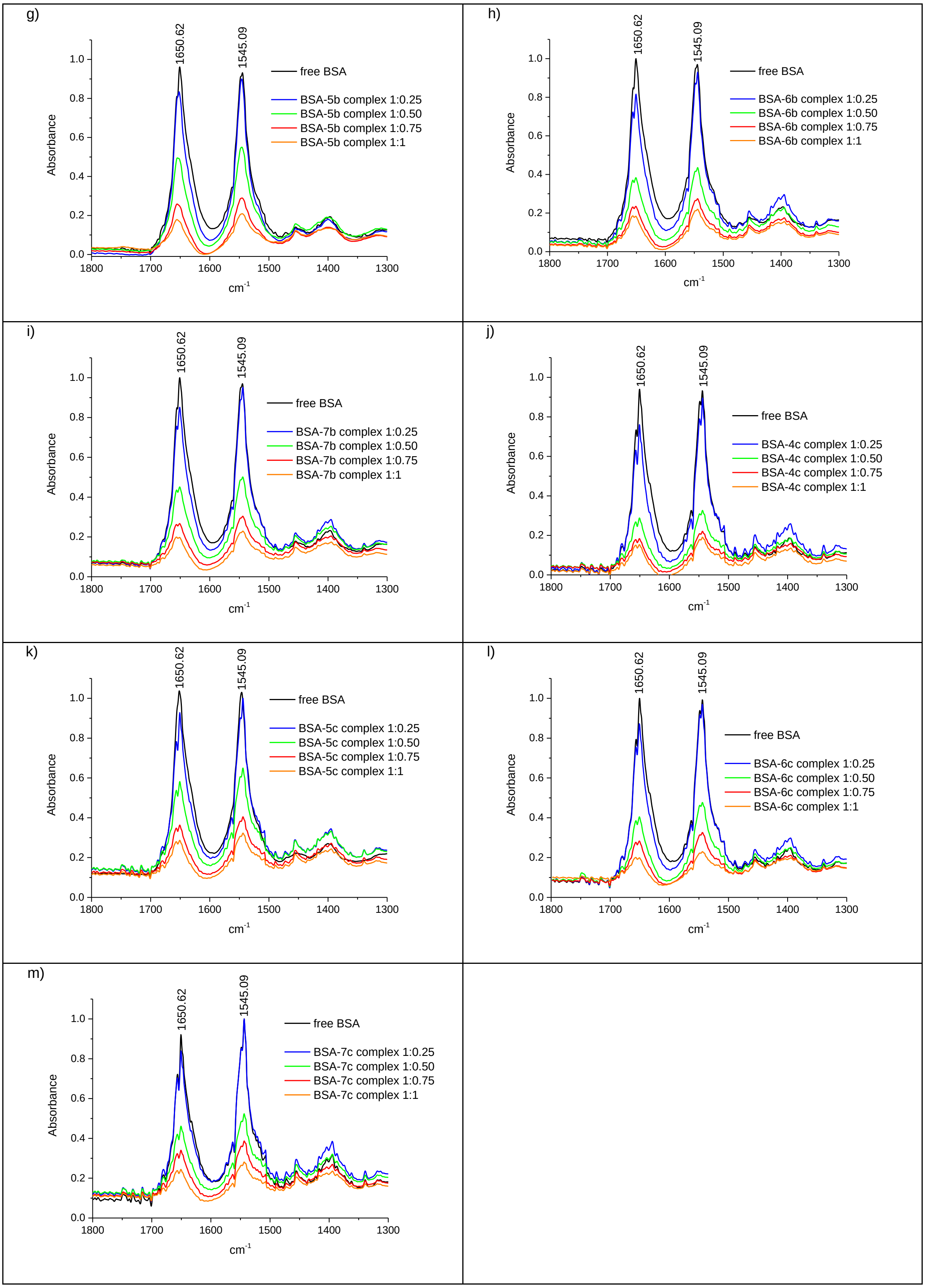

g)
1650.62
1545.09
Absorbance
cm-1
free BSA
BSA-5b complex 1:0.25
BSA-5b complex 1:0.50
BSA-5b complex 1:0.75
BSA-5b complex 1:1
h)
1650.62
1545.09
Absorbance
cm-1
free BSA
BSA-6b complex 1:0.25
BSA-6b complex 1:0.50
BSA-6b complex 1:0.75
BSA-6b complex 1:1
i)
1650.62
1545.09
Absorbance
cm-1
free BSA
BSA-7b complex 1:0.25
BSA-7b complex 1:0.50
BSA-7b complex 1:0.75
BSA-7b complex 1:1
j)
1650.62
1545.09
Absorbance
cm-1
free BSA
BSA-4c complex 1:0.25
BSA-4c complex 1:0.50
BSA-4c complex 1:0.75
BSA-4c complex 1:1
k)
1650.62
1545.09
Absorbance
cm-1
free BSA
BSA-5c complex 1:0.25
BSA-5c complex 1:0.50
BSA-5c complex 1:0.75
BSA-5c complex 1:1
l)
1650.62
1545.09
Absorbance
cm-1
free BSA
BSA-6c complex 1:0.25
BSA-6c complex 1:0.50
BSA-6c complex 1:0.75
BSA-6c complex 1:1
m)
1650.62
1545.09
Absorbance
cm-1
free BSA
BSA-7c complex 1:0.25
BSA-7c complex 1:0.50
BSA-7c complex 1:0.75
BSA-7c complex 1:1
